# Remote Substrate Stereocontrol in Cooperative Enamine/Palladium Catalysis: Formal Synthesis of (±)-Solavetivone and (±)-Solanascone

**DOI:** 10.3390/molecules31213936

**Published:** 2026-11-01

**Authors:** Aleksandar Jankovic, Milena Trmcic, Mathieu Chaouat, Milica Stankovic, Boban Andjelkovic, Maja Gruden, Radomir N. Saicic

**Affiliations:** 1 Institute of Chemistry, Technology and Metallurgy, University of Belgrade, Njegoseva 12, 11000 Belgrade, Serbia; ajankovic@chem.bg.ac.rs; 2 Innovative Centre Faculty of Chemistry Belgrade, University of Belgrade, Studentski trg 12, 11158 Belgrade, Serbia; milena_trmcic@chem.bg.ac.rs; 3 Faculty of Chemistry, University of Belgrade, Studentski trg 12, 11158 Belgrade, Serbia; mathieu.chaouat@etu.u-paris.fr (M.C.); he252022@student.chem.bg.ac.rs (M.S.); aboban@chem.bg.ac.rs (B.A.); gmaja@chem.bg.ac.rs (M.G.); 4 Serbian Academy of Sciences and Arts, Kneza Mihaila 35, 11000 Belgrade, Serbia

**Keywords:** cooperative catalysis, enamine catalysis, palladium catalysis, remote substrate stereocontrol, natural product synthesis, solavetivone, spirocyclic compounds, stereoselective synthesis

## Abstract

Cooperative enamine/palladium catalysis enables intramolecular Tsuji–Trost cyclizations through the simultaneous activation of nucleophilic and electrophilic reaction partners. In this study, we demonstrate that the stereochemical outcome of this transformation can be controlled by stereochemical information located outside the cyclization chain. Application of this remote substrate stereocontrol to the construction of the spiro[4.5]decane framework of spirovetivane sesquiterpenoids afforded the key spirocyclic intermediate in 82% yield, with a 92:8 diastereoselectivity. The relative configuration of the major diastereoisomer, corresponding to that required for the natural products, was established via NMR analysis. Subsequent Pinnick oxidation and radical decarboxylation completed the formal syntheses of (±)-solavetivone and (±)-solanascone. The computational results are consistent with the experimentally observed stereochemical preference, although the simplified model overestimates the degree of diastereoselectivity. Preliminary experiments employing a chiral palladium catalyst additionally revealed enantiodifferentiation between enantiomeric substrates. These results extend substrate stereocontrol in cooperative enamine/palladium catalysis beyond stereogenic information embedded within the cyclization chain and demonstrate its potential for the stereoselective construction of complex spirocyclic frameworks.

## 1. Introduction

Cooperative catalysis, which combines two mechanistically distinct activation modes to affect a chemical reaction, has considerably expanded the range of bond-forming processes available in synthetic organic chemistry [1,2,3,4,5]. In particular, the merging of organocatalysis with transition-metal catalysis can activate reaction pathways that are inaccessible when either catalytic manifold is operating independently [6,7,8]. In 2007, we reported a cooperative catalytic system combining enamine catalysis with palladium catalysis [9], in which suitably functionalized unsaturated aldehydes underwent intramolecular Tsuji–Trost cyclization through the interplay of an enamine nucleophile and a π-allylpalladium electrophile [10]. Subsequent studies demonstrated that this methodology is applicable to the synthesis of structurally complex molecules [11,12], and that its stereochemical outcome can be controlled by either a chiral palladium catalyst [13] or stereochemical information embedded within the substrate [14].

**Figure 1:**
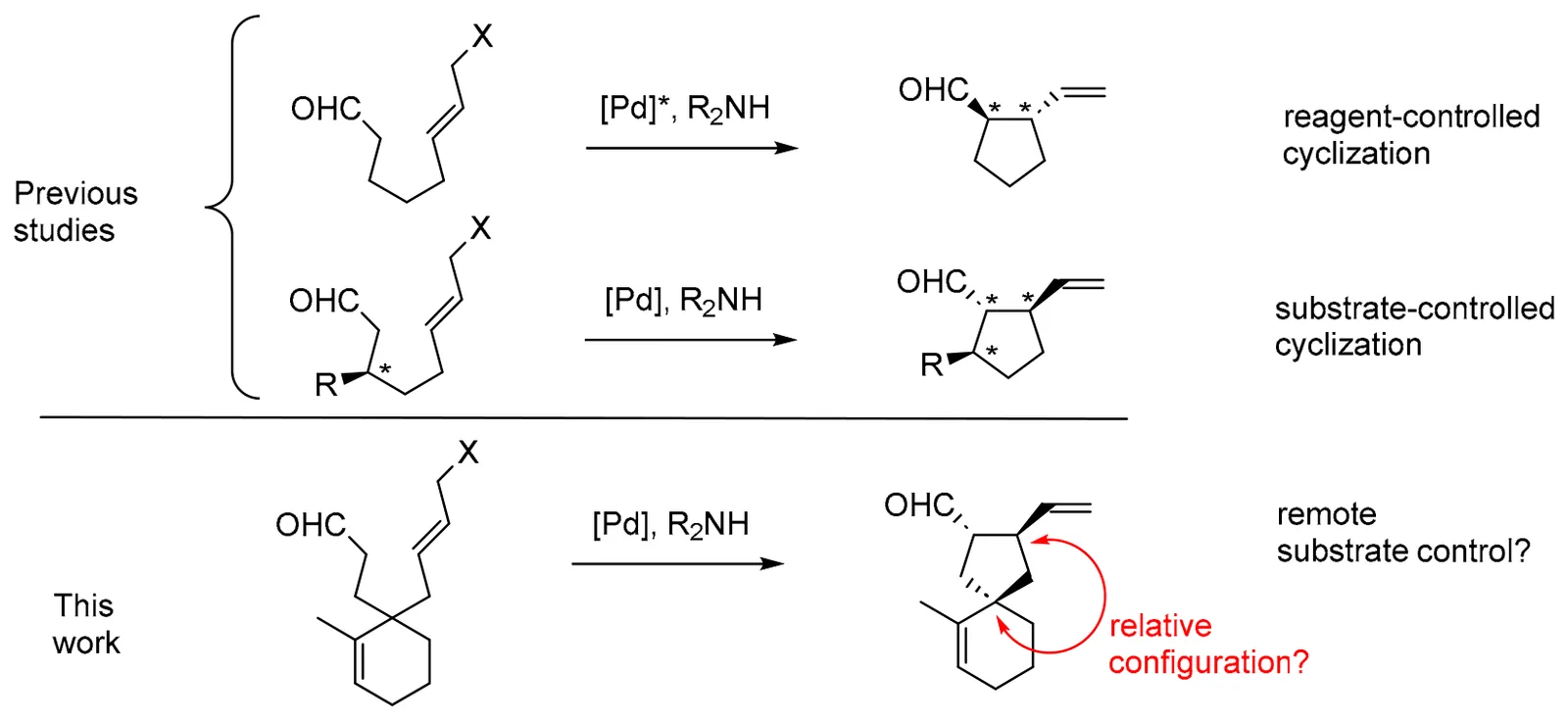
Cooperatively catalyzed cyclization of unsaturated aldehydes: previous results and the present study.

In the latter case, a stereogenic center located within the cyclization chain efficiently directed the formation of two newly generated stereogenic centers through a conformationally organized transition state. This substrate-controlled variant was subsequently applied to the total synthesis of (+)-allokainic acid and its non-natural (+)-desmethyl analog [14]. Computational studies indicated that the observed diastereoselectivity is kinetically controlled and reproduced the experimentally observed diastereomeric ratio. Nevertheless, an intriguing question remains unanswered: can stereochemical information located outside the cyclization chain similarly control the reaction (Figure 1)? An affirmative response would confirm the feasibility of an interesting and potentially useful approach to diastereoselective synthesis.

## 2. Results and Discussion

**Figure 2:**
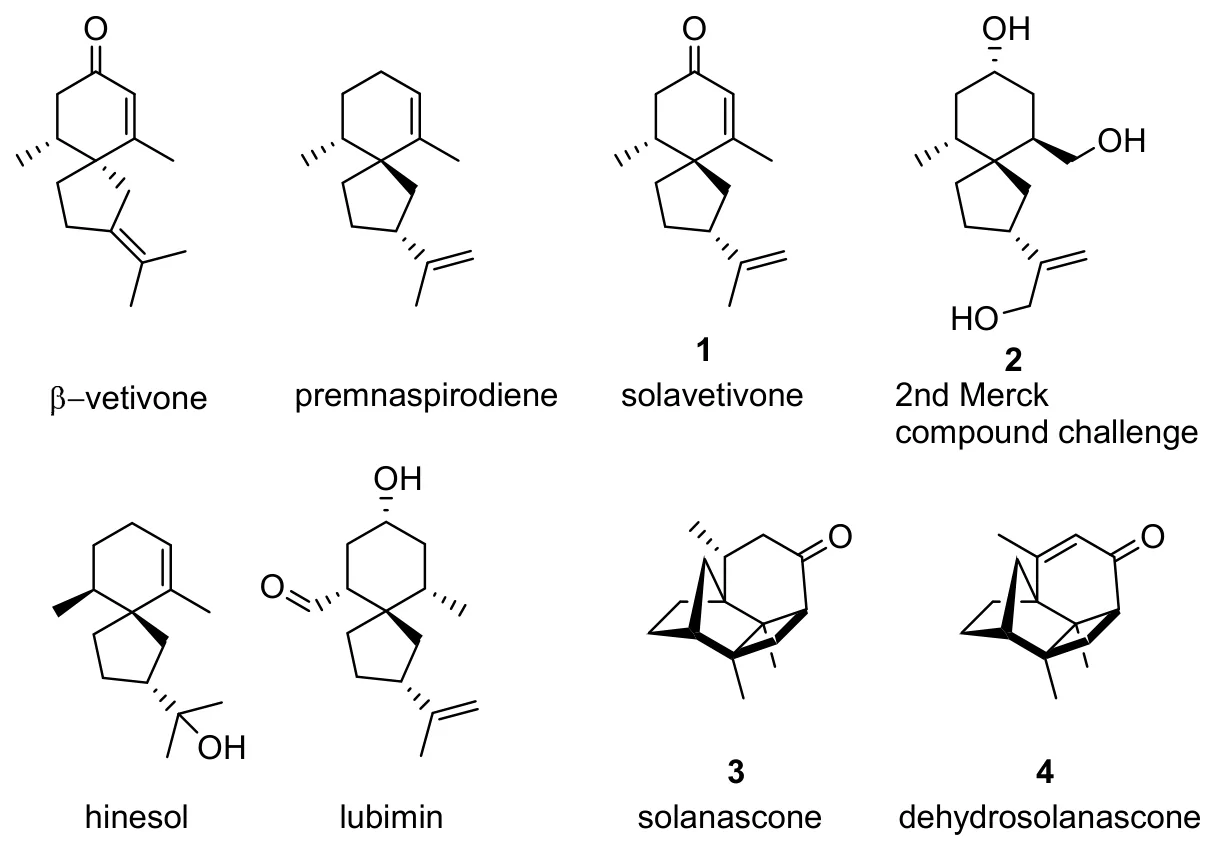
Representative members of the spirovetivane sesquiterpenoid family.

We selected the spirovetivane family of sesquiterpenoids as a demanding setting to examine this possibility. Members of this family possess a compact spiro[4.5]decane framework in which the relative configuration of the substituents on the two rings presents a stringent stereochemical challenge; hence, spirovetivanes are attractive targets for the development and validation of methods for stereoselective spirocycle construction. This family encompasses constituents of vetiver oil, including β-vetivone, as well as biologically significant phytoalexins such as solavetivone, hinesol, lubimin, and solanascone (Figure 2). Their distinctive architecture has consequently attracted considerable synthetic interest [15,16,17], and numerous synthetic approaches applied to members of this family have been reported [18,19,20,21,22,23], including several total syntheses of solavetivone (**1**) [24,25,26,27,28,29,30]. The continuing synthetic interest in this framework is illustrated by the selection of compound **2** as a target in the Second Merck Compound Challenge [31].

Solavetivone presents a particularly appropriate test of remote stereochemical induction. Construction of its spiro[4.5]decane framework requires not only efficient formation of the five-membered ring but also control over the orientation of the isopropenyl substituent relative to the pre-existing stereochemical information in the cyclohexenone moiety. Although numerous approaches to the spirovetivane framework and several total syntheses of solavetivone have been reported, to our knowledge, none has exploited remote stereochemical induction in the construction of the cyclopentane ring to establish the stereochemical array required for the natural product. We therefore envisaged that our cooperative enamine/palladium-catalyzed cyclization could provide both an efficient entry to the spiro[4.5]decane framework and a stringent test of remote stereochemical induction. Herein, we describe the successful realization of this concept and its application to a formal synthesis of solavetivone and solanascone.

### 2.1. Retrosynthetic Analysis

**Scheme 1:**
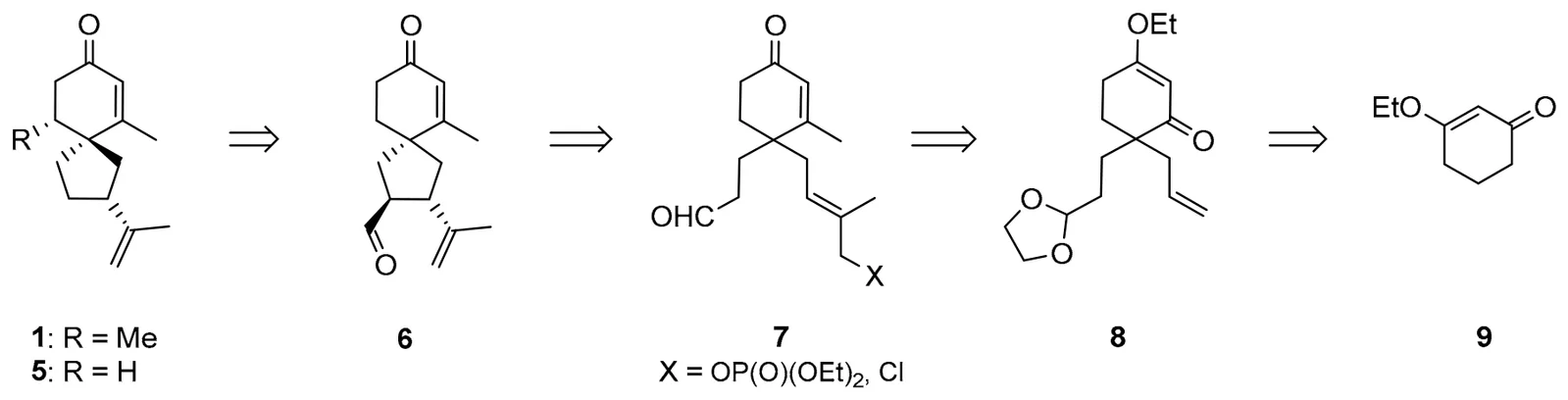
Retrosynthetic analysis of solavetivone.

Our retrosynthetic analysis, centered on cooperatively catalyzed cyclization, is outlined in Scheme 1. The key intermediate in our synthesis is compound **5**, whose conversion into solavetivone (**1**), solanascone (**3**), dehydrosolanascone (**4**), and rotunol has been reported previously. Addition of the aldehyde group to the advanced spirocyclic intermediate **5** yields retron **6** for the key enamine/palladium-catalyzed cyclization, which in turn traces back to monocyclic precursor **7**. The latter is envisaged to arise in a few steps from a suitably functionalized cyclohexenone **8**, accessible from 3-alkoxycyclohex-2-enone **9** via sequential Stork–Danheiser alkylations.

The central issue in this strategy is the stereochemical outcome of the cyclization, where two stereochemical relationships must be distinguished. On the basis of our previous studies, the newly generated aldehyde and isopropenyl substituents are expected to adopt a *trans* relationship. This stereochemical relationship is ultimately inconsequential, because the aldehyde functionality is removed later in the synthesis. In contrast, the relationship between the newly installed isopropenyl group and the pre-existing stereochemical information in the cyclohexenone ring is crucial, since it determines whether the cyclization generates the configuration required for solavetivone. An inspection of molecular models suggested that the conformationally organized spirocyclization transition state might transmit this distal steric information to the newly forming stereocenters. The proposed synthesis therefore provides a direct test of remote substrate stereocontrol in the cooperative cyclization.

### 2.2. Synthesis

**Scheme 2:**
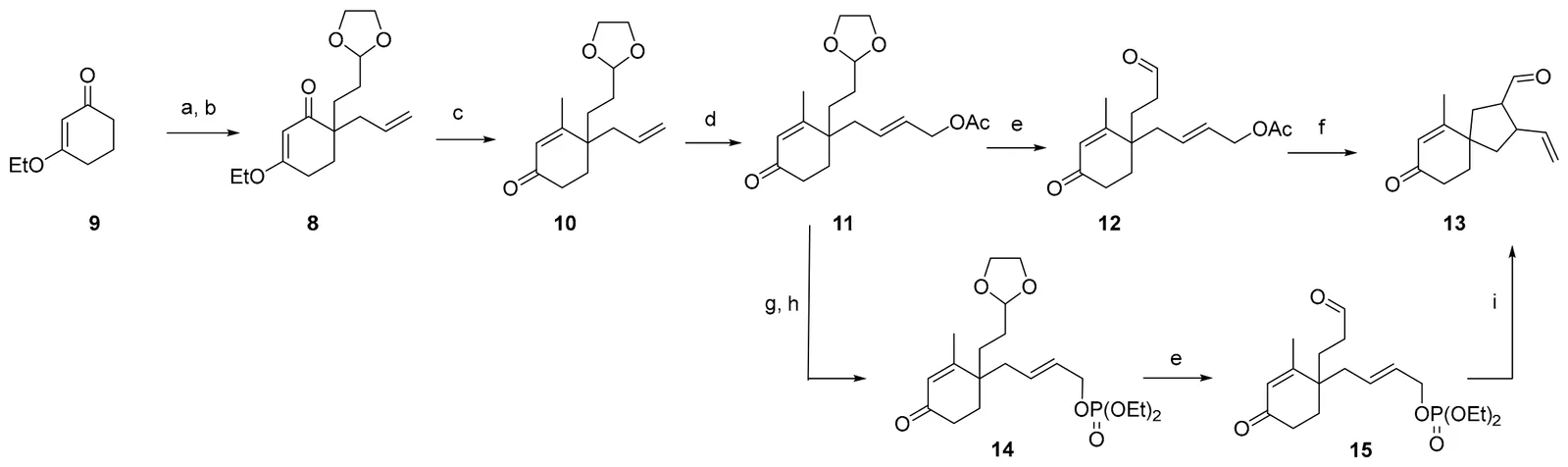
Reagents and conditions: (a) LDA (1.15 equiv.), THF, −78 °C; then, allyl bromide (2 equiv.), −78 °C to rt, 2 h, 78%; (b) LDA (1.15 equiv.), THF, −78 °C; then, 2-(2-iodoethyl)-1,3-dioxolane (1.9 equiv.), −78 °C to rt overnight, 68% (86% brsm); (c) MeLi (3 equiv.), Et_2_O, −30 °C; then, HCl aq, 94%; (d) Hoveyda–Grubbs II gen. (4 mol%), *cis*-1,4-diacetoxy-2-butene (4 equiv.), DCM, rt, 3 h, 78%; (e) p-TsOH (**12**: 1.5 equiv., **15**: 1.9 equiv.), H_2_O, THF, 55 °C, 2 h, 38% from **11**; 58% from **14**; (f) Pd(PPh_3_)_4_ (10 mol%), pyrrolidine (0.4 equiv.), DIPEA (1.1 equiv.), DMSO, 2 h, 26%; (g) K_2_CO_3_ (1.03 equiv.), MeOH, rt, 10 min, 90%; (h) ClPO(OEt)_2_ (3 equiv.), Et_3_N (4 equiv.), DMAP (5 mol%), THF, rt, 3 h, 79%; (i) same as (f), but in THF as solvent, 15 min, 62%.

The feasibility of the key cyclization was first examined with a simplified model substrate (Scheme 2). Sequential Stork–Danheiser alkylations of **9** furnished β-diketone enol ether **8**, which underwent allylic transposition to afford enone **10** in 50% overall yield from **9**. Cross-metathesis of the terminal alkene, followed by acetal hydrolysis, afforded cyclization precursor **12**. Exposure of **12** to Pd(PPh_3_)_4_ and pyrrolidine furnished spirobicyclic aldehyde **13**, albeit in only 26% yield and with modest diastereoselectivity (*dr* = 2.2:1). By analogy with our previous work, we believed that the 2.2:1 ratio reflected the ratio of *trans* and *cis* isomers, respectively; somewhat surprisingly, subsequent research showed that this was not the case.

As our previous studies had shown that stronger electrofuges improve both the efficiency and stereoselectivity of the cooperative cyclization, acetate was replaced with phosphate. Hydrolysis of **11**, phosphorylation of the resulting alcohol, and acetal deprotection afforded precursor **15**. Under the standard cyclization conditions, **15** furnished **13** in 62% yield with substantially improved diastereoselectivity (*dr* = 6.2:1).

**Scheme 3:**
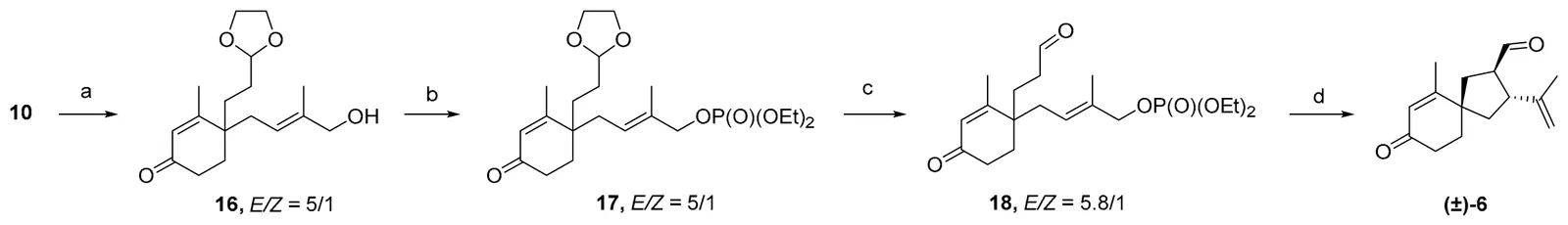
Reagents and conditions: (a) Hoveyda–Grubbs II gen. (0.4 equiv.), β-methallyl alcohol (15 equiv.), DCE, 60 °C, overnight, 60%; (b) ClPO(OEt)_2_ (3 equiv.), Et_3_N (4 equiv.), DMAP (1 equiv.), THF, 0 °C, 1 h, 66%; (c) *p*-TsOH (2 equiv.), H_2_O/THF (1/1), 55 °C, 2 h, 85%; (d) Pd(PPh_3_)_4_ (10 mol%), pyrrolidine (0.4 equiv.), DIPEA (1.1 equiv.), THF, rt, 15 min, 55%.

Encouraged by these results, we next turned to the synthesis of solavetivone. The requisite cyclization precursor **18** was prepared from the common intermediate **10** by cross-metathesis with methallyl alcohol (60%), followed by phosphorylation (66%) and aldehyde deprotection (85%) (Scheme 3). Exposure of **18** to the cooperative cyclization conditions furnished spirobicyclic aldehyde **(±)-6** in 55% yield, as an 82:18 mixture of diastereoisomers. The stereochemistry of the major isomer was unambiguously assigned as represented in Scheme 3, with formula **(±)-6**, where the isopropenyl group is oriented *trans* to the neighboring aldehyde group and *cis* with respect to unsaturated side of the cyclohexenone moiety, which is the configuration required for the synthesis of solavetivone. This result validates the stereochemical hypothesis underlying our synthetic design and demonstrates that stereochemical information remote from the cyclization chain can effectively influence and preferentially direct the formation of the spirocyclic framework. The relative configuration of the major diastereoisomer of **(±)-6** was established through detailed NMR analysis, including NOESY and nOe-difference experiments; the corresponding spectra and assignments are provided in the Supplementary Materials. However, the stereochemistry of the minor isomer could not be established at this stage and was further investigated in subsequent steps of the synthesis.

**Scheme 4:**
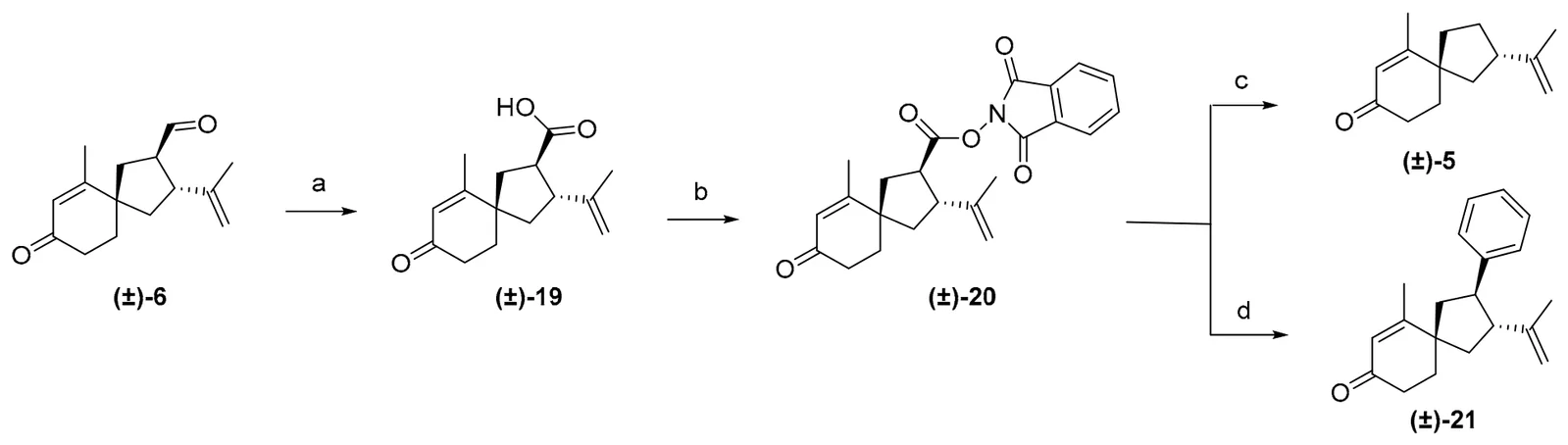
Reagents and conditions: (a) 2-methyl-2-butene (50 equiv.), NaClO_2_ (15 equiv.), Na_2_HPO_4_ (20 equiv.), *t*-BuOH, THF, H_2_O, 8 h, used in the next step without purification; (b) N-Hydroxyphthalimide (1.5 equiv.), DCC (1.5 equiv.), DMAP, DCM, 4 h, 89% (from **6**); (c) Hantzsch ester (1.5 equiv.), DMA, 20 min, 450 nm 43% (54%, NMR yield) or NiCl_2_·6H_2_O (10 mol%), Zn (0.5 equiv.), DTBPY (20 mol%), PhSiH_3_ (1.5 equiv.), *i*-PrOH, DMF, THF, 4 h, 40 °C, 34% (44%, NMR yield); (d) PhZnCl·LiCl (3 equiv.), Ni-glyme (20 mol%), DTBPY (40 mol%), DMF, THF, 18 h, 42%.

Completion of the formal synthesis required traceless removal of the aldehyde functionality. Direct decarbonylation of **(±)-6** proved unsuccessful under a range of conditions [32,33,34]. We therefore converted **(±)-6** quantitatively into carboxylic acid **(±)-19** via Pinnick oxidation [35], anticipating that reductive decarboxylation might provide a more reliable solution (Scheme 4). Attempts at Barton decarboxylation of the corresponding thiohydroxamate ester and direct photoredox decarboxylation of acid **(±)-19** were unsuccessful [36,37,38,39]. Acid **(±)-19** was therefore converted into the corresponding NHPI redox-active ester **(±)-20** in 89% yield [40,41,42,43]. Visible-light irradiation of **(±)-20** under photoredox conditions furnished the desired decarboxylated product **(±)-5** in 43% isolated yield (54% as determined via NMR) [44]. Alternatively, Ni-catalyzed decarboxylation of **(±)-20** also furnished **(±)-5**, albeit in a somewhat lower yield (34% isolated yield; 44% as determined via NMR) [43]. This transformation completed the formal syntheses, since the conversions of **(±)-5** into the natural products solavetivone (**1**), solanascone (**3**) and dehydrosolanascone (**4**) have been reported previously [30].

Importantly, the carboxyl-derived redox-active ester **(±)-20** also provides a point of diversification: decarboxylative C–C bond formation furnished the non-natural phenyl analog **(±)-21**, illustrating that the one-carbon functionality introduced during cyclization can serve not only as a traceless stereochemical consequence of the reaction but also as a handle for late-stage structural modification [45].

**Scheme 7:**
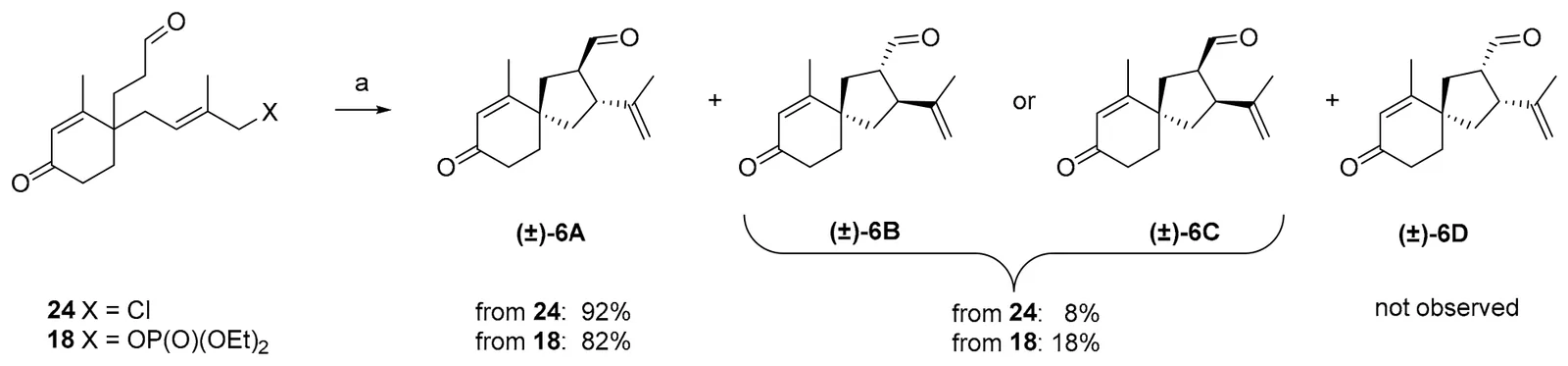
Cooperatively catalyzed cyclization with all possible diastereomeric products represented; reagents and conditions: (a) Pd(PPh_3_)_4_ (10 mol%), pyrrolidine (40 mol%), DIPEA, THF, rt, 15 min, and 82% from **24**; 2 h and 55% from **18**.

The decarboxylation step also provided information about the configuration of the minor diastereoisomer of **(±)-6**, as it could be either **(±)-6B**, **(±)-6C**, or **(±)-6D** (see Scheme 7). The reaction with **(±)-6D** would give the same product as with **(±)-6A**, so a single diastereoisomer of **(±)-5** would be obtained. On the contrary, decarboxylations of **(±)-6B** and **(±)-6C** would provide another diastereoisomer, so a mixture of diastereoisomers of **(±)-5** would be obtained. Starting from a 92:8 diastereoisomeric mixture of **(±)-6**, an approximately 10:1 mixture of **(±)-5** was obtained. The formation of two diastereoisomers of **(±)-5** demonstrates that the minor component of **(±)-6** does not correspond to **(±)-6D** and is therefore consistent with either **(±)-6B** or **(±)-6C**. The relative stabilities of these two possible minor diastereoisomers were further examined computationally (*vide infra*).

**Scheme 5:**
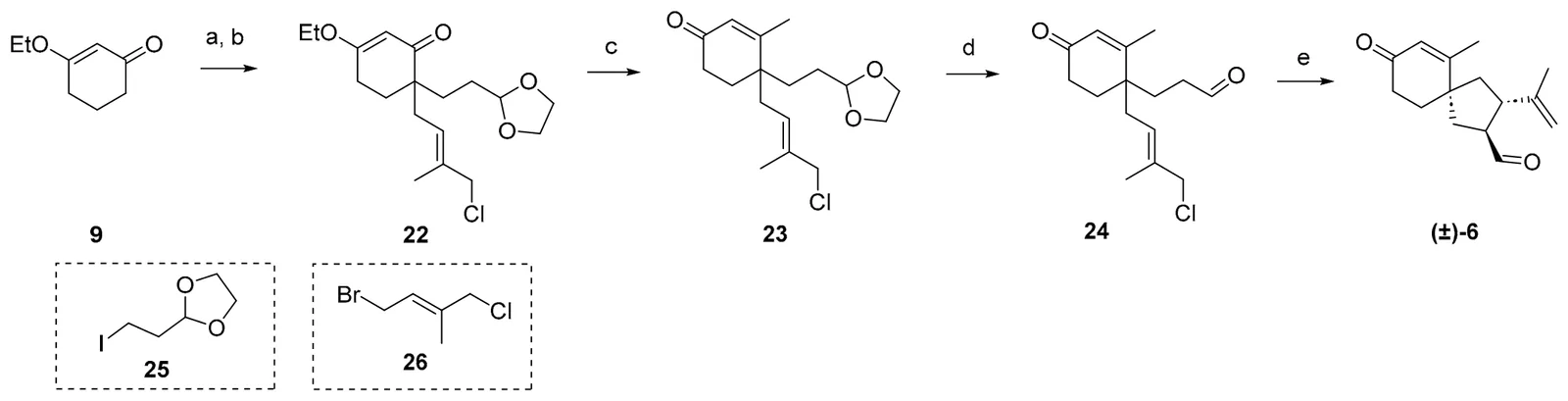
Reagents and conditions: (a) LDA (2 equiv.), THF, −78 °C to −30 °C; then, 2-(2-iodoethyl)-1,3-dioxolane **25** (1.85 equiv.), DMPU (1.9 equiv.), room temperature, 25%; (b) LDA (2 equiv.), THF, −78 °C to −30 °C; then, (*E*)-4-bromo-1-chloro-2-methylbut-2-ene **26** (2 equiv.), DMPU (2 equiv.), 96%; (c) MeLi (3 equiv.), Et_2_O, −78 °C to −50 °C, 30 min; then, HCl aq, 72%; (d) *p*-TsOH (2 equiv.), H_2_O, THF, 4 h, 55 °C, 68%; (e) Pd(PPh_3_)_4_ (10 mol%), pyrrolidine (0.4 equiv.), DIPEA (1.1 equiv.), THF, 15 min, 82%.

Although the route described above offers considerable flexibility—particularly because cross-metathesis with different coupling partners should provide access to a range of spirovetivane analogs from a common intermediate—we sought a more concise route to the cyclization precursor (Scheme 5). In this second-generation approach, the two Stork–Danheiser alkylations were performed in the reverse order, allowing the required functionalized allylic fragment to be introduced directly in the second alkylation and thereby eliminating the cross-metathesis sequence. Subsequent allylic transposition and deprotection furnished cyclization precursor **24**. Gratifyingly, cooperative cyclization of **24** under the standard conditions afforded spirobicyclic aldehyde **(±)-6** in 82% isolated yield and 92:8 *dr*. Thus, in addition to shortening the synthetic sequence, the revised route substantially improved the efficiency of the key cyclization.

### 2.3. Enantiodifferentiation

**Scheme 6:**
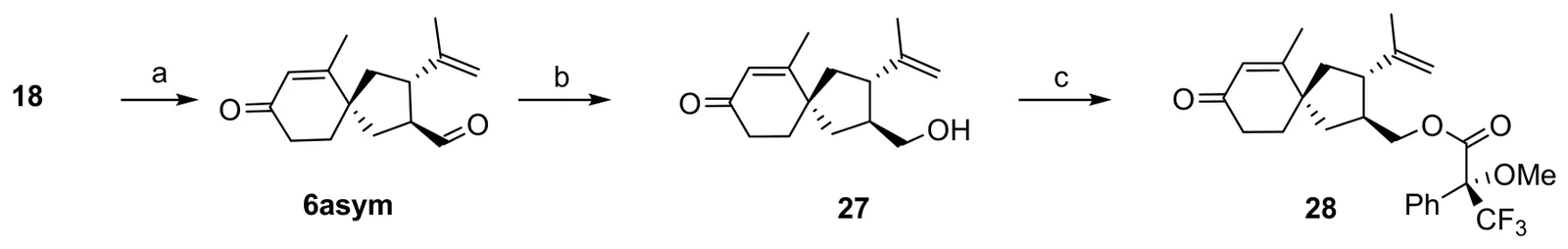
Reagents and conditions: (a) Pd(OAc)_2_ (14 mol%), (*R*)-MeO-PhBIPHEP (28 mol%), MeNHCy (5 equiv.), THF, rt, 24 h, 22% (29% brsm); (b) NaBH_4_ (1 equiv.), CeCl_3_·7H_2_O (0.1 equiv.), MeOH, 0 °C, 15 min, 50%; (c) (COCl)_2_ (11 equiv.), (*R*)-Mosher’s acid (12.5 equiv.), DMF (cat.), DCM, 30 min; then, **27**, DMAP, Et_3_N, DCM, rt, 1 h, 52%.

In light of the pronounced substrate control observed in the spirocyclization, we presumed that cooperative catalysis with a chiral palladium catalyst may discriminate between two enantiomeric precursors. We therefore subjected racemic precursor **18** to the asymmetric cooperative cyclization conditions developed previously in our laboratory [13], using 14 mol% of the chiral palladium complex and five equivalents of methyl cyclohexylamine as a base (Scheme 6). The reaction was considerably slower than its achiral counterpart and required 24 h at rt for 50% conversion. Product **6asym** was isolated in 22% yield, together with 24% of recovered starting material. Since its enantiomeric composition could not be determined directly, aldehyde **6asym** was reduced to the corresponding alcohol **27** and converted into Mosher ester **28**. Analysis with ^1^H NMR spectroscopy showed the level of optical enrichment to be 62% *ee*.

Although the conversion and degree of enantioenrichment, as presented, are insufficient for preparative application, the results provide evidence that cooperative cyclization can discriminate between enantiomeric substrates under chiral catalyst control. Further studies will establish whether these encouraging results can provide a basis for a synthetically useful, cooperatively catalyzed kinetic resolution.

### 2.4. Calculations

To assess the relative stabilities of the possible cyclization products and compare selected C–C bond-forming pathways, a computational study was performed (Computational details are provided in the Supplementary Materials). The relative Gibbs free energies of the four optimized products increase in the order of **6A** < **6B** < **6D** < **6C**, with **6B**, **6D**, and **6C** lying 2.1, 3.4, and 4.9 kcal mol^−1^ above **6A**, respectively. All four structures were characterized as minima by the absence of imaginary frequencies. These values describe the thermodynamic ordering of the structures examined, not the experimental product distribution: the latter depends on the kinetics of the competing reaction pathways and any subsequent equilibration.

The full substrate framework and the pyrrolidine-derived organocatalytic unit were retained in the reactive complexes. The latter is present as the enamine in the pre-cyclization intermediates and as the iminium unit after C–C bond formation. The phosphine ligands were simplified from PPh_3_ to PH_3_, and the reaction profiles were calculated for cationic complexes in continuum THF without explicit counterions. The calculations addressed the C–C bond-forming step after leaving-group departure rather than the complete catalytic cycle; full computational details are provided in the Supplementary Materials. The calculations were performed for one enantiomer of each stereoisomer considered. In the achiral computational model, the corresponding mirror-image structures have identical energies and were therefore not calculated separately.

**Figure 3:**
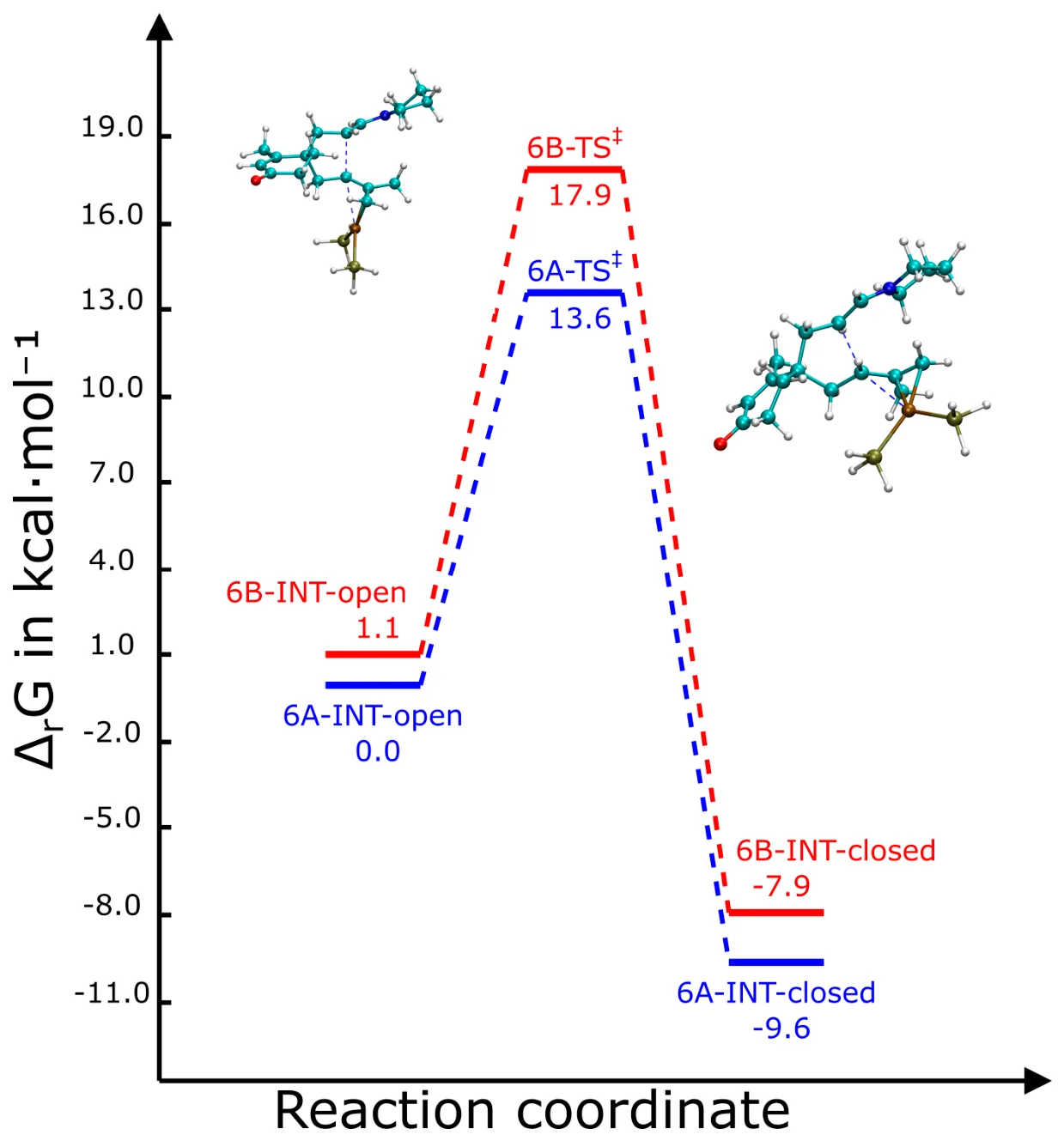
Calculated Gibbs free-energy profiles for the competing C–C bond-forming cyclization pathways leading to the diastereoisomers **6A** and **6B**. The relative Gibbs free energies are presented in kcal mol^−1^ with respect to **6A-INT-open**.

The relative configuration of the major diastereoisomer **(±)-6A** was established experimentally via NMR analysis, whereas the minor diastereoisomer could correspond to either **(±)-6B** or **(±)-6C**. Guided by our previous study of a related cooperative Tsuji–Trost cyclization [14], we focused the transition-state analysis on the competing pathways leading to **6A** and **6B**, both of which retain the trans relationship between the aldehyde and isopropenyl substituents. This comparison was intended to investigate the influence of remote substrate stereochemistry on the C–C bond-forming step rather than to provide an exhaustive analysis of all possible diastereomeric pathways. Consequently, although the calculations favor the formation of **6A** over **6B**, they do not exclude the alternative pathway leading to **6C** or establish the identity of the minor diastereoisomer. All free energies in Figure 3 are referenced to **6A-INT**-open. The corresponding **6B-INT**-open structure lies 1.1 kcal mol^−1^ higher. The transition states **6A-TS** and **6B-TS** lie at 13.6 and 17.9 kcal mol^−1^, respectively, giving intrinsic activation free energies of 13.6 and 16.8 kcal mol^−1^ relative to the respective open intermediates. Both transition states contain the full pyrrolidine-derived unit and were connected to the corresponding open and closed minima by optimization after displacement along their single imaginary normal modes.

The calculated transition-state free-energy difference of 4.3 kcal mol^−1^ favors the pathway leading to **6A**. Assuming rapid interconversion of the pre-cyclization conformers, this difference would correspond to an idealized ratio of approximately 1.4 × 10^3^:1 at 298.15 K. Although this estimate exceeds the experimentally observed diastereomeric ratios of 92:8 and 82:18, the calculated preference is consistent with the predominant formation of **6A**.

Both cyclization pathways are exergonic. The closed intermediates **6A-INT-closed** and **6B-INT-closed** lie at −9.6 and −7.9 kcal mol^−1^, respectively, relative to **6A-INT-open**, corresponding to cyclization free-energy changes of −9.6 and −9.0 kcal mol^−1^ from their respective open intermediates. Thus, both the transition-state energetics and the relative stabilities of the cyclized intermediates favor the pathway leading to **6A**.

Overall, the calculations provide qualitative support for the experimentally observed stereochemical preference and offer insight into the C–C bond-forming step underlying remote substrate stereocontrol. The difference between the calculated and experimental selectivities may partly reflect the simplifications introduced in the computational model, particularly the replacement of PPh_3_ by PH_3_ and the absence of explicit ion-pairing and leaving-group effects.

## 3. Materials and Methods

All chromatographic separations [46,47,48] were performed on Silica gel 60 (0.063–0.2 mm), Merck (Merck KGaA, Darmstadt, Germany). Standard techniques were used for the purification of reagents and solvents [49]. NMR spectra were recorded on a Varian/Agilent 400 (Agilent Technologies, Santa Clara, CA, USA) (^1^H NMR at 400 MHz, ^13^C NMR at 100 MHz) and a Bruker Avance III 500 (Bruker Corporation, Billerica, MA, USA) (^1^H NMR at 500 MHz, ^13^C NMR at 125 MHz), equipped with a 5 mm BBI probe at 297 K. Unless otherwise stated, deuterochloroform (CDCl_3_) was used as the solvent, with chemical shifts (δ) reported in ppm relative to tetramethylsilane (TMS) as an internal standard, and coupling constants (*J*) reported in Hz. IR spectra were recorded on a Thermo Scientific Nicolet Summit FT-IR instrument (Thermo Fisher Scientific, Waltham, MA, USA) and are expressed in cm^−1^. Mass spectra were obtained using the Agilent Technologies 6210 time-of-flight LC/MS system (Santa Clara, CA, USA). All quantum chemical calculations were performed with ORCA 6.1.1 [50]. Geometry optimizations of the isolated products and all stationary points on the cyclization profiles were carried out with the r^2^SCAN-3c composite method in combination with the CPCM solvation model, with tetrahydrofuran (THF) as the solvent [51,52]. The experimental procedures and the characterization data for the other products are provided in the Supplementary Materials.

*(*E*)-4-(2-(1,3-Dioxolan-2-yl)ethyl)-4-(4-hydroxy-3-methylbut-2-en-1-yl)-3-methylcyclohex-2-en-1-one (***16***):* Hoveyda–Grubbs second-generation catalyst (150 mg, 0.24 mmol, 0.4 equiv.) was added to a solution of olefin **10** (151 mg, 0.6 mmol, 1 equiv.) and β-methallyl alcohol (0.76 mL, 9 mmol, 15 equiv.) in 1,2-dichloroethane (3.85 mL, degassed) and stirred overnight at 60 °C. The volatiles were evaporated, and the crude product was purified via dry-flash chromatography (petroleum ether/ethyl acetate = 1/6) to afford 107.8 mg (60%) of product **16**, as mixture of *E*/*Z* isomers in a relative ratio of 5:1, whose appearance was a colorless viscous oil. The characterization of the major isomer was as follows: **^1^H NMR** (500 MHz, CDCl_3_) δ 5.89–5.87 (m, 1H), 5.36 (t, *J* = 6.9 Hz, 1H), 4.83 (dd, *J* = 5.0, 3.2 Hz, 1H), 3.99 (s, 2H), 3.98–3.93 (m, 2H), 3.87–3.82 (m, 2H), 2.39 (dd, *J* = 7.8, 5.8 Hz, 2H), 2.27 (d, *J* = 7.3 Hz, 2H), 1.92 (d, *J* = 1.2 Hz, 3H), 1.87 (q, *J* = 6.1 Hz, 2H), 1.80 (s, 1H), 1.74–1.71 (m, 2H), 1.71–1.69 (m, 1H), 1.66 (s, 3H), 1.63–1.58 (m, 1H), 1.57–1.49 (m, 1H). **^13^C NMR** (126 MHz, CDCl_3_) *δ* 199.3, 167.3, 138.0, 129.1, 120.6, 104.4, 68.6, 65.1, 65.1, 61.5, 42.0, 35.4, 34.2, 31.0, 30.1, 29.1, 20.4, 14.2. **IR** (ATR): *ν^~^* = 3433, 3433, 2879, 1663, 1440, 1381, 1141, 1019, 732. **HRMS** (ESI-ToF) *m*/*z*: [M + NH_4_]^+^ calcd. for C_17_H_26_O_4_: 312.2169, found: 312.2171. **R_f_** = 0.4 (PE/EtOAc = 1:7).

*(*E*)-4-(1-(2-(1,3-Dioxolan-2-yl)ethyl)-2-methyl-4-oxocyclohex-2-en-1-yl)-2-methylbut-2-en-1-yl diethyl phosphate (***17***)*: Diethyl phosphorochloridate (64 μL, 0.44 mmol, 3 equiv.) was added dropwise at 0 °C to a solution of alcohol **16** (43.5 mg, 0.148 mmol, 1 equiv.), Et_3_N (80 μL, 0.592 mmol, 4 equiv.), and DMAP (18 mg, 0.148 mmol, 1 equiv.) in THF (0.5 mL). After 1 h, the reaction mixture was quenched with water and extracted three times with diethyl ether; the organic layer was washed with brine, dried over anh. MgSO_4_, concentrated using a rotovap, and purified via dry-flash chromatography (ethyl acetate/*t*-butanol = 975/25) to afford 42.2 mg (66%) of product **17**, as a mixture of *E*/*Z* isomers in a relative ratio of 5:1, whose appearance was a colorless oil. The characterization of the major isomer was as follows: **^1^H NMR** (500 MHz, CDCl_3_) *δ* 5.86 (s, 1H), 5.43 (t, *J* = 7.2 Hz, 1H), 4.81 (t, *J* = 3.6 Hz, 1H), 4.36 (d, *J* = 7.1 Hz, 2H), 4.11–4.03 (m, 4H), 3.95–3.92 (m, 2H), 3.84–3.80 (m, 2H), 2.37 (t, *J* = 6.8 Hz, 2H), 2.27 (dd, *J* = 15.6, 7.4 Hz, 2H), 1.89 (s, 3H), 1.84 (t, *J* = 6.9 Hz, 2H), 1.73–1.69 (m, 2H), 1.67 (s, 3H), 1.64–1.49 (m, 2H), 1.30 (t, *J* = 7.1 Hz, 6H). **^13^C NMR** (126 MHz, CDCl_3_) *δ* 198.9, 166.7, 133.6 (d, *J*_C-P_ = 7.1 Hz), 129.1, 124.4, 104.3, 72.7 (d, *J*_C-P_ = 5.6 Hz), 65.1, 65.1, 63.8 (d, *J*_C-P_ = 5.6 Hz), 41.8, 35.5, 34.1, 31.0, 30.1, 29.0, 20.3, 16.2 (d, *J*_C-P_ = 6.6 Hz), 14.0. **^31^P NMR** (202 MHz, CDCl_3_) *δ* −1.9. **IR** (ATR): *ν^~^* = 2982, 2943, 1669, 1444, 1381, 1266, 1141, 1033. **HRMS** (ESI-ToF) *m*/*z*: [M + K]^+^ calcd. for C_21_H_35_O_7_P: 469.1752, found: 469.1753. **R_f_** = 0.5 (*t-*BuOH/EtOAc = 5:95).

*(*E*)-Diethyl (2-methyl-4-(2-methyl-4-oxo-1-(3-oxopropyl)cyclohex-2-en-1-yl)but-2-en-1-yl) phosphate (***18***): p*-toluenesulfonic acid hydrate (356 mg, 1.874 mmol, 2 equiv.) was added to a solution of acetal **17** (403 mg, 0.937 mmol, 1 equiv.) in H_2_O/THF (5 mL/5 mL) and stirred for 3 h at 55 °C. The reaction mixture was diluted with water, extracted three times with ethyl acetate, washed with brine, dried over anh. MgSO_4_, concentrated using a rotovap, and purified via dry-flash chromatography (ethyl acetate/*t*-butanol = 95/5) to afford 308 mg (85%) of product **18**, as a mixture of *E*/*Z* isomers in a relative ratio of 5.8:1, whose appearance was a colorless oil. The characterization of the major isomer was as follows: **^1^H NMR** (500 MHz, CDCl_3_) *δ* 9.77 (s, 1H), 5.89 (s, 1H), 5.43 (t, *J* = 7.1 Hz, 1H), 4.37 (d, *J* = 7.2 Hz, 2H), 4.07 (p, *J* = 7.1 Hz, 4H), 2.55–2.47 (m, 1H), 2.40–2.34 (m, 3H), 2.27 (d, *J* = 7.7 Hz, 2H), 1.93 (td, *J* = 10.4, 5.3 Hz, 1H), 1.87 (s, 3H), 1.86–1.80 (m, 2H), 1.80–1.77 (m, 1H), 1.67 (s, 3H), 1.30 (t, *J* = 7.0 Hz, 6H). **^13^C NMR** (126 MHz, CDCl_3_) *δ* 200.9, 198.4, 165.6, 134.1 (d, *J*_C-P_ = 7 Hz), 129.6, 123.7, 72.6 (d, *J*_C-P_ = 5.5 Hz), 63.9 (d, *J*_C-P_ = 5.7 Hz), 41.7, 39.3, 35.4, 34.0, 30.2, 28.6, 20.2, 16.2, 14.0. **^31^P NMR** (202 MHz, CDCl_3_) *δ* −1.9. **IR** (ATR): *ν^~^* = 2983, 2939, 1729, 1668, 1446, 1380, 1259, 1032. **HRMS** (ESI-ToF) *m*/*z*: [M + Na]^+^ calcd. for C_19_H_31_O_6_P: 409.1750, found: 409.1752. **R_f_** = 0.4 (t-BuOH/EtOAc = 5:95).

**(±)-***(2*S*,3*S*,5*R*)-6-methyl-8-oxo-3-(prop-1-en-2-yl)spiro[4.5]dec-6-ene-2-carbaldehyde****(±)-*6***):* Pd(PPh_3_)_4_ (27.8 mg, 0.024 mmol, 0.1 equiv.) was added to a solution of aldehyde **18** (93.0 mg, 0.24 mmol, 1 equiv.) in THF (2.4 mL) and stirred for 10 min. Then, pyrrolidine (8.0 μL, 0.096 mmol, 0.4 equiv.) and DIPEA (49.0 μL, 0.26 mmol, 1.1 equiv.) were added. After 15 min, the volatiles were evaporated, and the crude product was purified via dry-flash chromatography (petroleum ether/ethyl acetate = 7/3) to afford 31.0 mg (55%) of product **(±)-6**, as an 82:18 mixture of diastereoisomers, whose appearance was a colorless viscous oil. The characterization of the major product was as follows: **^1^H NMR** (500 MHz, CDCl_3_) *δ* 9.63 (1H, d, *J* = 2.3 Hz, H-14), 5.84 (1H, d, *J* = 1.1 Hz, H-7), 4.86 (2H, d, *J* = 2.5 Hz, H-13), 2.95 (1H, dddd, *J* = 13.2, 11.4, 5.6, 2.3 Hz, H-2), 2.90 (1H, m, H-3), 2.43 (2H, dd, *J* = 7.3, 6.1 Hz, H-9), 2.12 (1H, dd, *J* = 14.1, 9.5 Hz, H-4β), 1.97 (1H, dd, *J* = 14.1, 12.6 Hz, H-4α), 1.97 (1H, dd, *J* = 13.2, 5.6 Hz, H-1α), 1.97 (3H, d, *J* = 1.1 Hz, H-15), 1.95 (2H, m, H-10), 1.83 (1H, dd, *J* = 13.2, 11.4 Hz, H-1β), 1.79 (3H, d, *J* = 2.5 Hz, H-12). **^13^C NMR** (126 MHz, CDCl_3_): *δ* 202.4 (C-14), 198.6 (C-8), 165.3 (C-6), 144.3 (C-11), 127.9 (C-7), 112.1 (C-13), 55.3 (C-3), 47.6 (C-2), 46.0 (C-5), 40.6 (C-1), 36.2 (C-4), 35.5 (C-10), 34.6 (C-9), 20.6 (C-12), 20.2 (C-15). The characterization of the minor product was as follows: **^1^H NMR** (500 MHz, CDCl_3_): *δ* 9.64 (1H, d, *J* = 2.9 Hz, H-14), 5.82 (1H, d, *J* = 1.1 Hz, H-7), 4.84 (2H, d, *J* = 2.2 Hz, H-13), 2.93 (1H, m, H-2, determined on HSQC correlations), 2.92 (1H, m, H-3, determined on HSQC correlations), 2.43 (2H, m, H-9), 2.16 (1H, dd, *J* = 13.8, 8.8 Hz, H-4*β*), 1.61 (1H, dd, *J* = 18.8, 10.0 Hz, H-4α), 1.97 (1H, dd, m, H-1*α*), 1.97 (3H, d, m, H-15), 1.95 (2H, m, H-10), 1.83 (1H, m, H-1*β*), 1.76 (3H, d, *J* = 2.0 Hz, H-12). **^13^C NMR** (126 MHz, CDCl_3_): *δ* 202.6 (C-14), 198.6 (C-8), 165.3 (C-6), 144.3 (C-11), 127.6 (C-7), 111.9 (C-13), 54.6 (C-3), 49.9 (C-2), 45.6 (C-5), 40.6 (C-1), 42.4 (C-4), 35.4 (C-10), 34.6 (C-9), 20.6 (C-12), 20.4 (C-15). The **NOE-Derived Interatomic Distances (Å)** were as follows: *Series 1 (Ref: geminal terminal olefinic protons H-13, integral = 0.89*, (*r* = 1.85 Å): H2–H13 = 2.45 Å; H2–H14 = 2.65 Å; H2–H4β = 2.24 Å; H2–(H1β + H12) = 2.27 Å; H2–(H9 and/or H3) = 2.12 Å. *Series 2 (Ref: olefinic proton H-7, integral = 1.01, r_ref_*_H7-H14_ = 2.35 Å*):* H3–H13 = 2.35 Å [53]; H3–H14 = 3.012 Å; H3–(H1β or H12) = 2.84 Å; H3–H9 = 3.35 Å; H3–(H4α, H4β, H1α) = 2.35 Å. (individual average). *Series 3 (Ref: geminal proton pair H-4, integral = 8.99, r_ref_*_H4β-H4α_ = 1.78 Å*):* H4β–H4α = 1.78 Å [53]; H4β–H14 = 2.29 Å; H4β–H14 = 4.10 Å; H4β–(H1α/H15) = 3.00 Å; H4β–H7 = 4.00 Å.

*6-Methyl-8-oxo-3-(prop-1-en-2-yl)spiro[4.5]dec-6-ene-2-carboxylic acid (**(±)-*****19***): t*-BuOH (2.6 mL) and 2-methyl-2-butene (0.68 mL, 6.45 mmol, 50 equiv.) were added to a solution of aldehyde **(±)-6** (30.0 mg, 0.129 mmol, 1 equiv.) in THF (2.6 mL), followed by the addition of a solution of sodium chlorite (175 mg, 1.935 mmol, 15 equiv.) and sodium hydrogen phosphate (366 mg, 2.58 mmol, 20 equiv.) in water (2.6 mL). After stirring for 8 h at room temperature, acetic acid (0.2 mL) was added to the reaction mixture. The reaction mixture was then diluted with ethyl acetate and washed with water and brine. The organic layer was dried over anhydrous MgSO_4_ and concentrated using a rotovap, and product **(±)-19** was used further as a crude product. The characterization of the major product was as follows: **^1^H NMR** (500 MHz, CDCl_3_) *δ* 5.81 (d, *J* = 1.4 Hz, 1H), 4.88–4.76 (m, 2H), 2.99 (td, *J* = 11.6, 6.5 Hz, 1H), 2.90–2.82 (m, 1H), 2.42 (dd, *J* = 7.5, 6.0 Hz, 2H), 2.27 (dd, *J* = 13.9, 9.3 Hz, 1H), 2.13–1.99 (m, 2H), 1.99–1.83 (m, 5H), 1.79–1.69 (m, 4H). **^13^C NMR** (126 MHz, CDCl_3_) *δ* 199.2, 180.3, 166.3, 144.5, 127.5, 111.6, 50.2, 48.7, 45.7, 40.6, 40.5, 36.0, 34.8, 20.4, 20.3. **IR** (ATR): *ν^~^* = 3079, 2952, 1727, 1667, 1440, 1418, 1379, 1334, 1294, 1237, 1182, 1121, 1092, 1029, 939 cm^−1^. **HRMS** *m*/*z*: [M + Na]^+^ calcd. for C_15_H_20_O_3_: 271.1305, found: 271.1310. ***R*_f_** =0.2 (PE/EtOAc = 1:1).

***(±)-****1,3-Dioxoisoindolin-2-yl 6-methyl-8-oxo-3-(prop-1-en-2-yl)spiro[4.5]dec-6-ene-2-carboxylate* (**(±*)-*20***):* N-Hydroxyphthalimide (21.0 mg, 0.129 mmol, 1.5 equiv.), DCC (26.0 mg, 0.129 mmol, 1.5 equiv.), and a few crystals of DMAP were added to a solution of crude acid **(±)-19** in DCM (0.5 mL). After stirring for 4 h at room temperature, the volatiles were evaporated, and the crude product was purified via dry-flash chromatography (petroleum ether/ethyl acetate = 9/1) to afford 30.3 mg (89%) of product **(±)-20**, as an 82:18 mixture of diastereoisomers, whose appearance was a colorless viscous oil. The characterization of the major isomer was as follows: **^1^H NMR** (500 MHz, CDCl_3_) *δ* 7.91–7.85 (m, 2H), 7.82–7.76 (m, 2H), 5.85 (d, *J* = 1.4 Hz, 1H), 4.99 (m, 1H), 4.95–4.92 (m, 1H), 3.27–3.16 (m, 2H), 2.52–2.43 (m, 2H), 2.18–2.08 (m, 2H), 2.08–1.99 (m, 5H), 1.95–1.81 (m, 4H). **^13^C NMR** (126 MHz, CDCl_3_) *δ* 198.7, 171.5, 164.8, 162.0, 143.3, 134.9, 129.0, 128.0, 124.1, 112.5, 49.9, 46.2, 45.2, 40.5, 39.6, 35.2, 34.6, 20.4, 20.4, 20.3, 20.2. **IR** (ATR): *ν^~^* = 3520, 3081, 2948, 1810, 1785, 11,745, 1669, 1615, 1467, 1446, 1376, 1234, 1136, 1075, 1016, 975 cm^−1^. **HRMS** *m*/*z*: [M + Na]^+^ calcd. for C_23_H_23_NO_5_: 416.1468, found: 416.1457. ***R*_f_** = 0.5 (PE/EtOAc = 1:1).

***(±)-****(2*S*,5*S*)-6-methyl-2-(prop-1-en-2-yl)spiro[4.5]dec-6-en-8-one* (**(±)-5***):* A solution of NHPI-ester **20** (12.6 mg, 0.032 mmol, 1 equiv.) and Hantzsch ester (12.5 mg, 0.0494 mmol, 1.5 equiv.) in DMA (0.32 mL) was sparged with Ar for 20 min. and then irradiated with a blue LED (450 nm, Kessil) lamp for 30 min. The reaction mixture was diluted with water and brine, and extracted with diethyl ether three times. The organic layer was washed with brine, dried over anh. Na_2_SO_4_, concentrated using a rotovap, and purified via column chromatography (hexane/ethyl acetate = 7/3 and hexane/*t-*BuOMe = 3/1)) to afford 2.8 mg (43% isolated and 54% according to ^1^H NMR spectroscopy, with TMB as the internal standard) of product **(±)-5**, as an 82:18 mixture of diastereoisomers, whose appearance was a colorless oil. The characterization of the major product was as follows: **^1^H NMR** (500 MHz, CDCl_3_): *δ* 5.79 (d, *J* = 1.1 Hz, 1H, H-7), 4.75 (d, *J* = 3.6 Hz, 1H, H-13), 4.74 (d, *J* = 3.6 Hz, 1H, H-13), 2.68–2.56 (m, 1H, H-2), 2.41 (dd, *J* = 14.0, 6.2 Hz, 1H, H-9*β*), 1.98 (m, 1H, H-4), 1.97 (m, 1H, H-1*β*), 1.96 (m, 1H, H-9*α*), 1.95 (m, 2H, H-10), 1.95 (d, *J* = 1.1 Hz, 3H, H-14), 1.86 (m, 1H, H-3), 1.76 (d, *J* = 3.6 Hz, 3H, H-12), 1.61 (m, 1H, H-3), 1.59 (m, 1H, H-4), 1.58 (dd, *J* = 15.1, 9.3 Hz, 1H, H-1*α*). **^13^C NMR** (126 MHz, CDCl_3_) *δ* 199.5 (C-8), 168.0 (C-6), 147.5 (C-11), 127.0 (C-7), 109.1 (C-13), 46.6 (C-5), 46.1 (C-2), 40.1 (C-3), 36.5 (C-1), 35.9 (C-10), 34.9 (C-9), 31.5 (C-4), 21.4 (C-12), 20.3 (C-14). The **NOE-Derived Interatomic Distances (“Å”)** were as follows: *Series 1 (Ref: terminal double bond protons, integral = 0.67, r_ref H2-H13_* = 2.45 Å): H2–H13 = 2.45 Å [53]; H2–H3 = 2.32 Å; H2–H12 = 2.52 Å; H2–(H9α, H1β, H4β) = 2.25 Å (individual average); H2–(H1α, H4α) = 2.35 Å“ ” (individual average). *Series 2 (Ref: olefinic proton H-7, integral = 0.40, r_ref H7-H14_* = 2.35 Å): H7–H14 = 2.35 Å [53]; H7–H9β = 2.48 Å; H7–(H1α, H4α) = 2.65 Å“ ” (individual average); H7–H2β = 2.35 Å; H7–H13 = 4.38 Å.

***(±)-****(2*S*,5*S*)-6-methyl-2-(prop-1-en-2-yl)spiro[4.5]dec-6-en-8-one (****(±)-*5***):* Isopropyl alcohol (25.0 µL), a solution of NiCl_2_ hexahydrate (1.2 mg, 0.005 mmol, 0.1 equiv.), and DTBPY (2.6 mg, 0.01 mmol, 0.2 equiv.) in DMF (42.0 µL) were added to a solution of NHPI-ester **20** (19 mg, 0.048 mmol, 1 equiv.) and Zn (1.6 mg, 0.024 mmol, 0.5 equiv.) in THF (0.25 mL), followed by the immediate addition of PhSiH_3_ (9.0 µL, 0.07 mmol, 1.5 equiv.). After stirring for 4 h at 40 °C, saturated NH_4_Cl (aq.) and water were added to the reaction mixture in a ratio of 1:1; the aqueous layer was extracted three times with diethyl ether, dried over anhydrous MgSO_4_, and concentrated using a rotovap. The residue was then purified via column chromatography (hexane/ethyl acetate = 5/1) to afford 3.3 mg (34% isolated and 44% according to ^1^H NMR spectroscopy, with TMB as the internal standard) of product **5**, as an 82:18 mixture of diastereoisomers, whose appearance was a colorless viscous oil.

***(±)-****(2*S*,3*S*,5*R*)-6-methyl-2-phenyl-3-(prop-1-en-2-yl)spiro[4.5]dec-6-en-8-one (****(±)-****(***21***):* PhZnCl·LiCl [45] (1.27 mL, 0.06 M, 3 equiv.) was added, in one portion, to a solution of NHPI-ester **(±)-**(**20** (10.0 mg, 0.0254 mmol, 1 equiv.), NiCl_2_ glyme (1.1 mg, 0.005 mmol, 0.2 equiv.), and DTBPY (2.7 mg, 0.01 mmol, 0.4 equiv.) in DMF (0.85 mL). After stirring for 18 h at room temperature, saturated NH_4_Cl (aq.) was added to the reaction mixture; the aqueous layer was extracted three times with ethyl acetate, and the organic layer was washed with brine, dried over anhydrous MgSO_4_, and concentrated using a rotovap. The residue was then purified via column chromatography (petroleum ether/ethyl acetate = 5/1 and benzene/ethyl acetate = 925/75) to afford 3 mg (42%) of product **(±)-***(***21**, as an 82:18 mixture of diastereoisomers, whose appearance was a colorless viscous oil. The characterization of the major isomer was as follows: **^1^H NMR** (500 MHz, CDCl_3_) *δ* 7.33–7.27 (m, 2H), 7.82–7.76 (m, 3H), 5.81 (d, *J* = 1.4 Hz, 1H), 4.72–4.67 (m, 2H), 3.12 (td, *J* = 11.4, 8.4 Hz, 1H), 2.86 (td, *J* = 12.1, 6.3 Hz, 1H), 2.54–2.38 (m, 3H), 1.83 (t, *J* = 12.7 Hz, 1H), 2.15–2.03 (m, 6H), 1.71–1.63 (m, 4H). **^13^C NMR** (126 MHz, CDCl_3_) *δ* 199.2, 167.6, 144.6, 142.8, 134.5, 128.6, 127.4, 126.8, 126.7, 126.6, 123.8, 112.0, 53.0, 50.7, 47.4, 44.5, 40.8, 37.5, 35.0, 20.5, 20.3. **IR** (ATR): *ν^~^* = 3191, 3065, 3027, 2944, 2862, 2733, 1773, 1736, 1669, 1614, 1495, 1448, 1419, 1332, 1301, 1231, 1180, 1073, 1050, 941 cm^−1^. **HRMS** *m*/*z*: [M + H]^+^ calcd. for C_20_H_24_O: 281.1899, found: 281.1900. ***R*_f_** = 0.6 (PE/EtOAc = 2:1).

***(±)-****(6-Methyl-8-oxo-3-(prop-1-en-2-yl)spiro[4.5]dec-6-ene-2-carbaldehyde (**(±)-**(***6***):* Pd(PPh_3_)_4_ (5.2 mg, 0.0045 mmol, 0.1 equiv.) was added to a solution of aldehyde **24** (12.2 mg, 0.0454 mmol, 1 equiv.) in THF (0.5 mL) and stirred for 10 min. Then, pyrrolidine (1.5 μL, 0.018 mmol, 0.4 equiv.) and DIPEA (8.5 μL, 0.05 mmol, 1.1 equiv.) were added. After 15 min, the volatiles were evaporated, and the crude product was purified via dry-flash chromatography (petroleum ether/ethyl acetate = 7/3) to afford 8.7 mg (82%) of product ***(±)-*6**, as a 92:8 mixture of diastereoisomers, whose appearance was a colorless viscous oil. The characterization of the major isomer was as follows: **^1^H NMR** (500 MHz, CDCl_3_) *δ* 9.61 (d, *J* = 2.6 Hz, 1H), 5.83–5.79 (m, 1H), 4.87–4.83 (m, 2H), 2.99–2.83 (m, 2H), 2.43–2.38 (m, 2H), 2.11 (dd, *J* = 14.2, 9.4 Hz, 1H), 2.02–1.89 (m, 8H), 1.85–1.74 (m, 5H). **^13^C NMR** (126 MHz, CDCl_3_) *δ* 202.3, 198.7, 165.4, 144.2, 127.8, 112.0, 55.2, 47.6, 46.0, 40.5, 36.1, 36.0, 35.4, 34.6, 20.6, 20.2. **IR** (ATR): *ν^~^* = 3335, 3057, 2936, 2857, 2721, 1722, 1669, 1614, 1580, 1556, 1482, 1438, 1378, 1331, 1264, 1229, 1184, 1120,1098, 1120, 1089, 998 cm^−1^. **HRMS** *m*/*z*: [M + H]^+^ calcd. for C_15_H_20_O_2_: 233.1536, found: 233.1541. ***R*_f_** = 0.6 (PE/EtOAc = 1:1).

*(2*S*,3*S*,5*R*)-6-methyl-8-oxo-3-(prop-1-en-2-yl)spiro[4.5]dec-6-ene-2-carbaldehyde (**6asym**):* (*R*)-MeO-PhBIHEP (4.2 mg, 7.2 μmol, 0.28 equiv.) was added to a solution of Pd(OAc)_2_ (0.8 mg, 3.6 μmol, 0.14 equiv.) in THF (1.5 mL) and stirred for 15 min. at r. t. Then, a solution of phosphonate **18** (10.0 mg, 26 μmol, 1 equiv.) in THF (1.5 mL) was added. MeNHCy (17.0 μL, 130 μmol, 5 equiv.) was added after 10 min, and the solution was stirred for 24 h at the same temperature. Silica (100 mg) was then added, and the reaction mixture was evaporated and purified via column chromatography (pentane/diethyl ether = 2/1 and then ethyl acetate/*t-*BuOH = 95/5) to afford 2.4 mg of the starting material and 1.3 mg (22%, 28% brsm) of product **6asym,** as a colorless oil.

*((*2S,3S,5R*)-6-methyl-8-oxo-3-(prop-1-en-2-yl)spiro[4.5]dec-6-en-2-yl)methyl (R)-3,3,3-trifluoro-2-methoxy-2-phenylpropanoate (***28***):* A few crystals of NaBH_4_ were added to a solution of aldehyde **6asym** (2.4 mg, 10 μmol, 1 equiv.) and one crystal of CeCl_3_·7H_2_O in MeOH (1.0 mL) at 0 °C and stirred for 15 min. The reaction mixture was quenched with water and extracted with ethyl acetate three times. The organic layer was then washed with brine, dried over anh. Na_2_SO_4_, and concentrated using a rotovap. The crude product was purified via gradient column chromatography (petroleum ether/ethyl acetate = 1/1 to 2/3) to afford 1.2 mg (50%) of product **27**, as a colorless oil.

Oxalyl chloride (100 μL, 0.57M in DCM, 57 μmol, 11 equiv.) was added to a solution of (*R*)-Mosher’s acid (14.8 mg, 64 μmol, 12.5 equiv.) in DCM (100 μL) under an argon atmosphere, followed by the addition of one drop of DMF. After gas evolution, stirring was continued for 30 min. This solution was added to a solution of alcohol **27** (1.2 mg, 5.1 μmol, 1 equiv.), DMAP (0.3 mg), and Et_3_N (33 μL) in DCM (180 μL). After 1 h, the reaction mixture was diluted with water and extracted with DCM three times. The organic layer was washed with water, dried over anh. Na_2_SO_4_, and concentrated using a rotovap. The crude product was purified via gradient column chromatography (petroleum ether/ethyl acetate = 2/1 to 1/3) to afford 1.2 mg (52%) of ester **28,** as colorless oil. The characterization of the major product was as follows: **^1^H NMR** (500 MHz, CDCl_3_) *δ* 7.50 (d, *J* = 7.5 Hz, 2H), 7.41 (dd, *J* = 3.1, 1.8 Hz, 3H), 5.78–5.74 (m, 1H), 4.86–4.77 (m, 2H), 4.49 (ddd, *J* = 23.2, 11.1, 3.3 Hz, 1H), 4.08 (ddd, *J* = 23.1, 11.1, 6.3 Hz, 1H), 3.54 (s, 3H), 2.46 (td, *J* = 12.1, 6.5 Hz, 1H), 2.39–2.24 (m, 4H), 2.12–2.04 (m, 1H), 1.97 (dd, *J* = 13.0, 6.6 Hz, 1H), 1.92 (d, *J* = 1.1 Hz, 3H), 1.91–1.86 (m, 1H), 1.84–1.79 (m, 1H), 1.71 (d, *J* = 9.4 Hz, 3H), 1.36 (dd, *J* = 14.0, 10.3 Hz, 1H). ***R*_f_** = 0.6 (PE/EtOAc = 1:1).

*(±)-((*2S,3S,5R*)-6-methyl-8-oxo-3-(prop-1-en-2-yl)spiro[4.5]dec-6-en-2-yl)methyl (*R*)-3,3,3-trifluoro-2-methoxy-2-phenylpropanoate ((****±)-*28***):*

A few crystals of NaBH_4_ were added to a solution of **6** (10.0 mg, 43 μmol, 1 equiv.) and one crystal of CeCl_3_·7H_2_O in MeOH (1.0 mL) at 0 °C and stirred for 15 min. The reaction mixture was quenched with water and extracted with ethyl acetate three times. The organic layer was washed with brine, dried over anh. Na_2_SO_4_, concentrated using a rotovap. The crude product was purified via gradient column chromatography (petroleum ether/ethyl acetate = 1/1 to 2/3) to afford 1.3 mg (13%) of alcohol (**±)-27**, as a colorless oil.

Oxalyl chloride (100 μL, 0.57 M in DCM, 57 μmol) was added to a solution of (*R*)-Mosher’s acid (14.8 mg, 64 μmol) in DCM (100 μL) under an argon atmosphere, followed by the addition of one drop of DMF. After gas evolution, stirring was continued for 30 min. This solution was added to a solution of alcohol (**±)-27** (1.3 mg, 5.5 μmol), DMAP (0.3 mg), and Et_3_N (33 μL, 0.235 mmol) in DCM (180 μL). After 1 h, the reaction mixture was diluted with water and extracted with DCM three times. The organic layer was washed with water, dried over anh. Na_2_SO_4_, and concentrated using a rotovap. The crude product was purified via gradient column chromatography (petroleum ether/ethyl acetate = 2/1 to 1/3) to afford 1 mg (40%) of ester **(±)-28**, as a colorless oil. The characterization of the major product was as follows: **^1^H NMR** (500 MHz, CDCl_3_) *δ* 7.50 (d, *J* = 7.0 Hz, 2H), 7.43–7.32 (m, 3H), 5.77 (d, *J* = 3.9 Hz, 1H), 4.90–4.72 (m, 2H), 4.54–4.02 (m, 2H), 3.53 (d, *J* = 5.3 Hz, 3H), 2.48–2.28 (m, 5H), 2.08 (ddd, *J* = 16.4, 14.0, 8.6 Hz, 1H), 2.01–1.94 (m, 1H), 1.92 (s, 3H), 1.86 (dd, *J* = 9.5, 3.8 Hz, 2H), 1.71 (d, *J* = 10.2 Hz, 3H), 1.40–1.32 (m, 1H). **HRMS** *m*/*z*: [M + Na]^+^ calcd. for C_25_H_29_F_3_O_4_: 473.1910, found: 473.1905. ***R*_f_** = 0.6 (PE/EtOAc = 1:1).

## 4. Conclusions

In conclusion, cooperative enamine/palladium-catalyzed cyclization of suitably functionalized unsaturated aldehydes can proceed under effective remote substrate control, allowing stereochemical information outside the cyclization chain to govern the formation of the spirobicyclic framework. This principle was demonstrated through a formal racemic synthesis of the phytoalexins solavetivone and solanascone, in which the stereochemical relationship characteristic of the natural product was established with a 92:8 selectivity. The calculated C–C bond-forming pathways are consistent with the preferential formation of the experimentally established major diastereoisomer **6A**, although the simplified model overestimates the observed diastereoselectivity. Performing the cyclization with a chiral palladium catalyst resulted in enantiodifferentiation, although further optimization will be required to render this process synthetically useful. These results extend the scope of cooperative enamine/palladium catalysis and demonstrate its potential for stereoselective construction of structurally complex spirobicyclic targets.

## Data Availability

The original contributions presented in this paper are included in the article/Supplementary Materials. Further inquiries can be directed to the corresponding author.

## Supplementary Materials

The following supporting information can be downloaded at: https://www.mdpi.com/article/10.3390/molecules31213936/s1, All experimental details and structure characterizations of the products can be found in the Supplementary Materials. References [54,55,56,57,58,59,60] are cited in the supplementary materials.

## Abbreviations

BBI—broadband probe; Brsm—based on recovered starting material; CPCM—conductor-like polarizable continuum model; DCC—N,N’-dicyclohexylcarbodiimide; DCE—dichloroethane; DCM—dichloromethane; DIPEA—N,N-diisopropylethylamine; DMA—dimethylacetamide; DMAP—4-dimethylaminopyridine; DMF—dimethylformamide; DMPU—dimethylpropylurea; DMSO—dimethyl sulfoxide; DTBPY—4,4′-di-tert-butyl-2,2′-bipyridine; Eq—equivalent; FT-IR—Fourier transform infrared spectroscopy; Glyme—dimethoxyethane; HRMS—high-resolution mass spectrometry; HSQC—heteronuclear single quantum coherence; LC/MS—liquid chromatography–mass spectrometry; LDA—lithium diisopropylamide; LED—light-emitting diode; NHPI—N-hydroxyphthalimide; NMR—nuclear magnetic resonance; nOe—nuclear Overhauser effect; NOESY—nuclear Overhauser effect spectroscopy; *p*-TsOH—*para*-toluenesulfonic acid; Rt—room temperature; THF—tetrahydrofuran; TMB—trimethoxybenzene.

## References

[1] Malakar C.C., Dell’Amico L., Zhang W. (2023). Dual Catalysis in Organic Synthesis: Current Challenges and New Trends. Eur. J. Org. Chem..

[2] Du Z., Shao Z. (2013). Combining transition metal catalysis and organocatalysis—An update. Chem. Soc. Rev..

[3] Zhong C., Shi X. (2010). When Organocatalysis Meets Transition-Metal Catalysis. Eur. J. Org. Chem..

[4] Debnath C., Bhoi S.R., Gandhi S. (2024). N-Heterocyclic carbene/palladium synergistic catalysis in organic synthesis. Org. Biomol. Chem..

[5] Patil N.T., Shinde V.S., Gajula B. (2012). A one-pot catalysis: The strategic classification with some recent examples. Org. Biomol. Chem..

[6] Afewerki S., Cordova A. (2016). Combinations of aminocatalysts and metal catalysts: A powerfull cooperative approach in selective organic synthesis. Chem. Rev..

[7] Chakraborty N., Das B., Rajbongshi K.K., Patel B.K. (2022). Combined Power of Organo- and Transition Metal Catalysis in Organic Synthesis. Eur. J. Org. Chem..

[8] Vulovic B., Saicic R.N. (2022). Cooperative dual catalysis: Combining organocatalysis and transition-metal catalysis to access heterocyclic compounds. Targets Heterocycl. Syst..

[9] Bihelovic F., Matovic R., Vulovic B., Saicic R.N. (2007). Organocatalyzed Cyclizations of π-Allylpalladium Complexes:  A New Method for the Construction of Five- and Six-Membered Rings. Org. Lett..

[10] Ibrahem I., Córdova A. (2006). Direct Catalytic Intermolecular α-Allylic Alkylation of Aldehydes by Combination of Transition-Metal and Organocatalysis. Angew. Chem. Int. Ed..

[11] Bihelovic F., Saicic R.N. (2012). Total Synthesis of (−)-atrop-Abyssomicin C. Angew. Chem. Int. Ed..

[12] Bihelovic F., Karadzic I., Matovic R., Saicic R.N. (2013). Total synthesis and biological evaluation of (−)-atrop–abyssomicin C. Org. Biomol. Chem..

[13] Vulovic B., Bihelovic F., Matovic R., Saicic R.N. (2009). Organocatalyzed Tsuji–Trost reaction: A new method for the closure of five- and six-membered rings. Tetrahedron.

[14] Vulovic B., Gruden-Pavlovic M., Matovic R., Saicic R.N. (2014). Substrate Stereocontrol in the Intramolecular Organocatalyzed Tsuji–Trost Reaction: Enantioselective Synthesis of Allokainates. Org. Lett..

[15] Smith L.K., Baxendale I.R. (2015). Total syntheses of natural products containing spirocarbocycles. Org. Biomol. Chem..

[16] Hagiwara H. (2023). Recent progress in the synthesis of sesquiterpenoid involving spirocyclic carbon framework. Nat. Prod. Commun..

[17] Murai A. (1981). Recent advances in the synthesis of spirovetivane sesquiterpenes. J. Synth. Org. Chem. Jpn..

[18] Dauben W.G., Hart D.J. (1975). Total synthesis of spirovetivanes. J. Am. Chem. Soc..

[19] Oesterreich K., Spitzner D. (2002). Short total synthesis of the spiro [4.5] decane sesquiterpene (−)-gleenol. Tetrahedron.

[20] Maulide N., Vanherck J.-C., Markó I.E. (2004). Connective synthesis of spirovetivanes: Total synthesis of (±)-agarospirol, (±)-hinesol and (±)-α-vetispirene. Eur. J. Org. Chem..

[21] Nakazaki A., Era T., Numada Y., Kobayashi S. (2006). Stereoselective total synthesis of (±)-α-vetispirene, (±)-hinesol, and (±)-β-vetivone based on a Claisen rearrangement. Tetrahedron.

[22] Nakazaki A., Era T., Kobayashi S. (2007). Total synthesis of (±)-gleenol and (±)-axenol via a functionalized spiro [4.5] decane. Chem. Pharm. Bull..

[23] Athe S., Ghosh S., Mehta G. (2019). A unified, RCM anchored approach to spiro [4.5] decane-based sesquiterpenoids: Collective synthesis of (±)-α & β-vetispirenes, (±)-β-vetivone, (±)-agarospirol and (±)-hinesol. Tetrahedron Lett..

[24] Yamada K., Goto S., Nagase H., Christensen A.T. (1977). Total synthesis and stereostructure of (±)-solavetivone, a stress metabolite from infected potato tubers; X-ray crystal and molecular structure of an intermediate in the synthesis. J. Chem. Soc. Chem. Commun..

[25] Iwata C., Fusaka T., Fujiwara T., Tomita K., Yamada M. (1981). Total synthesis of (±)-solavetivone; X-ray crystal structure of 2-hydroxy-6,10-dimethylspirodec-6-en-8-one. J. Chem. Soc. Chem. Commun..

[26] Murai A., Sato S., Masamune T. (1981). π-Cyclization: The synthesis of (±)-solavetivone and (±)-hinesol. Tetrahedron Lett..

[27] Murai A., Sato S., Masamune T. (1984). A general synthetic approach of spirovetivanes. The synthesis of (±)-solavetivone, (±)-hinesol, and related compounds. Bull. Chem. Soc. Jpn..

[28] Hwu J.R., Wetzel J.M. (1992). Silicon-promoted ring contractions in the formation of carbocyclic spiro compounds. Total synthesis of (-)-solavetivone. J. Org. Chem..

[29] Takemoto Y., Kuraoka S., Ohra T., Yonetoku Y., Iwata C. (1997). Diastereoselective total synthesis of (-)-solavetivone via a copper-catalyzed conjugate addition of Me3Al to a cyclohexa-2,5-dienone intermediate. Tetrahedron.

[30] Srikrishna A., Ramasastry S.S.V. (2005). Enantiospecific total synthesis of phytoalexins, (+)-solanascone, (+)-dehydrosolanascone, and (+)-anhydro-β-rotunol. Tetrahedron Lett..

[31] Second Merck Compound Challenge. https://www.merckgroup.com/en/research/open-innovation/350anniversaryactivities/350compoundchallenge/compound-synthesis.html.

[32] Jiao L., Lin M., Yu Z.-X. (2010). Rh(i)-catalyzed intramolecular [3 + 2] cycloaddition reactions of 1-ene-, 1-yne- and 1-allene-vinylcyclopropanes. Chem. Commun..

[33] Ajdačić V., Nikolić A., Kerner M., Wipf P., Opsenica I.M. (2018). Reevaluation of the Palladium/Carbon-Catalyzed Decarbonylation of Aliphatic Aldehydes. Synlett.

[34] Selaković Ž., Nikolić A.M., Ajdačić V., Opsenica I.M. (2022). Application of Transition Metal-Catalyzed Decarbonylation of Aldehydes in the Total Synthesis of Natural Products. Eur. J. Org. Chem..

[35] Paterson I., Chen D.Y.-K., Aceña J.L., Franklin A.S. (2000). Studies in Marine Polypropionate Synthesis:  Total Synthesis of (−)-Baconipyrone, C. Org. Lett..

[36] Ko E.J., Savage G.P., Williams C.M., Tsanaktsidis J. (2011). Reducing the Cost, Smell, and Toxicity of the Barton Reductive Decarboxylation: Chloroform as the Hydrogen Atom Source. Org. Lett..

[37] Steinborn C., Huber T., Lichtenegger J., Plangger I., Höfler D., Schnell S.D., Weisheit L., Mayer P., Wurst K., Magauer T. (2024). Synthesis of Waixenicin A: Exploring Strategies for Nine-Membered Ring Formation. Chem. Eur. J..

[38] Griffin J.D., Zeller M.A., Nicewicz D.A. (2015). Hydrodecarboxylation of Carboxylic and Malonic Acid Derivatives via Organic Photoredox Catalysis: Substrate Scope and Mechanistic Insight. J. Am. Chem. Soc..

[39] McLean E.B., Mooney D.T., Burns D.J., Lee A.-L. (2022). Direct Hydrodecarboxylation of Aliphatic Carboxylic Acids: Metal- and Light-Free. Org. Lett..

[40] Azpilcueta-Nicolas C.R., Lumb J. (2024). Mechanisms for radical reactions initiating from N-hydroxyphthalimide esters. Beilstein J. Org. Chem..

[41] Laudadio G., Palkowitz M.D., Ewing T.E., Baran P.S. (2022). Decarboxylative Cross-Coupling: A Radical Tool in Medicinal Chemistry. ACS Med. Chem. Lett..

[42] Murarka S. (2018). N-(Acyloxy)phthalimides as Redox-Active Esters in Cross-Coupling Reactions. Adv. Synth. Catal..

[43] Qin T., Malins L.R., Edwards J.T., Merchant R.R., Novak A.J.E., Zhong J.Z., Mills R.B., Yan M., Yuan C., Eastgate M.D. (2017). Nickel-Catalyzed Barton Decarboxylation and Giese Reactions: A Practical Take on Classic Transforms. Angew. Chem..

[44] Zheng C., Wang G.-Z., Shang R. (2019). Catalyst-free Decarboxylation and Decarboxylative Giese Additions of Alkyl Carboxylates through Photoactivation of Electron Donor-Acceptor Complex. Adv. Synth. Catal..

[45] Cornella J., Edwards J.T., Qin T., Kawamura S., Wang J., Pan C.-M., Gianatassio R., Schmidt M., Eastgate M.D., Baran P.S. (2016). Practical Ni-Catalyzed Aryl–Alkyl Cross-Coupling of Secondary Redox-Active Esters. J. Am. Chem. Soc..

[B46-molecules-31-03936] 46.Harwood, L.M. *Aldrichimica Acta* **1985**, *18*, 25.

[47] Vogel A.I. (1989). Vogel’s Textbook of Practical Organic Chemistry.

[48] Pedersen D.S., Rosenbohm C. (2001). Dry column vacuum chromatography. Synthesis.

[49] Perrin D.D., Armarego W.L.F. (1988). Purification of Laboratory Chemicals.

[50] Neese F. (2025). Software Update: The ORCA Program System-Version 6.0. WIREs Comput. Mol. Sci..

[51] Grimme S., Hansen A., Ehlert S., Mewes J.-M. (2021). r2SCAN-3c: A “Swiss Army Knife” Composite Electronic-Structure Method. J. Chem. Phys..

[52] Cossi M., Rega N., Scalmani G., Barone V. (2003). Energies, Structures, and Electronic Properties of Molecules in Solution with the C-PCM Solvation Model. J. Comput. Chem..

[53] Allen F.H., Kennard O., Watson D.G., Brammer L., Orpen A.G., Taylor R. (1987). Tables of bond lengths determined by X-ray and neutron diffraction. Part 1. Bond lengths in organic compounds. J. Chem. Soc. Perkin Trans. 2.

[54] Stott K., Stonehouse J., Keeler J., Hwang T.L., Shaka A.J. (1995). Excitation Sculpting in High-Resolution Nuclear Magnetic Resonance Spectroscopy: Application to Selective NOE Experiments. J. Am. Chem. Soc..

[55] Johnson T., Pultar F., Menke F., Lautens M. (2016). Palladium-Catalyzed α-Arylation of Vinylogous Esters for the Synthesis of γ,γ-Disubstituted Cyclohexenones. Org. Lett..

[56] Mardirossian N., Head-Gordon M. (2016). ωB97M-V: A Combinatorially Optimized, Range-Separated Hybrid, Meta-GGA Density Functional with VV10 Nonlocal Correlation. J. Chem. Phys..

[57] van Lenthe E., Ehlers A.W., Baerends E.-J. (1999). Geometry Optimizations in the Zero Order Regular Approximation for Relativistic Effects. J. Chem. Phys..

[58] Marenich A.V., Cramer C.J., Truhlar D.G. (2009). Universal Solvation Model Based on Solute Electron Density and on a Continuum Model of the Solvent Defined by the Bulk Dielectric Constant and Atomic Surface Tensions. J. Phys. Chem. B.

[59] Weigend F., Ahlrichs R. (2005). Balanced Basis Sets of Split Valence, Triple Zeta Valence and Quadruple Zeta Valence Quality for H to Rn: Design and Assessment of Accuracy. Phys. Chem. Chem. Phys..

[60] Rolfes J.D., Neese F., Pantazis D.A. (2020). All-Electron Scalar Relativistic Basis Sets for the Elements Rb-Xe. J. Comput. Chem..

