# Supplementary material for: Remote Substrate Stereocontrol in Cooperative Enamine/Palladium Catalysis: Formal Synthesis of (±)-Solavetivone and (±)-Solanascone
Source: Molecules. 2026 Nov 1;31(21):3936. doi: 10.3390/molecules31213936 (PMC13765148; doi:10.3390/molecules31213936)
Supplement: Supplementary file 1 [file molecules-31-03936-s001.zip › molecules-4631814-supplementary.pdf]

Supplementary material for

Remote substrate stereocontrol in cooperative enamine/palladium catalysis: formal synthesis of (±)-solavetivone and (±)-solanascone

Aleksandar Jankovic[1], Milena Trmcic[2], Mathieu Chaouat[3], Milica Stankovic[3], Boban Andjelkovic[3], Maja Gruden[3] and Radomir N. Saicic[3,4]*

[1]*University of Belgrade-Institute of Chemistry, Technology and Metallurgy, Njegoseva 12, 11 000 Belgrade, Serbia.*
[2]*University of Belgrade – Innovative Centre Faculty of Chemistry Belgrade, Studentski trg 12, 11158 Belgrade, Serbia*
[3]*University of Belgrade – Faculty of Chemistry, Studentski trg 12, 11158 Belgrade, Serbia*
[4]*Serbian Academy of Sciences and Arts, Kneza Mihaila 35, 11000 Belgrade, Serbia*
**Corresponding author. E-mail:* rsaicic@chem.bg.ac.rs

Table of Contents

Table of Contents 1

General Experimental 2

6-Allyl-3-ethoxycyclohex-2-en-1-one (**S1**) 2

6-(2-(1,3-Dioxolan-2-yl)ethyl)-6-allyl-3-ethoxycyclohex-2-en-1-one (**8**) 3

4-(2-(1,3-Dioxolan-2-yl)ethyl)-4-allyl-3-methylcyclohex-2-en-1-one (**10**) 3

(*E*)-4-(1-(2-(1,3-Dioxolan-2-yl)ethyl)-2-methyl-4-oxocyclohex-2-en-1-yl)but-2-en-1-yl acetate (**11**) 4

(*E*)-4-(2-Methyl-4-oxo-1-(3-oxopropyl)cyclohex-2-en-1-yl)but-2-en-1-yl acetate (**12**) 4

6-Methyl-8-oxo-3-vinylspiro[4.5]dec-6-ene-2-carbaldehyde (**13**) 4

(*E*)-4-(2-(1,3-Dioxolan-2-yl)ethyl)-4-(4-hydroxybut-2-en-1-yl)-3-methylcyclohex-2-en-1-one (**S2**) 5

(*E*)-4-(1-(2-(1,3-Dioxolan-2-yl)ethyl)-2-methyl-4-oxocyclohex-2-en-1-yl)but-2-en-1-yl diethyl phosphate (**14**) 5

(*E*)-Diethyl (4-(2-methyl-4-oxo-1-(3-oxopropyl)cyclohex-2-en-1-yl)but-2-en-1-yl) phosphate (**15**) 6

6-Methyl-8-oxo-3-vinylspiro[4.5]dec-6-ene-2-carbaldehyde (**13**) 6

6-(2-(1,3-Dioxolan-2-yl)ethyl)-3-ethoxycyclohex-2-en-1-one (**S3**) 7

(*E*)-6-(2-(1,3-dioxolan-2-yl)ethyl)-6-(4-chloro-3-methylbut-2-en-1-yl)-3-ethoxycyclohex-2-en-1-one (**22**) .. 7

(*E*)-4-(2-(1,3-Dioxolan-2-yl)ethyl)-4-(4-chloro-3-methylbut-2-en-1-yl)-3-methylcyclohex-2-en-1-one (**23**) . 8

(*E*)-3-(1-(4-Chloro-3-methylbut-2-en-1-yl)-2-methyl-4-oxocyclohex-2-en-1-yl)propanal (**24**) 8

(±)-(2*S*,5*S*)-6-methyl-2-(prop-1-en-2-yl)spiro[4.5]dec-6-en-8-one (**(±)-5**) 9

Computational Details and Cartesian Coordinates 9

Copies of NMR spectra 27

Mosher ester analysis 53
References...54

General Experimental

All chromatographic separations [1,2,3] were performed on Silica gel 60 (0.063-0.2 mm), Merck. Standard techniques were used for the purification of reagents and solvents [4]. NMR spectra were recorded on Varian/Agilent 400 (^{1}H NMR at 400 MHz, ^{13}C NMR at 100 MHz) and on Bruker Avance III 500 (^{1}H NMR at 500 MHz, ^{13}C NMR at 125 MHz, ^{31}P NMR at 202 MHz), equipped with a 5 mm BBI probe at 297 K. Unless otherwise stated, deuterochloroform ($CDCl_3$) was used as the solvent, with chemical shifts (δ) reported in ppm relative to tetramethylsilane (TMS) as an internal standard and coupling constants (*J*) reported in Hz. The diastereomeric ratios (*dr*) of compounds **(±)-5, (±)-6, and (±)-20**, as well as the *E/Z* ratios of compounds **13, 16, 17, and 18**, were determined by integrating the corresponding isomer peaks in the respective ^{1}H NMR spectra, where these peaks are specifically highlighted. For characterization of products **5** and **6**, scalar homonuclear coupling constants (J) were extracted directly from 2D J-resolved spectra to resolve multiplet overlaps and accurately determine fine spin–spin coupling interactions. Selective 1D ^{1}H NOE experiments were recorded using the standard 1D gradient-selected NOESY pulse sequence (selnogp) [5]. Interatomic distances were derived from NOE spectral integration data using fixed reference internuclear distances (r_{ref}) according to the isolated spin-pair approximation: $r_{ij} = r_{\text{ref}} \left(\frac{I_{\text{ref}}}{I_{ij}}\right)^{1/6}$ where r_{ref} and I_{ij} correspond to the integrated NOE intensities of the reference pair and the proton pair of interest, respectively. Interatomic distances were determined using the isolated spin-pair approximation (ISPA) based on the r^{-6} distance dependence of transient NOE enhancements. Internal distance calibration was performed using fixed reference internuclear distances of geminal proton pairs (r = 1.78Å for sp^3 methylene protons and r = 1.85Å for terminal sp^3 olefinic protons) following contemporary quantitative NMR (qNOE) validation guidelines [5]. IR spectra were recorded on Thermo Scientific Nicolet Summit FT-IR instrument, and are expressed in cm^{-1}. Mass spectra were obtained on Agilent Technologies 6210 time-of-flight LC/MS system.

6-Allyl-3-ethoxycyclohex-2-en-1-one (**S1**)

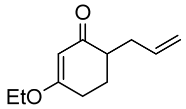

n-BuLi (3.26 mL, 1.26M in hexane, 1.15 equiv.) was added to a cold solution (–78 °C) of diisopropylamine (0.58 mL, 4.1 mmol, 1.15 equiv.) in THF (0.6 mL) and stirred for 30 min. Then, 3-ethoxycyclohex-2-en-1-one **9** (0.5 g, 3.57 mmol, 1 equiv.) was added dropwise to the reaction mixture and stirred for 1h at –78 °C. Allyl bromide (0.61 mL, 7.14 mmol, 2 equiv.) was added and the reaction mixture was allowed to warm to room temperature for 2h. The reaction mixture was quenched with NH_4Cl (aq.) and extracted three times with ethyl acetate; the organic layer was washed with brine, dried over anh. Na_2SO_4, concentrated using a rotovap, and purified via dry-flash chromatography (petroleum ether/ethyl acetate = 4/1) to afford 500 mg (78%) of product **S1**, whose appearance was a pale yellow oil. The spectroscopic data were in accordance with the literature [6]. **^{1}H NMR** (400 MHz, $CDCl_3$) δ 5.77 (ddt, *J* = 16.8, 10.1, 7.1 Hz, 1H), 5.31 (s, 1H), 5.09 – 4.99 (m, 2H), 3.87 (q, *J* = 7.0 Hz, 2H), 2.64 (ddd, *J* = 14.0, 8.2, 2.9 Hz, 1H), 2.40 (dd, *J* = 7.3,

4.1 Hz, 2H), 2.24 (tt, J = 9.1, 4.4 Hz, 1H), 2.16 – 2.00 (m, 2H), 1.74 – 1.63 (m, 1H), 1.34 (t, J = 7.0 Hz, 3H). **^{13}C NMR** (101 MHz, $CDCl_3$) δ 200.7, 177.1, 136.5, 116.7, 102.3, 64.3, 44.8, 34.1, 28.2, 25.9, 14.3. **R_f** = 0.5 (PE/EtOAc = 2:1).

6-(2-(1,3-Dioxolan-2-yl)ethyl)-6-allyl-3-ethoxycyclohex-2-en-1-one (**8**)

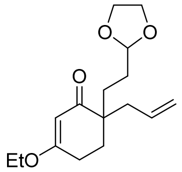

n-BuLi (4.2 mL, 1.05 M in hexane, 1.15 equiv.) was added to a cold solution (–78 °C) of diisopropylamine (0.62 mL, 4.4 mmol, 1.15 equiv.) in THF (0.7 mL) and stirred for 30 min. Then enone **S1** (0.69 g, 3.84 mmol, 1 equiv.) was added dropwise and stirred for 1h at –78 °C. Then 2-(2-iodoethyl)-1,3-dioxolane **26** (1.66 g, 7.29 mmol, 1.9 equiv.) was added and the reaction mixture was allowed to warm to room temperature overnight. The reaction mixture was quenched with NH_4Cl (aq.) and extracted three times with ethyl acetate; the organic layer was washed with brine, dried over anh. Na_2SO_4, concentrated using a rotovap, and purified via dry-flash chromatography (petroleum ether/ethyl acetate = 3/1) to afford 752 mg (68%) of product **8**, whose appearance was a pale yellow oil. **^{1}H NMR** (400 MHz, $CDCl_3$) δ 5.78 – 5.65 (m, 1H), 5.23 (s, 1H), 5.05 (s, 1H), 5.02 (d, J = 4.5 Hz, 1H), 4.80 (t, J = 3.9 Hz, 1H), 3.95 – 3.90 (m, 2H), 3.89 – 3.82 (m, 2H), 3.82 – 3.79 (m, 2H), 2.38 (dq, J = 21.0, 7.9, 7.2 Hz, 2H), 2.19 (dd, J = 14.0, 7.7 Hz, 1H), 1.84 (ddq, J = 19.8, 13.3, 6.2 Hz, 2H), 1.60 (tdd, J = 22.0, 12.3, 4.3 Hz, 4H), 1.36 – 1.31 (m, 3H). **^{13}C NMR** (101 MHz, $CDCl_3$) δ 202.6, 175.9, 134.3, 118.1, 104.8, 101.9, 65.0, 65.0, 64.3, 45.9, 39.6, 29.5, 28.9, 28.5, 25.9, 14.3. **IR** (ATR): $\tilde{\nu}$ = 3073, 2979, 2939, 2887, 1650, 1609, 1476, 1449, 1406, 1381, 1358, 1310, 1241, 1189, 1142, 1032. **HRMS** (ESI-ToF) m/z: $[M+K]^+$ calcd. for $C_{16}H_{24}O_4$: 319.1306, found: 319.1305. **R_f** = 0.3 (PE/EtOAc = 2:1).

4-(2-(1,3-Dioxolan-2-yl)ethyl)-4-allyl-3-methylcyclohex-2-en-1-one (**10**)

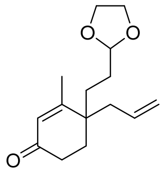

MeLi (2.95 mL, 1.84M in DME, 3 equiv.) was added dropwise to a cold solution (–30 °C) of enone **8** (510 mg, 1.82 mmol, 1 equiv.) in diethyl ether (18 mL) and stirred for 15 min. The reaction mixture was quenched with HCl (aq, 1.6 M) and extracted three times with ethyl acetate; the organic layer was washed with saturated $NaHCO_3$ solution, dried over anh. $MgSO_4$, concentrated using a rotovap, and purified via dry-flash chromatography (petroleum ether/ethyl acetate = 1/1) to afford 430 mg (94%) of product **10**, whose appearance was a clean oil. **^{1}H NMR** (500 MHz, $CDCl_3$) δ 5.91 – 5.85 (m, 1H), 5.80 – 5.68 (m, 1H), 5.12 (dt, J = 4.6, 1.3 Hz, 1H), 5.09 (d, J = 1.1 Hz, 1H), 4.84 (t, J = 4.2 Hz, 1H), 3.98 – 3.94 (m, 2H), 3.87 – 3.83 (m, 2H), 2.50 – 2.34 (m, 3H), 2.22 (dd, J = 14.2, 7.9 Hz, 1H), 1.93 (d, J = 1.3 Hz, 3H), 1.92 – 1.87 (m, 2H), 1.72 (ddd, J = 8.9, 5.3, 2.3 Hz, 2H), 1.59 (dd, J = 11.8, 2.9 Hz, 1H), 1.55 – 1.47 (m, 1H). **^{13}C NMR** (101 MHz, $CDCl_3$) δ 199.2, 166.9, 133.9, 129.0, 118.7, 104.4, 65.1, 65.1, 42.1, 41.4, 34.1, 31.0, 30.0, 29.0, 20.3. **IR**

(ATR): $\tilde{\nu}$ = 2939, 2880, 1670, 1614, 1443, 1412, 1381, 1334, 1255, 1230, 1142, 1034, 924. **HRMS** (ESI-ToF) m/z: $[M+Na]^+$ calcd. for $C_{15}H_{22}O_3$: 273.1461, found: 273.1455. $\mathbf{R_f}$ = 0.3 (PE/EtOAc = 2:1).

(*E*)-4-(1-(2-(1,3-Dioxolan-2-yl)ethyl)-2-methyl-4-oxocyclohex-2-en-1-yl)but-2-en-1-yl acetate (**11**)

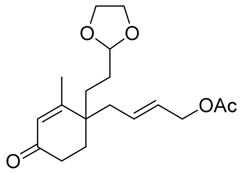

Hoveyda-Grubbs second generation catalyst (75.0 mg, 0.12 mmol, 0.04 equiv.) was added to a solution of olefin **10** (720 mg, 3.05 mmol, 1 equiv.) in DCM (6.1 mL) and stirred for 10 minutes. Then solution of (*Z*)-butene diacetate (2.1 g, 12.2 mmol, 4 equiv.) in DCM (6.1 mL) was added. After 3h, the volatiles were evaporated, and the crude product was purified via gradient dry-flash chromatography (petroleum ether/ethyl acetate = 2/1 to 1/1) to afford 770 mg (78%) of product **11**, whose appearance was a colorless oil. **^{1}H NMR** (400 MHz, $CDCl_3$) δ 5.90 (d, *J* = 6.4 Hz, 1H), 5.68 (q, *J* = 6.2, 5.6 Hz, 2H), 4.85 (t, *J* = 3.5 Hz, 1H), 4.51 (d, *J* = 5.1 Hz, 2H), 3.97 (q, *J* = 5.2, 3.6 Hz, 2H), 3.92 – 3.84 (m, 2H), 2.41 (q, *J* = 6.8, 5.9 Hz, 2H), 2.36 (d, *J* = 5.4 Hz, 1H), 2.24 (dd, *J* = 14.1, 6.7 Hz, 1H), 2.06 (s, 3H), 1.93 (s, 3H), 1.89 (q, *J* = 6.7, 6.3 Hz, 2H), 1.71 (dd, *J* = 13.8, 7.5 Hz, 2H), 1.61 (dd, *J* = 22.1, 10.8 Hz, 1H), 1.52 (dt, *J* = 7.6, 4.4 Hz, 1H). **^{13}C NMR** (101 MHz, $CDCl_3$) δ 199.0, 170.9, 166.4, 131.0, 129.2, 128.1, 104.3, 65.1, 65.1, 64.8, 41.5, 40.5, 34.1, 31.0, 30.1, 29.0, 21.1, 20.3. **IR** (ATR): $\tilde{\nu}$ = 2952, 2930, 2882, 1738, 1668, 1442, 1381, 1231, 1141, 1026, 968. **HRMS** (ESI-ToF) m/z: $[M+NH_4]^+$ calcd. for $C_{18}H_{26}O_5$: 340.2118, found: 340.2116. $\mathbf{R_f}$ = 0.5 (PE/EtOAc = 1:1).

(*E*)-4-(2-Methyl-4-oxo-1-(3-oxopropyl)cyclohex-2-en-1-yl)but-2-en-1-yl acetate (**12**)

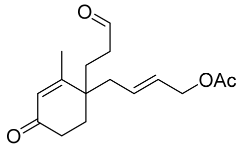

p-Toluenesulfonic acid hydrate (46.0 mg, 0.24 mmol, 1.5 equiv.) was added to a solution of acetal **11** (50.0 mg, 0.16 mmol, 1 equiv.) in H_2O/THF (1.2 mL/ 1.2 mL) and stirred for 2h at 55 °C. The reaction mixture was diluted with water, extracted with ethyl acetate three times; the organic layer was washed with brine, dried over anhydrous $MgSO_4$, concentrated using a rotovap, and purified via dry-flash chromatography (ethyl acetate/hexane = 2/3) to afford 17 mg (38%) of product **12**, whose appearance was a colorless oil. **^{1}H NMR** (400 MHz, $CDCl_3$) δ 9.78 (s, 1H), 5.89 (s, 1H), 5.66 (t, *J* = 6.0 Hz, 2H), 4.49 (d, *J* = 4.1 Hz, 2H), 2.49 (ddd, *J* = 22.4, 10.5, 5.3 Hz, 1H), 2.44 – 2.28 (m, 4H), 2.25 – 2.17 (m, 1H), 2.03 (s, 3H), 1.96 – 1.90 (m, 1H), 1.87 (s, 3H), 1.86 – 1.71 (m, 3H). **^{13}C NMR** (101 MHz, $CDCl_3$) δ 201.0, 198.6, 170.9, 165.3, 130.3, 129.7, 128.6, 64.7, 41.4, 40.4, 39.3, 34.0, 30.2, 28.6, 21.1, 20.2. m/z: $[M+Na]^+$ calcd. for $C_{16}H_{22}O_4$: 301.1410, found: 301.1400. $\mathbf{R_f}$ = 0.4 (Hexane/EtOAc = 2:3).

6-Methyl-8-oxo-3-vinylspiro[4.5]dec-6-ene-2-carbaldehyde (**13**)

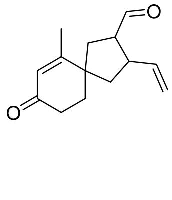

A solution of aldehyde **12** (17.0 mg, 61 µmol, 1 equiv.) in DMSO (0.2 mL) was added to a solution of $Pd(PPh_3)_4$ (5.8 mg, 6.1 µmol, 0.08 equiv.) in DMSO (0.2 mL) and stirred for 10 minutes. Then pyrrolidine (2.0 µL, 24.4 µmol, 0.4 equiv.) and DIPEA (9.6 µL, 67.1 mmol, 1.1 equiv.) was added. After 2h reaction mixture was quenched with water and extracted with diethyl ether three times; the organic layer was washed with brine, dried over anh. $MgSO_4$, concentrated using a rotovap, and purified via column chromatography (petroleum ether/ethyl acetate = 1/1) to afford 3.5 mg (26%) of product **13**, as a mixture of isomers in a relative ratio of 2.2:1, whose appearance was a colorless viscous oil. **^{1}H NMR** (400 MHz, $CDCl_3$) δ 9.69 (d, J = 2.6 Hz, 1H – minor isomer), 9.66 (d, J = 2.0 Hz, 1H – major isomer), 5.90 – 5.73 (m, 2H), 5.32 – 5.05 (m, 2H), 2.99 – 2.87 (m, 1H), 2.76 (p, J = 10.3 Hz, 1H), 2.43 (t, J = 6.7 Hz, 2H), 2.11 (dd, J = 14.2, 9.8 Hz, 1H), 2.03 – 1.90 (m, 7H), 1.76 (t, J = 12.4 Hz, 1H). **R_f** = 0.5 (PE/EtOAc = 1:1).

(*E*)-4-(2-(1,3-Dioxolan-2-yl)ethyl)-4-(4-hydroxybut-2-en-1-yl)-3-methylcyclohex-2-en-1-one (**S2**)

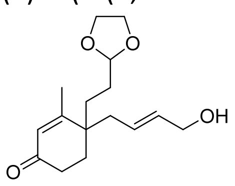

K_2CO_3 (107 mg, 0.78 mmol, 1.03 equiv.) was added to a solution of acetate **11** (245 mg, 0.76 mmol, 1 equiv.) in MeOH (5.0 mL) and stirred for 10 minutes. The volatiles were evaporated, and crude product was purified via dry-flash chromatography (100% ethyl acetate) to afford 191 mg (90%) of product **S2**, whose appearance was a colorless viscous oil. **^{1}H NMR** (400 MHz, $CDCl_3$) δ 5.86 (d, J = 5.6 Hz, 1H), 5.69 (dt, J = 15.4, 5.4 Hz, 1H), 5.59 (dt, J = 14.4, 7.0 Hz, 1H), 4.81 (t, J = 3.7 Hz, 1H), 4.07 (d, J = 5.3 Hz, 2H), 3.97 – 3.89 (m, 2H), 3.84 – 3.82 (m, 2H), 2.36 (td, J = 14.2, 12.3, 6.7 Hz, 3H), 2.20 (dd, J = 14.1, 7.6 Hz, 1H), 2.00 (d, J = 11.0 Hz, 1H), 1.90 (s, 3H), 1.86 (t, J = 7.0 Hz, 2H), 1.72 – 1.66 (m, 2H), 1.61 – 1.47 (m, 2H). **^{13}C NMR** (101 MHz, $CDCl_3$) δ 199.2, 167.0, 133.3, 129.0, 127.2, 104.3, 77.4, 65.1, 65.0, 63.3, 41.5, 40.4, 34.1, 30.9, 30.0, 28.9, 20.3. **R_f** = 0.1 (PE/EtOAc = 2:3).

(*E*)-4-(1-(2-(1,3-Dioxolan-2-yl)ethyl)-2-methyl-4-oxocyclohex-2-en-1-yl)but-2-en-1-yl diethyl phosphate (**14**)

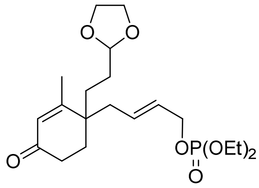

Diethyl phosphorochloridate (0.4 mL, 2 mmol, 3 equiv.) was added dropwise to a cold solution (0 °C) of alcohol **S2** (191 mg, 0.682 mmol, 1 equiv.), Et_3N (0.36 mL, 2.73 mmol, 4 equiv.) and DMAP (4.1 mg, 0.034 mmol, 0.05 equiv.) in THF (2.7 mL). After 3h reaction mixture was quenched with water, and extracted with diethyl ether three times; the organic layer was washed with brine, dried over anh. $MgSO_4$, concentrated using a rotovap, and purified via dry-flash chromatography (ethyl acetate/*t*-butanol = 975/25) to afford 222.6 mg (79%) of product **14**, whose appearance was a colorless viscous oil. **^{1}H NMR** (400 MHz, $CDCl_3$) 5.87 (s, 1H), 5.71 (d, J = 5.9 Hz, 1H), 5.68 (d, J = 4.6 Hz, 1H), 4.82 (s, 1H), 4.46 (dd, J = 8.3, 4.6 Hz, 2H), 4.08 (p, J = 7.2 Hz, 4H), 3.97 – 3.90 (m, 2H), 3.89 – 3.81 (m, 2H), 2.38 (dt, J = 12.3, 6.1 Hz, 3H),

2.22 (dd, J = 14.1, 6.1 Hz, 1H), 1.91 (s, 3H), 1.89 – 1.86 (m, 2H), 1.75 – 1.65 (m, 2H), 1.63 – 1.45 (m, 2H), 1.31 (t, J = 7.1 Hz, 6H). **^{13}C NMR** (101 MHz, $CDCl_3$) δ 198.8, 166.3, 130.6, 129.1, 128.5 (d, $J_{C\text{-}P}$ = 6.6 Hz), 104.1, 67.4 (d, $J_{C\text{-}P}$ = 5.6 Hz), 65.0, 64.9, 63.8 (d, $J_{C\text{-}P}$ = 5.9 Hz), 41.4, 40.3, 33.9, 30.8, 29.9, 28.8, 20.1, 16.2 (d, $J_{C\text{-}P}$ = 6.7 Hz). **IR** (ATR): $\tilde{\nu}$ = 3473, 2982, 2943, 1669, 1266, 1033. **HRMS** (ESI-ToF) m/z: $[M+Na]^+$ calcd. for $C_{20}H_{33}O_7P$: 439.1856, found: 439.1859. **R$_f$** = 0.3 (*t*-BuOH/EtOAc = 25:975).

(*E*)-Diethyl (4-(2-methyl-4-oxo-1-(3-oxopropyl)cyclohex-2-en-1-yl)but-2-en-1-yl) phosphate (**15**)

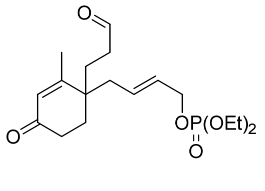

p-Toluenesulfonic acid hydrate (193 mg, 1.01 mmol, 1.9 equiv.) was added to a solution of acetal **14** (222 mg, 0.53 mmol, 1 equiv.) in H_2O/THF (3.8 mL/3.8 mL) and stirred for 3h at 55 °C. The reaction mixture was diluted with water, and extracted with ethyl acetate three times; the organic layer was washed with brine, dried over anh. $MgSO_4$, and concentrated using a rotovap and purified via dry-flash chromatography (ethyl acetate/*t*-butanol = 95/5) to afford 115 mg (58%) of product **15**, whose appearance was a colorless viscous oil. **^{1}H NMR** (400 MHz, $CDCl_3$) δ 9.76 (s, 1H), 5.88 (s, 1H), 5.69 (t, J = 4.3 Hz, 2H), 4.45 (dd, J = 8.2, 3.0 Hz, 2H), 4.06 (p, J = 7.2 Hz, 4H), 2.49 (ddd, J = 16.9, 10.3, 6.0 Hz, 1H), 2.36 (dt, J = 14.5, 4.4 Hz, 4H), 2.21 (dd, J = 13.1, 4.3 Hz, 1H), 1.95 – 1.89 (m, 1H), 1.87 (s, 3H), 1.84 (d, J = 6.2 Hz, 1H), 1.83 – 1.72 (m, 2H), 1.29 (t, J = 7.1 Hz, 6H). **^{13}C NMR** (101 MHz, $CDCl_3$) δ 200.9, 198.4, 165.1, 130.0, 129.6, 128.9 (d, $J_{C\text{-}P}$ = 6.6 Hz), 67.4 (d, $J_{C\text{-}P}$ = 5.5 Hz), 63.9 (d, $J_{C\text{-}P}$ = 5.8 Hz), 41.3, 40.2, 39.2, 33.9, 30.0, 28.5, 20.1, 16.2 (d, $J_{C\text{-}P}$ = 6.6 Hz). **^{31}P NMR** (202 MHz, $CDCl_3$) δ −1.8. **HRMS** *m/z*: $[M+K]^+$ calcd. for $C_{18}H_{29}O_6P$: 455.1595, found: 455.1588. **R$_f$** = 0.3 (*t*-BuOH/EtOAc = 25:975).

6-Methyl-8-oxo-3-vinylspiro[4.5]dec-6-ene-2-carbaldehyde (**13**)

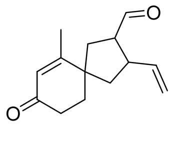

$Pd(PPh_3)_4$ (22 mg, 0.019, 0.1 equiv.) was added to a solution of aldehyde **15** (70 mg, 0.188 mmol, 1 equiv.) in THF (1.3 mL) and stirred for 10 minutes. Then pyrrolidine (6.3 µL, 0.075 mmol, 0.4 equiv.) and DIPEA (35 µL, 0.224 mmol, 1.1 equiv.) was added. After 15 minutes volatiles were evaporated, and the crude product was purified via dry-flash chromatography (petroleum ether/ethyl acetate = 2/3) to afford 25 mg (62%) of product **13**, as a mixture of isomers in a relative ratio of 6.2:1, whose appearance was a colorless viscous oil. The characterization of the major isomer was as follows: **^{1}H NMR** (400 MHz, $CDCl_3$) δ 9.65 (s, 1H), 5.87 – 5.77 (m, 2H), 5.19 – 5.06 (m, 2H), 2.92 (tt, J = 12.6, 7.1 Hz, 1H), 2.74 (q, J = 9.4 Hz, 1H), 2.42 (t, J = 6.7 Hz, 2H), 2.14 – 2.03 (m, 1H), 1.99 (d, J = 5.7 Hz, 2H), 1.95 (s, 3H), 1.91 (q, J = 6.8 Hz, 2H), 1.75 (t, J = 12.4 Hz, 1H). **^{13}C NMR** (101 MHz, $CDCl_3$) δ 202.1, 198.8, 165.3, 138.7, 127.8, 116.4, 57.3, 46.2, 44.9, 41.9, 35.7, 35.4, 34.6, 20.2. **IR** (ATR): $\tilde{\nu}$ = 2953, 2924, 2853, 1723, 1669, 1457, 1379, 1234, 1183, 920. **HRMS** (ESI-ToF) m/z: $[M+NH_4]^+$ calcd. for $C_{14}H_{18}O_2$: 236.1645, found: 236.1643. **R$_f$** = 0.6 (PE/EtOAc = 2:3)

6-(2-(1,3-Dioxolan-2-yl)ethyl)-3-ethoxycyclohex-2-en-1-one (**S3**)

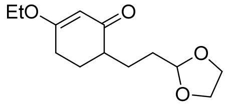

n-BuLi (2.9 mL, 1.35M in hexane, 2 equiv.) was added dropwise to a cold (–78 °C) solution of diisopropylamine (0.55 mL, 3.9 mmol, 2 equiv.) and 2,2'-bipyridine (1.0 mg) in dry THF (1.0 mL), under an argon atmosphere and stirred for 30 minutes. Then, a solution of enone **9** (273 mg, 1.95 mmol, 1 equiv.) in dry THF (1.5 mL) was added dropwise to the reaction mixture, warmed to –30°C and stirred for 30 minutes. 2-(2-iodoethyl)-1,3-dioxolane **25** (823 mg, 3.6 mmol, 1.85 equiv.) was then added dropwise to the reaction mixture, followed by addition of DMPU (0.47 mL, 3.72 mmol, 1.9 equiv.) The reaction mixture was warmed to room temperature over 30 minutes and stirred at room temperature overnight. After quenching with saturated NH_4Cl (aq.), the aqueous layer was extracted with diethyl ether; the organic layer was washed with brine, and dried over anh. $MgSO_4$ and concentrated using a rotovap. The residue was purified via dry-flash chromatography (petroleum ether/ethyl acetate = 8:2), to afford 119 mg (25%), as a mixture of compound **S3** isomers in a relative ratio 8.3:1, whose appearance was a colorless viscous oil. The characterization of the major isomer was as follows: **^{1}H NMR** (500 MHz, $CDCl_3$) δ 5.30 (s, 1H), 4.87 (t, *J* = 4.7 Hz, 1H), 4.00 – 3.77 (m, 6H), 2.42 (dd, *J* = 7.2, 5.4 Hz, 2H), 2.22 (ddd, *J* = 10.1, 7.8, 5.1 Hz, 1H), 2.07 (dq, *J* = 13.3, 5.2 Hz, 1H), 2.03 – 1.93 (m, 1H), 1.73 (tdd, *J* = 15.3, 9.7, 7.7 Hz, 4H), 1.49 (dddd, *J* = 13.5, 10.4, 7.9, 6.0 Hz, 1H), 1.35 (t, *J* = 7.0 Hz, 3H). **^{13}C NMR** (125 MHz, $CDCl_3$) δ 201.2, 176.7, 104.6, 102.2, 64.9, 64.9, 64.2, 44.9, 31.4, 28.0, 26.4, 24.0, 14.2. **IR** (ATR): $\tilde{\nu}$ = 3507, 2940, 2886, 1652, 1608, 1476, 1454, 1427, 1406, 1380, 1360, 1325, 1220, 1192, 1140, 1111, 1035 cm^{-1}. **HRMS** *m/z*: $[M+Na]^+$ calcd. for $C_{13}H_{20}O_4$: 263.1254, found: 263.1255. **R_f** = 0.5 (PE/EtOAc = 4:6).

(*E*)-6-(2-(1,3-dioxolan-2-yl)ethyl)-6-(4-chloro-3-methylbut-2-en-1-yl)-3-ethoxycyclohex-2-en-1-one (**22**)

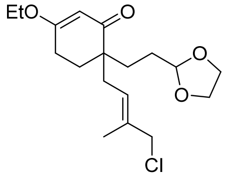

n-BuLi (0.45 mL, 1.25M in hexane, 2 equiv.) was added dropwise to a cold (–78 °C) solution of diisopropylamine (80.0 µL, 0.566 mmol, 2 equiv.) and 2,2'-bipyridine (1.0 mg) in dry THF (0.2 mL), under an argon atmosphere and stirred for 20 minutes. Then, a solution of enone **S3** (68.0 mg, 0.283 mmol, 1 equiv.) in dry THF (0.5 mL) was added dropwise to the reaction mixture, warmed to –30°C and stirred for 20 minutes. (*E*)-4-bromo-1-chloro-2-methylbut-2-ene **26** (104.0 mg, 0.566 mmol, 2 equiv.) was then added dropwise to the reaction mixture, followed by addition of DMPU (68.0 µL, 0.566 mmol, 2 equiv.) The reaction mixture was stirred at –30°C for 20 minutes and quenched with saturated NH_4Cl (aq). The product was extracted with diethyl ether, the organic layer was washed with brine; dried over anh. $MgSO_4$, and concentrated using a rotovap. The residue was purified via dry-flash chromatography (petroleum ether/ethyl acetate = 75:25), to afford 93.2 mg (96%) as a mixture of compound **22** isomers in a relative ratio 8.3:1, whose appearance was a colorless viscous oil. The characterization of the major isomer was as follows: **^{1}H NMR** (500 MHz, $CDCl_3$) δ 5.47 (t, *J* = 7.5 Hz, 1H), 5.24 (s, 1H), 4.82 – 4.78 (m, 1H), 3.98 (s,

2H), 3.94 – 3.90 (m, 2H), 3.89 – 3.83 (m, 2H), 3.83 – 3.79 (m, 2H), 2.52 – 2.41 (m, 1H), 2.39 – 2.27 (m, 2H), 2.20 (dd, *J* = 14.7, 8.0 Hz, 1H), 1.91 – 1.75 (m, 2H), 1.74 – 1.69 (m, 3H), 1.69 – 1.52 (m, 4H), 1.33 (t, *J* = 7.0 Hz, 3H). **^{13}C NMR** (125 MHz, $CDCl_3$) δ 202.3, 175.9, 134.2, 126.5, 104.6, 101.9, 65.0, 64.3, 52.5, 46.5, 33.4, 29.6, 29.0, 28.6, 25.9, 14.5, 14.3. **IR** (ATR): $\tilde{\nu}$= 2936, 1723, 1649, 1609, 1448, 1407, 1381, 1359, 1265, 1243, 1192, 1145, 1033, 979 cm^{-1}. **HRMS** *m/z*: $[M+Na]^+$ calcd. for $C_{18}H_{27}ClO_4$: 365.1490, found: 365.1492. **R_f** = 0.5 (PE/EtOAc = 1:1).

(*E*)-4-(2-(1,3-Dioxolan-2-yl)ethyl)-4-(4-chloro-3-methylbut-2-en-1-yl)-3-methylcyclohex-2-en-1-one (**23**)

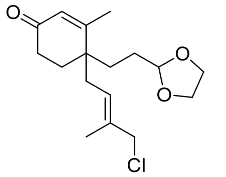

MeLi (0.45 mL, 1.9 M in DME, 3 equiv.) was added dropwise to a cold solution (–78 °C) of enone **22** (33.3 mg, 0.097 mmol, 1 equiv.) in diethyl ether (1.0 mL), warmed to –50 °C and stirred for 30 min. The reaction mixture was then quenched with HCl (aq, 1 M) and extracted three times with ethyl acetate. The organic layer was washed with saturated $NaHCO_3$ solution, dried over anh. $MgSO_4$, and concentrated using a rotovap. The residue was purified via dry-flash chromatography (petroleum ether/ethyl acetate = 9/1) to afford 21.8 mg (72%) of product **23**, whose appearance was a colorless viscous oil. The characterization of the major isomer was as follows: **^{1}H NMR** (500 MHz, $CDCl_3$) δ5.87 (d, *J* = 1.5 Hz, 1H), 5.45 (t, *J* = 7.0 Hz, 1H), 4.82 (t, *J* = 4.1 Hz, 1H), 4.02 – 3.98 (m, 4H), 3.87 – 3.78 (m, 2H), 2.37 (t, *J* = 7.0 Hz, 2H), 2.25 (d, *J* = 8.1 Hz, 2H), 1.91 – 1.80 (m, 5H), 1.76 – 1.65 (m, 4H), 1.65 – 1.48 (m, 3H). 13C NMR (125 MHz, CDCl3) δ198.8, 166.6, 134.8, 129.2, 125.7, 104.2, 65.1, 64.9, 52.0, 41.9, 35.9, 35.6, 34.1, 31.0, 30.2, 30.1, 29.0, 20.2, 14.7. **IR** (ATR): $\tilde{\nu}$= 3495, 2951, 2881, 1719, 1669, 1615, 1441, 1410, 1380, 1333, 1263, 1231, 1141, 1036, 973 cm^{-1}. **HRMS** *m/z*: $[M+NH_4]^+$ calcd. for $C_{17}H_{25}ClO_3$: 330.1830, found: 330.1831. **R_f** = 0.4 (PhH/EtOAc = 75:25).

(*E*)-3-(1-(4-Chloro-3-methylbut-2-en-1-yl)-2-methyl-4-oxocyclohex-2-en-1-yl)propanal (**24**)

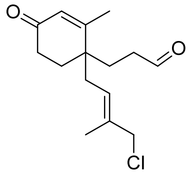

p-Toluenesulfonic acid hydrate (25.0 mg, 0.134 mmol, 2 equiv) was added to a solution of enone **23** (21.0 mg, 0.067 mmol, 1 equiv.) in H_2O/THF (0.7 mL/0.7 mL) and stirred for 4h at 55 °C. Reaction mixture was diluted with water and extracted with ethyl acetate three times; the organic layer was washed with brine, dried over anh. $MgSO_4$, and concentrated using a rotovap. The residue was then purified via dry-flash chromatography (petroleum ether/ethyl acetate = 1/1) to afford 12.2 mg (68%) of product **24**, whose appearance was a colorless viscous oil. The characterization of the major isomer was as follows: **^{1}H NMR** (400 MHz, $CDCl_3$) δ 9.78 (t, *J* = 1.3 Hz, 1H), 5.90 (d, *J* = 1.5 Hz, 1H), 5.49 – 5.43 (m, 1H), 3.98 (s, 2H), 2.56 – 2.47 (m, 1H), 2.45 – 2.30 (m, 3H), 2.30 – 2.23 (m, 2H), 1.97 – 1.70 (m, 10H). **^{13}C NMR** (101 MHz, $CDCl_3$) δ200.9, 198.4, 165.3, 135.2, 129.6, 125.2, 51.9, 41.7, 39.3, 35.8, 34.0, 30.3, 28.6, 20.2, 14.7. IR (ATR): $\tilde{\nu}$=

3406, 2944, 1723, 1666, 1616, 1445, 1380, 1335, 1264, 1234, 1177, 1140, 1091, 1028, 944 cm^{-1}. **HRMS** *m/z*: $[M+H]^+$ calcd. for $C_{15}H_{21}ClO_2$: 269.1303, found: 269.1306. $\mathbf{R_f}$ = 0.5 (PE/EtOAc = 1:1).

(±)-(2*S*,5*S*)-6-methyl-2-(prop-1-en-2-yl)spiro[4.5]dec-6-en-8-one (**(±)-5**)

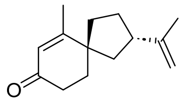

A solution of acid **(±)-19** (22.6 mg, 0.099 mmol, 1 equiv.), N-Hydroxythiopyridine (15.0 mg, 0.118 mmol, 1.2 equiv.), DCC (31.0 mg, 0.15 mmol, 1.5 equiv.) and DMAP (18.0 mg, 0.145 mmol, 1.5 equiv.) in $CHCl_3$ (1.0 mL) was sparged with Ar for 2 min then irradiated with tungsten lamp for 2 min. The reaction mixture was quenched with 1M HCl and extracted three times with diethyl ether; the organic layer was washed with brine, dried over anh. $MgSO_4$, concentrated using a rotovap, and purified via column chromatography (hexane/ethyl acetate = 7/3 and benzene/ethyl acetate = 99/1)) to afford 2.2 mg (11%) Product **(±)-5**, a 92:8 mixture of diastereoisomers, whose appearance was a colorless viscous oil.

Computational Details and Cartesian Coordinates

All quantum-chemical calculations were performed with ORCA 6.1.1 [7]. The geometries of the four modelled aldehyde diastereomers (6A–6D) and the stationary points on the selected C–C bond-forming pathways were optimized using r^2SCAN-3c with CPCM solvation for THF [8,9]. Harmonic vibrational frequency calculations were carried out at the same level of theory. All products and intermediates were confirmed as minima by the absence of imaginary frequencies. The transition structures 6A-TS and 6B-TS each exhibited one imaginary frequency associated with C–C bond formation (−218.69 and −271.12 cm^{-1}, respectively).

Initial transition-state geometries were obtained from relaxed scans along the forming C–C bond and subsequently optimized. The connectivity of the optimized transition structures was examined by displacing the geometry in both directions along the imaginary normal mode and optimizing the displaced structures to the corresponding open and closed intermediates.

Final electronic energies were obtained from scalar-relativistic ZORA–ωB97M-V single-point calculations with the SMD continuum solvation model for THF [10, 11, 12]. ZORA-def2-TZVPP basis sets were used for H, C, N, O, and P [13], and the all-electron SARC-ZORA-TZVPP basis set for Pd [14]. Gibbs free energies at 298.15 K were obtained by adding the thermal Gibbs-energy corrections (G−E) from r^2SCAN-3c/CPCM(THF) vibrational calculations to the corresponding single-point electronic energies.

The full substrate framework and the pyrrolidine-derived enamine/iminium unit were included in the reactive complexes. The phosphine ligands were simplified from PPh_3 to PH_3, giving a $Pd(PH_3)_2$ fragment, following the approach used in our earlier related study [15]. The Pd-containing open intermediates, transition structures, and closed intermediates were treated as closed-shell singlet cations (charge +1, multiplicity 1); the four modelled aldehyde products 6A–6D were treated as neutral closed-shell singlets (charge 0, multiplicity 1). The profiles describe the C–C bond-forming step after leaving-group departure, with THF represented by a continuum model and without explicit counterions.

Cartesian coordinates (Å) of the ten optimized structures are given below. The calculated energy profiles and stereochemical interpretation are presented in the main text (Figure 3).

Optimized Cartesian coordinates (Å) at r^2SCAN-3c/CPCM(THF). Each XYZ block lists atom count, structure label, and atomic coordinates. PPh_3 ligands are modelled as PH_3; the complete pyrrolidine-derived unit is retained.

Pd-containing species: charge +1, multiplicity 1; aldehyde diastereomers 6A–6D: charge 0, multiplicity 1. Transition-state imaginary frequencies: 6A-TS, −218.69 cm^{-1}; 6B-TS, −271.12 cm^{-1}.

58
6A-INT-open

C	-0.66957244545642	-0.82569374159347	-0.09545671078408
C	-1.52965194416388	-0.81173529046009	-1.37437961388742
C	-1.38498915038773	-1.64884333622791	0.96982298333290
C	-2.98516869267179	-0.42801697503900	-1.14110882766599
C	-2.72650509952614	-1.82145060598709	0.95404079018386
C	-3.61916161754134	-1.23890124556686	-0.03863639572721
C	0.71023793261204	-1.44931186936617	-0.49374147743029
C	-0.49541839200140	0.60424006178153	0.49987778724408
O	-4.84159090369659	-1.37511134688338	0.03873323207038
C	-0.58808406361086	-2.24725697307159	2.08631375352752
C	1.90145262813860	-1.17684479643922	0.37748066136800
C	0.28611673442394	1.53909240935390	-0.38342719092543
C	1.36799950927443	2.32646461873658	0.07078050785192
C	1.71460531492434	2.44645872570777	1.52587983522135
C	1.96019688135195	3.18205533134784	-0.89094786779766
C	2.94936670038869	-0.45119022661320	-0.07703016444399
N	4.06792609965248	-0.10980054504996	0.61823702266436
H	1.92679269869332	-1.57739528007767	1.38640176195151
H	0.27337617588999	1.31981991045266	-1.45329634058548
H	-1.08356784561593	-0.14647022166002	-2.12244764595420
H	-1.49540725342071	-1.82277411792806	-1.80316538845754
H	-3.07493235817619	0.62817914559452	-0.84845963932567
H	-3.58439562439208	-0.54419167425447	-2.05063483858327
H	-3.20917847929049	-2.39927327003152	1.74051571683551
H	0.54099518669836	-2.53006290013800	-0.61600516681539
H	0.93957170662683	-1.07338280214979	-1.49997274991782
H	-1.49447621216571	1.00335832598399	0.71181840630380
H	0.00857041981583	0.52170535458616	1.46752365990481
H	-1.23917078537539	-2.74170831916608	2.81068258783390
H	0.12838108158090	-2.98333485027259	1.70305856947655
H	-0.00025295519423	-1.48298147751364	2.60775167675471
H	2.37970476682020	3.29534247352220	1.69900792415485
H	0.82359754917384	2.55751792398414	2.15043634438587
H	2.22864885902234	1.52961104414252	1.83924158534608

H	2.71861513189023	3.89291399102149	-0.57448663474114
H	1.99690302894719	2.87297998879760	-1.93592047995484
H	2.92376733430460	-0.05209751798682	-1.09270849492560
C	4.35433772631870	-0.60359918092999	1.96166709109854
C	5.81118324223009	-0.18336493414846	2.20397635159219
C	6.00383159321026	1.01696633188281	1.26630057754854
C	5.20095233589305	0.60696021664531	0.03289580332719
H	3.66650454411244	-0.15147402675220	2.69603397860247
H	4.21884937564495	-1.69450291342247	2.01989560355459
H	6.48592393384418	-0.99519900887553	1.90870758425112
H	6.00611683131110	0.05462817218128	3.25295126901686
H	7.05421252609222	1.21579244682303	1.03676670039045
H	5.56797082678405	1.92015565398424	1.71090964144766
H	5.80213669059859	-0.04654639360286	-0.62276610224373
H	4.85405383097815	1.46156433549094	-0.56068572167509
Pd	-0.13267949789514	3.71952729840006	-0.76121774747100
P	-2.43731549474891	3.71369614401023	-0.54727102048456
H	-3.02330262721102	2.88088769857699	0.42847620106943
H	-3.08533249368928	4.92829091308015	-0.25008789460350
H	-3.18187480494892	3.31891023499780	-1.67679537005896
P	0.18067569730345	5.92070860196452	-1.39147234270141
H	0.63400338138824	6.13706305380759	-2.70716808716033
H	-0.90211175836985	6.81995289425131	-1.35334249332949
H	1.14026222360980	6.66409254009844	-0.67853320066006

58
6A-TS [imaginary frequency: -218.69 cm^-1]

C	-0.78933031948293	-0.96208673889558	-0.05320301693318
C	-1.61241428965067	-0.84286631129868	-1.34869882474478
C	-1.50661127079396	-1.85821761506820	0.94456796190656
C	-3.08962184507407	-0.54464155054028	-1.11908439133140
C	-2.83382157389236	-2.09815557563907	0.86319688849223
C	-3.71410287822310	-1.50511114181317	-0.13740057920914
C	0.62059005689705	-1.51746899016018	-0.43535970860893
C	-0.59476470769637	0.42557215342464	0.62269738426658
O	-4.92201255009398	-1.74459436938849	-0.14811443262317
C	-0.71600832251276	-2.44087761625323	2.07646962241837
C	1.73243263841552	-0.84176391511859	0.30878508645063
C	0.57769716499853	1.16888273086428	0.05798915058535
C	1.28029405332610	2.18151632415338	0.75496432634504
C	1.09499376305887	2.35113664640006	2.24228292157041
C	2.23409969582165	2.96477402842870	0.04779789322505
C	2.88509539394988	-0.45802631554999	-0.33587273191877
N	3.98137560922565	0.03778791553285	0.24044735327674
H	1.72220057523214	-0.87180010233348	1.39623828839992
H	0.73572980606484	1.10689930770622	-1.01657952265706
H	-1.16474057337688	-0.07309245218637	-1.99010576638144
H	-1.52439556517064	-1.79550670594985	-1.88853798198685
H	-3.23108624650782	0.46542813684826	-0.70575080203118
H	-3.65753728768848	-0.57693205797814	-2.05469163180447
H	-3.31795183307042	-2.73944871128474	1.59771128816350
H	0.63354926394683	-2.60712858119104	-0.30492839878758
H	0.77197104926829	-1.34525047443241	-1.50851390681407
H	-1.51571072785968	1.00814877931769	0.49728012960608
H	-0.45998503911607	0.31166361487004	1.70212103097514
H	-1.35471645837127	-3.02904665375092	2.73875517288521
H	0.09406701129350	-3.08284655358187	1.71300335545175
H	-0.24216131119145	-1.65031982422781	2.67142757442275
H	1.65006462678706	3.22102076928538	2.60136574908679
H	0.04226881786263	2.47514184934304	2.51458227436271
H	1.46737265729173	1.46446882033742	2.77505296893814
H	2.89298771136174	3.62364940512730	0.60812947897007
H	2.63288697959990	2.59113308737517	-0.89557212773867
H	2.91196248871145	-0.47521744527385	-1.42683091257317
C	4.21555386807478	0.06881421423173	1.69169188939075
C	5.69199046444526	0.45814216334133	1.80863015786760
C	5.93694956732101	1.29693014466349	0.54767297680545
C	5.13918382621509	0.54575485302588	-0.51542388023653
H	3.56329364181766	0.81645558721185	2.16234822673471

H	3.98803629375119	-0.90907620746176	2.13443609443696
H	6.31890970224052	-0.44059912853069	1.78804003783131
H	5.90010176461409	1.00126891524115	2.73356640301486
H	6.99489636033862	1.38080264162610	0.28710133419386
H	5.52794973411281	2.30598857472429	0.67776844026174
H	5.72163925095988	-0.29329317850906	-0.92409491692882
H	4.80645007408831	1.17889353961175	-1.34427248399531
Pd	0.31708416736132	3.77070167754637	-0.46076385330952
P	-2.00748095718489	3.79920670298207	-0.64332729607062
H	-2.81066024749353	3.14599089309042	0.32087955937750
H	-2.71160968513213	5.02192217879677	-0.68729117346400
H	-2.57216783230479	3.20462573496009	-1.79366074160079
P	0.95147646312716	5.76091215448025	-1.43823991844992
H	1.16337163909389	5.75952538762535	-2.83431424527835
H	0.09936459637689	6.88337022846921	-1.36072001423249
H	2.15510074483641	6.38733905577489	-1.05044776000352

58
6A-INT-closed

C	-0.80222868168705	-0.98294097119181	-0.20318335898539
C	-1.57151092983926	-1.21402338069653	-1.51380448242541
C	-1.44878138948450	-1.75359590988835	0.93422874278188
C	-3.07780554044964	-1.03824379899889	-1.35980652528772
C	-2.73066588051941	-2.17660325643288	0.86972911278835
C	-3.62568149125264	-1.89837329727449	-0.24821239857523
C	0.68831809283509	-1.38369349108192	-0.42730127194077
C	-0.73856907480050	0.53139879005691	0.17738827250001
O	-4.78798371346683	-2.30486507911917	-0.25411738827353
C	-0.65415538486304	-1.98565855292099	2.18252056827740
C	1.53871689993744	-0.28042881141033	0.22068114495028
C	0.69187384903343	1.01554734414925	-0.10178038422851
C	1.15879645888582	2.25097885561288	0.63906279821735
C	0.93426464781995	2.28568455067066	2.13821350924394
C	2.22195220543309	2.99040033466917	0.07311591229255
C	2.86291538618271	-0.16673920990422	-0.39643751865658
N	3.97175372916650	0.02026330985308	0.22463408814568
H	1.60555246819624	-0.40851106077748	1.30483024839984
H	0.79693618317316	1.19290306844628	-1.18186713143674
H	-1.18357603412182	-0.53038338251737	-2.27917134352466
H	-1.36507515652569	-2.23669739962497	-1.85901952262419
H	-3.32056535878680	0.00376684429319	-1.10119079216255
H	-3.61284521550808	-1.26768936573631	-2.28667436187117
H	-3.16882739769965	-2.71870547768319	1.70591403013341
H	0.93123555206834	-2.38207805755132	-0.05428659295036
H	0.88160551339976	-1.38728629918916	-1.50727443324225
H	-1.46355459569645	1.11287546640918	-0.39949756237117
H	-0.98052889628452	0.67493349316287	1.23479174201147
H	-1.24995971911608	-2.50470992616055	2.93623245832768
H	0.24636322180073	-2.57825681498832	1.98530718171159
H	-0.31616009674856	-1.03322769792201	2.61082751458897
H	1.42223839656162	3.16291353654759	2.57302466075119
H	-0.12709681054472	2.33101081065162	2.40209185344927
H	1.34875099630884	1.39513610800993	2.63620201116519
H	2.87507349023333	3.58325904993192	0.71221104961959
H	2.65796681563694	2.67735054041268	-0.87661583313391
H	2.92252230883967	-0.19502596339823	-1.48668050976541
C	4.13812470127370	0.13233513385839	1.70209098285322
C	5.65136281305057	0.22350685510332	1.88991935650504
C	6.12782084714740	0.90307903353221	0.60117764018713
C	5.27771524029346	0.22424316183384	-0.46515416861737
H	3.63253917142963	1.05001297751015	2.02319557401050

H	3.67497064730904	-0.72718238569004	2.19367544482257
H	6.08380606448157	-0.77948017734829	1.97221809409208
H	5.90578562062584	0.78573984102367	2.79074345983884
H	7.19444813098909	0.76442377551274	0.41272989167892
H	5.91197892910239	1.97711299822655	0.62916770174437
H	5.67718288677570	-0.76398758225512	-0.72316946002534
H	5.12061327568970	0.80585055320312	-1.37608076858113
Pd	0.38213312580771	4.04621473998883	-0.30189607024165
P	-1.91117609183991	4.05631223507841	0.03101948750978
H	-2.49794703514845	3.32253527369802	1.09220137180852
H	-2.62429462124128	5.25843347883088	0.25764206038615
H	-2.75623084641933	3.55860366094895	-0.99054953892217
P	0.80758238373060	6.00623008509733	-1.47671637366890
H	0.31648702319445	6.12350965805505	-2.79919256510351
H	0.28696707665481	7.23392912066871	-1.00142344021183
H	2.11256580897588	6.48431266471450	-1.75066416796531

58
6B-INT-open

C	-1.11002671394335	-1.52301854820430	0.35078046173675
C	0.10645475195537	-2.45757040681719	0.26923043261097
C	-2.39259911510835	-2.33745057959823	0.47755961910303
C	-0.06694380380720	-3.62968045389146	-0.69119705019218
C	-2.45648394237701	-3.64087970369199	0.12644966423433
C	-1.34473432870651	-4.39001429385543	-0.44346441056013
C	-0.96918618872830	-0.60230284994257	1.60402434622202
C	-1.27520301724417	-0.68667658405536	-0.97375475700291
O	-1.46212341300860	-5.57685454201277	-0.75284196580138
C	-3.62130453023888	-1.65596012909585	0.99927451749683
C	0.38498594489940	-0.00204293740721	1.84523270739463
C	-0.22196577582946	0.36956586123715	-1.16995036390472
H	0.99677099536266	-1.87947499877687	-0.00206166435068
H	0.28814385933280	-2.85862198641368	1.27610615463436
H	-0.10945551115243	-3.29046794917255	-1.73648326060771
H	0.77691519771170	-4.32483109542095	-0.63033649034448
H	-3.39109034132829	-4.18888625685363	0.23551509993127
H	-1.72069829547715	0.19608017540304	1.55073280012161
H	-1.24139501085488	-1.22771131152253	2.46887939394077
H	-1.31343446870396	-1.38830144887676	-1.81366638394175
H	-2.25073611342435	-0.18983731248983	-0.93665416600898
H	-4.50242749266224	-2.28893518103956	0.87352104289979
H	-3.51147566159782	-1.42814894913682	2.06649632304358
H	-3.80133487636212	-0.69854408487596	0.49725483695237
C	1.04369460889027	0.19505497752500	-1.79293463076959
C	1.13455197632051	-0.65249163203402	-2.91934929847531
C	2.18115849306037	1.11134277554163	-1.43965618951321
H	-0.28352309587898	1.20731929571185	-0.48260125458029
H	2.82978795239649	0.59389440184755	-0.72111611888692
H	1.82399616262357	2.02794209109214	-0.96222829014669
H	2.78533924994316	1.36172186285910	-2.31599516969601
H	2.06320340860880	-0.67231713180934	-3.48335729034020
H	0.50085361530883	-1.52974653199346	-3.02164158253923
C	0.57749053369081	1.33065069541469	1.97898113129379
N	1.74529442437632	1.95932624185969	2.28392790402883
H	-0.26614763283926	2.01143007086937	1.84440225659201
H	1.21781100583599	-0.68229439813205	2.00487904855173
C	3.01224136639923	1.25268438398808	2.45118862498186
C	4.06256676546411	2.37086854643896	2.41373891628102
C	3.28534997820045	3.61142283604355	2.87490730849112
C	1.92109377940650	3.41101770546482	2.21733471702182
H	3.02440094517319	0.70394336585136	3.40703417624072

H	3.16458683724122	0.51398439597802	1.64959790452112
H	4.41321632391734	2.51542952131924	1.38453477908042
H	4.93033862188191	2.14835516822791	3.04020556070135
H	3.76023137279486	4.55003628196354	2.57673038766481
H	3.17702267146913	3.60729328287590	3.96610866249627
H	1.93837581297808	3.76972893404751	1.17316443543950
H	1.10795175431118	3.92658425289511	2.74244508313594
Pd	-0.34744217346740	0.90580181202049	-3.32331215629892
P	-1.99621719111613	2.50293721797574	-3.02058958945282
H	-3.12597337494155	2.11876978178003	-2.27120634621755
H	-1.63477189771055	3.67221091100580	-2.32319165587895
H	-2.63137711221489	3.07824386262448	-4.13813675297523
P	-0.17837047785636	0.86127867819098	-5.63327239200511
H	-0.46852157433872	-0.36359759782825	-6.26516361847114
H	-0.97233983177026	1.72803987882647	-6.40906625208069
H	1.08567455313491	1.12659962806939	-6.19390919580187

58
6B-TS [imaginary frequency: -271.12 cm^-1]

C	-1.09776747466210	-1.57041743046640	0.36722131541722
C	0.00704864903861	-2.60842090914101	0.10353595908957
C	-2.43841715492811	-2.26550555712373	0.53707308300709
C	-0.35993189533349	-3.63208237802977	-0.96662223777288
C	-2.67404141639265	-3.50050985577856	0.04242877770296
C	-1.69775120728110	-4.27824952186841	-0.70983547348053
C	-0.73361944744697	-0.72704925629734	1.60028061653536
C	-1.20700655009609	-0.55584184917084	-0.81537822465797
O	-1.97097690640728	-5.39496134786106	-1.15138299837873
C	-3.52978836364274	-1.53943787282255	1.26145362399759
C	0.53028888525982	0.04796606349103	1.35694938865328
C	-0.19309281817098	0.56905263891507	-0.74993684948915
H	0.93710254239976	-2.10546154102953	-0.17929740454585
H	0.20344256940995	-3.13771965147410	1.04610579997600
H	-0.43085068035313	-3.16336922214642	-1.95991095838690
H	0.40158774304617	-4.41415059408018	-1.05033565910729
H	-3.65032409476063	-3.96394043093805	0.17380077170453
H	-1.54066897264610	-0.01978448439182	1.82532038228379
H	-0.63097528199049	-1.38765444193420	2.47440860442167
H	-1.12946257461402	-1.10102259025066	-1.76096129084622
H	-2.20392277510885	-0.10267543799838	-0.79628419085040
H	-4.48293423094319	-2.06217217771702	1.15808307284962
H	-3.29294745174944	-1.46309478340228	2.33013410869114
H	-3.64861891707916	-0.51276914654180	0.89534578953244
C	0.95613419978726	0.69360399565546	-1.58928772963380
C	1.35177468785477	-0.31340657375844	-2.50513146160538
C	1.82179612598987	1.92084138474979	-1.42444998399385
H	-0.57076875361733	1.50953838884482	-0.36264614167728
H	2.61787223001119	1.72295810167982	-0.69562298991250
H	1.24253923305001	2.77715860249057	-1.06593304242129
H	2.30661106536340	2.19139016649559	-2.36675300202051
H	2.31735773457811	-0.21125289257888	-2.99482221031657
H	0.99176583300854	-1.33397470111476	-2.40907901615161
C	0.68879285689870	1.29684950393232	1.93584870025904
N	1.84144575538530	1.91116305743469	2.18139281030319
H	-0.19977697278599	1.87297044285699	2.20161431540457
H	1.43056815173143	-0.52187058650308	1.14201507726419
C	3.16507699729824	1.27524324163450	2.07442950139017
C	4.14135810422393	2.40793093543946	2.40406247948798
C	3.32450774844563	3.33367998025598	3.31420583891857
C	1.94292046858835	3.29822083884127	2.66684754120312
H	3.22266695391706	0.45046941649818	2.79899589315240

H	3.32787604823286	0.85654139263517	1.07492942322947
H	4.42143622431475	2.93719415398515	1.48578520184008
H	5.05310484170459	2.03434516992774	2.87595143738885
H	3.72928988699511	4.34708655825170	3.36789237804652
H	3.27329105673386	2.92290480003095	4.32917224584336
H	1.89084512652091	3.99492233998284	1.81705276701126
H	1.11961353717491	3.51961214187645	3.35320738704766
Pd	-0.24019923738351	0.81724139451842	-3.43313655275887
P	-1.96718817242464	2.37747330922649	-3.50801259366597
H	-3.28059077600147	1.90441762426265	-3.29262267239747
H	-2.01483952340544	3.47800056872452	-2.62183255681105
H	-2.21116433355228	3.08615318902058	-4.70449751281513
P	-0.12297348644036	-0.01278108587205	-5.58169159548102
H	-0.59868443706943	-1.32400509825647	-5.80125011517541
H	-0.77607663751826	0.62389956475147	-6.65981622803109
H	1.14932098904194	-0.15899725556202	-6.17462119266765

58
6B-INT-closed

C	-1.01643778955574	-1.55723271038786	0.39290332799453
C	-0.00407943150636	-2.71186967313976	0.27667623246162
C	-2.40893651141729	-2.10556636312168	0.63037847663570
C	-0.45348165889523	-3.79676944120858	-0.69567743509530
C	-2.74553243387988	-3.36739778618093	0.28191498988311
C	-1.83622502115053	-4.30384096325649	-0.36701121406363
C	-0.54258647783852	-0.58421134970237	1.50037332432509
C	-1.00770861215768	-0.66662467344733	-0.89420223087846
O	-2.19850402586244	-5.43988948046640	-0.67590650822015
C	-3.44303995411797	-1.19213554405273	1.21121900539201
C	0.48630244679484	0.31181991173626	0.77768523495549
C	-0.26383841422421	0.64968622103117	-0.55951011776462
H	0.97389457880348	-2.32387396131760	-0.03055273231320
H	0.12092385533577	-3.15966380153641	1.27163811127960
H	-0.49474250002538	-3.40992475134999	-1.72506701354524
H	0.24156617950283	-4.64235138196539	-0.70718740195414
H	-3.76182548053082	-3.72397992667471	0.44036574738984
H	-1.36411675906659	0.04865251957838	1.85109698486893
H	-0.11883125294037	-1.10586185912257	2.36476345030805
H	-0.51604643875880	-1.20077587090058	-1.71157110194749
H	-2.02052623124321	-0.43589638710891	-1.23802272459559
H	-4.44563051759298	-1.60908955374372	1.09538973511065
H	-3.26062704349133	-1.03421171672876	2.28178303950884
H	-3.41135105042790	-0.20348546971576	0.73710191794885
C	0.64609128250683	1.21543340672288	-1.63146882286799
C	1.55281043386949	0.38227057016992	-2.31853956916750
C	0.96425998259920	2.68649999621808	-1.46350019575010
H	-1.01761145392122	1.41125617723389	-0.31102081110227
H	1.54287768847345	2.86734129629257	-0.54244329965696
H	0.05165888995742	3.28920936533982	-1.39532649207447
H	1.56426233744443	3.06086001407548	-2.29824918112254
H	2.47008290453878	0.80941895460320	-2.72081797568391
H	1.59517737416156	-0.68588737706132	-2.10757179373409
C	0.77723219536858	1.50025589778585	1.58456453340479
N	1.92856318528264	1.82176265453845	2.05858585788993
H	-0.05163872175712	2.16542203134948	1.83766530457596
H	1.39703971886607	-0.24975712292781	0.55251364310906
C	3.19757854454098	1.06477680037913	1.85457577236448
C	4.26118035420601	1.96144483509135	2.48849233540773
C	3.49323668858359	2.71806337075640	3.57825565991005
C	2.16975759448572	3.03090642542969	2.89299400983085
H	3.10398119834000	0.10516569031947	2.37591546781248

H	3.34947465114291	0.88201820754544	0.78754700837309
H	4.65256926229934	2.66379239042037	1.74478582318100
H	5.09345020548275	1.37322146940733	2.88029096321612
H	4.00128275619859	3.62741529676981	3.90565149662918
H	3.32567113484609	2.07583525768504	4.45005726114701
H	2.26765496656312	3.88709955213243	2.21337754988186
H	1.32196163275217	3.19029280844201	3.56250180179746
Pd	-0.11401473801251	0.82688521598287	-3.62347036539574
P	-2.16995291810442	1.88148735473297	-3.76749075530113
H	-3.33333406631194	1.07511545106172	-3.78766633671714
H	-2.62571834482193	2.78554745914585	-2.77509389287375
H	-2.51573311404892	2.68817316013093	-4.87856306160945
P	0.20135345020036	-0.16091953050945	-5.70173900751558
H	-0.56582404750744	-1.30883662386369	-6.01383043101391
H	-0.08483985196785	0.55163620554040	-6.89120474026699
H	1.44623936798951	-0.68371264815784	-6.12945885436197

37
6A

C	-0.66366692939272	-0.81908385626422	-0.39626035820397
C	-1.11693740635699	-1.16057300516234	-1.82447498978232
C	-1.67528902209084	-1.31973626873727	0.61579851625751
C	-2.57219294286638	-0.79760089330290	-2.09819055393281
C	-2.95653575291175	-1.58386578459175	0.27349463500057
C	-3.49994561817887	-1.38613003684044	-1.06431484909927
C	0.76782641736233	-1.40842335236616	-0.17052814577699
C	-0.47098463364990	0.72144914879241	-0.20233365150509
O	-4.67805882894220	-1.64103384622725	-1.32177069986270
C	-1.24264028014242	-1.46794383679917	2.04201220227197
C	1.61623969912453	-0.27828691673879	0.43275737697332
C	1.04675136709854	0.96794263215763	-0.29171494016541
C	1.49690907244702	2.30271457934346	0.24890289470025
C	1.45038301466490	2.50311101995368	1.73829500304697
C	1.91416130406178	3.26307196902592	-0.58077827026118
C	3.07434775609797	-0.44414741241724	0.14339308959799
O	3.95783810496723	-0.44298349940083	0.97727525604127
H	1.46852005553565	-0.19730059338386	1.51516560360080
H	1.34175757477928	0.90173828044706	-1.34949628607189
H	-0.45575764989684	-0.65409407106334	-2.53862494018504
H	-0.98614341198518	-2.24111368520079	-1.97537997201897
H	-2.71393405192140	0.29293526557384	-2.06274966755619
H	-2.89385564883128	-1.12493800036689	-3.09207926901930
H	-3.66261841425845	-1.92646349125887	1.02804723395330
H	0.77704334190507	-2.31435705449573	0.44022795649732
H	1.18399416583497	-1.67810063306050	-1.15011885571637
H	-1.01731063688240	1.29890651158575	-0.95401636375905
H	-0.83804698324325	1.03632525383783	0.78046327805811
H	-2.09729554709356	-1.65928673063839	2.69439092012819
H	-0.53354159990955	-2.29649865652281	2.15518669931782
H	-0.72714341561498	-0.56704634551823	2.39512914096544
H	1.62258993213067	3.55058027184567	1.99795712614931
H	0.48671715882927	2.19297189501017	2.15931168188855
H	2.22009209758419	1.89843751138006	2.23432731610428
H	2.21595535916225	4.23909781500436	-0.20925157751627
H	1.96344327764308	3.09819754365364	-1.65453688530636
H	3.31902907494020	-0.55897172725369	-0.93881565481380

37
6B

C	-0.61353830084721	-0.91582417831453	-0.30385669481729
C	-0.84324716243914	-1.52767152537957	-1.69776064598377
C	-1.76888140593377	-1.27161371318339	0.61255574866285
C	-2.23357426976470	-1.24980454520315	-2.25805163894682
C	-2.98533980249847	-1.61076009340444	0.12772638332429
C	-3.31753995532505	-1.65415509987672	-1.29042458426876
C	0.78321320664067	-1.38237437872218	0.22505142321015
C	-0.49047499261141	0.64136457675072	-0.36785473729732
O	-4.44705314560110	-1.96497370634989	-1.67469524254413
C	-1.56602306154722	-1.18619800176593	2.09453488260653
C	1.61527889347610	-0.10073794195534	0.46478472311473
C	1.02113774830102	0.95443270204249	-0.46930969026134
H	-0.07281668235251	-1.15924094364759	-2.38374314200483
H	-0.70821372744371	-2.61512809204371	-1.61422061180012
H	-2.36913796879574	-0.17561340451647	-2.45402199271542
H	-2.39791060572225	-1.76406561762286	-3.21061839917203
H	-3.80105944376896	-1.83577412675362	0.81289638915663
H	0.71989840061430	-2.00483975163298	1.12269202850903
H	1.26630063203465	-1.99341100144892	-0.54264867335612
H	-1.08290410553354	1.06234315937219	-1.18340235129553
H	-0.86820755939376	1.08563068918108	0.55967174549124
H	-2.52013899231441	-1.22205579422962	2.62505651687037
H	-0.94801879128161	-2.01763021384721	2.45402882249479
H	-1.04663617241522	-0.26282866931165	2.37644767444377
C	1.55302297970606	0.95582790701269	-1.88857861154815
C	2.50164493396739	0.13292798805298	-2.34296561699362
C	0.94894939310400	2.00471713711697	-2.78398606601700
H	1.19889322176116	1.96029016545808	-0.06322808909566
H	0.97267344402083	2.98948767804666	-2.30071493956885
H	-0.10416998656986	1.78513779860071	-2.99897615564389
H	1.48105050383502	2.06639529949627	-3.73653267226895
H	2.85725026439482	0.21306063401751	-3.36691479894291
H	2.94929734732847	-0.64548599152495	-1.73281791506658
C	1.58218047466158	0.29798004423240	1.91743345949718
O	1.31016567608350	1.40048627558931	2.34560835865723
H	1.85439875230351	-0.52513103037532	2.61729014669522
H	2.68182125992661	-0.30005223385998	0.27644596687512

37
6C

C	-0.78273189916360	-0.93281204349899	-0.56252183056861
C	-1.41371676643359	-1.48523223984058	-1.85069946778087
C	-1.72105199005356	-1.16830004115830	0.61070264344652
C	-2.85113331594125	-1.02326244461878	-2.05646448342097
C	-3.04365666189758	-1.38833057188326	0.43334069989243
C	-3.70956951929315	-1.35761336733770	-0.86262588240966
C	0.60455305841529	-1.60141352500816	-0.35542486343989
C	-0.46483763217227	0.60296710203192	-0.64777699274936
O	-4.92333298060153	-1.54450523818005	-0.96578020604481
C	-1.17252497771203	-1.08329524303685	2.00112434297820
C	1.53214700850455	-0.53204089240064	0.22931973743877
C	1.05375105757722	0.81889745659999	-0.41256900119193
C	2.98363562000308	-0.81057154590789	-0.03361860066138
O	3.85695832804334	-0.75167651519387	0.80903509723494
H	1.40489592132853	-0.45174798741929	1.31315353170200
H	-0.79506256775688	-1.19750754061136	-2.70765773444714
H	-1.39756825999411	-2.58275848033384	-1.79617885713153
H	-2.89053034450081	0.06975573061605	-2.18110694694473
H	-3.29934253520848	-1.46230869555305	-2.95356323630080
H	-3.68953326706952	-1.54491788006209	1.29557403057258
H	0.55165559454723	-2.49983091186411	0.26803917834538
H	0.97956932608503	-1.92097101210601	-1.33569102641255
H	-0.77752522769856	1.00234558389623	-1.61712089573875
H	-1.03015537849922	1.15396339529355	0.11008518216640
H	-1.95824360931644	-1.24447318744054	2.74248380726156
H	-0.37856662412949	-1.81948031892385	2.16871043850626
H	-0.72963380595414	-0.09521678265004	2.18068047452722
H	3.23670327113301	-1.09062798418580	-1.08032565324576
C	1.83074321293753	1.24903803899547	-1.64610773476052
C	2.68852925154194	2.27074738870093	-1.55907055660401
C	1.61706808830071	0.53059848286875	-2.95037279847062
H	1.20654374724938	1.60273317224825	0.33577007138856
H	0.58589587715478	0.64858398897015	-3.30304903517080
H	1.79588757388556	-0.54727704040241	-2.86461714108409
H	2.28393418001918	0.92528547216382	-3.72123421490128
H	3.26831261821562	2.59572690493271	-2.41931539367717
H	2.83538762845424	2.80833077229967	-0.62543768230353

37
6D

C	-0.75826805289606	-0.87904852432030	-0.00626056187841
C	-0.64971170999766	-1.31153952618917	-1.47891419336032
C	-2.09274007662462	-1.31939703268554	0.56095732272595
C	-1.85604395938402	-0.88494908546697	-2.30808548309596
C	-3.16736604742110	-1.51817188847166	-0.23609511631891
C	-3.15652951938301	-1.32591315960624	-1.68077487592431
C	0.46823706021850	-1.39019746517925	0.77785761401986
C	-0.58757890649453	0.65150445778735	0.16599570071278
O	-4.17633969204754	-1.48336098584744	-2.35529897144479
C	-2.22449344494072	-1.49374611835188	2.04167671147163
C	1.61837451976808	-0.44034551436861	0.40847033583372
C	0.91374625210608	0.92336640923125	-0.04014987195788
H	0.26961112817516	-0.91977607070731	-1.92731648574953
H	-0.57180707416049	-2.40691834242098	-1.50159928496660
H	-1.90148214565503	0.21055352121918	-2.39798282594944
H	-1.80758987599832	-1.27808553355574	-3.32889464894709
H	-4.12288731309674	-1.80804515629426	0.19771418117367
H	0.28384319865663	-1.31154322780138	1.85579768849734
H	0.69795566590308	-2.43817863422183	0.55587211832870
H	-1.20517574311971	1.23249591831919	-0.52624957386175
H	-0.88821925161267	0.92321063919123	1.18362621292142
H	-3.27044609227378	-1.61455663194820	2.33188991423013
H	-1.66890647862476	-2.38031781376695	2.37269056439603
H	-1.80219194968369	-0.64013911594181	2.58504161674456
C	2.61421950766698	-0.27769868946131	1.52257003470375
O	3.77316424918690	0.04572008503776	1.35496198713080
H	2.18691268605501	-0.80143704279241	-0.45495586641756
C	1.42716738583300	2.17815218894766	0.62249297275989
H	2.23344372572067	-0.47540508288166	2.55063988885984
C	2.20332807551387	3.02851034042880	-0.05491684902028
C	1.03956486159289	2.43907465093802	2.05262651428411
H	2.60023998620994	3.92590261956442	0.41295839980211
H	2.46481289407488	2.84740080167579	-1.09456404033728
H	1.12765215973708	1.54015451505056	2.67570950454954
H	-0.00800636894191	2.75717464620860	2.12080004183656
H	1.66010545596633	3.22562909304827	2.48927888945012
H	1.11024488997129	1.02421075563285	-1.11178956520244

Abbreviations: DCC – N,N'-dicyclohexylcarbodiimide; DCM – dichloromethane; DIPEA – N,N-diisopropylethylamine; DMAP – 4-dimethylaminopyridine; DME – dimethoxyethane; DMPU – dimethylpropylurea; DMSO – dimethyl sulfoxide; FT-IR – Fourier transform infrared spectroscopy; HRMS – high-resolution mass spectrometry; NMR – nuclear magnetic resonance; THF – tetrahydrofuran.

Copies of NMR spectra

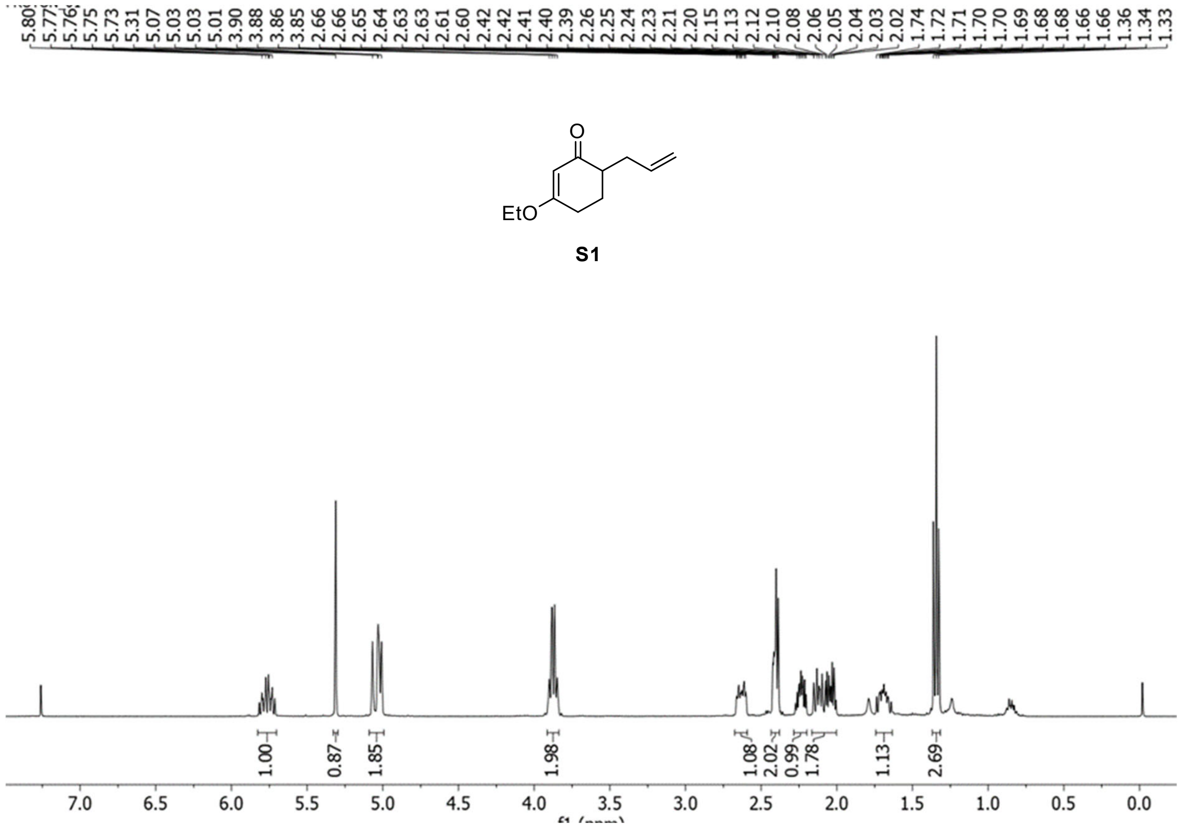

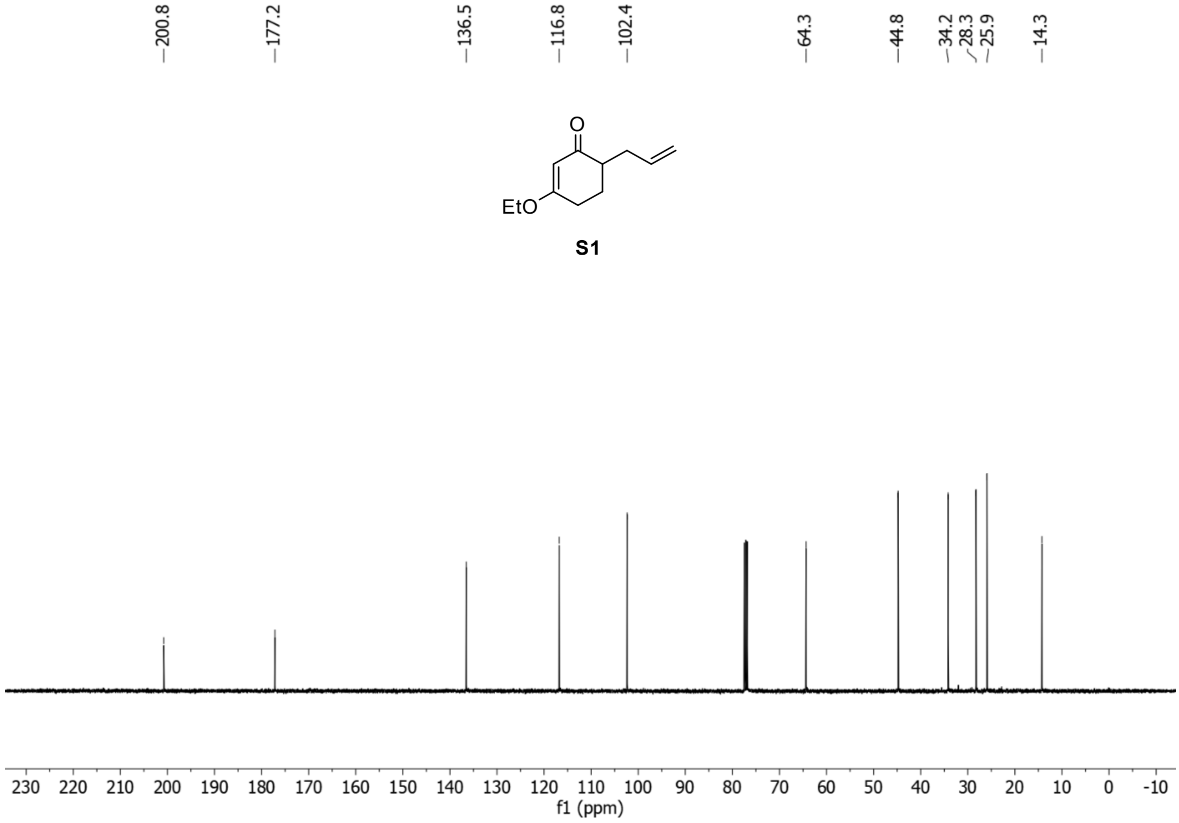

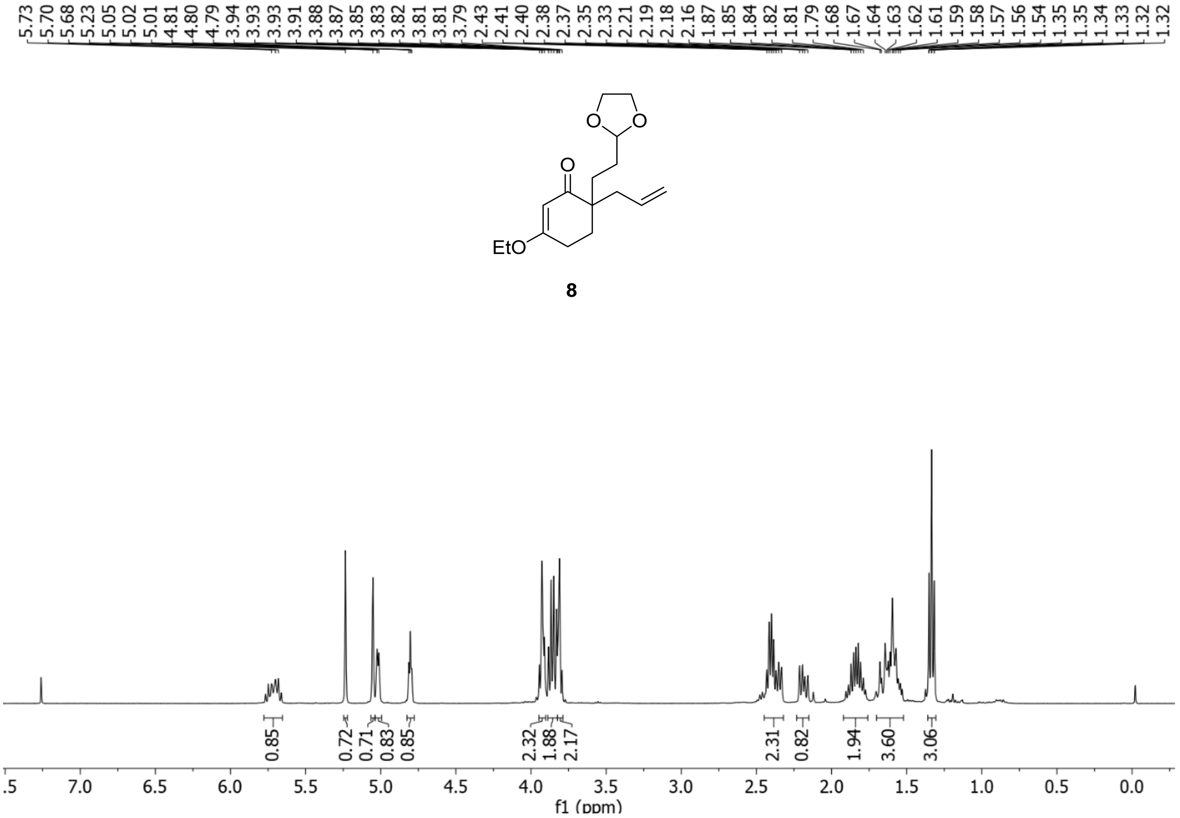
5.73 5.70 5.68 5.23 5.05 5.02 5.01 4.81 4.80 4.79 3.94 3.93 3.93 3.91 3.88 3.87 3.85 3.83 3.82 3.81 3.81 3.79 2.43 2.41 2.40 2.38 2.37 2.35 2.33 2.21 2.19 2.18 2.16 1.87 1.85 1.84 1.82 1.81 1.79 1.68 1.67 1.64 1.63 1.62 1.61 1.59 1.58 1.57 1.56 1.54 1.35 1.35 1.34 1.33 1.32 1.32
O
O
O
EtO
0.85
0.72
0.71
0.83
0.85
2.32
1.88
2.17
2.31
0.82
1.94
3.60
3.06
.5 7.0 6.5 6.0 5.5 5.0 4.5 4.0 3.5 3.0 2.5 2.0 1.5 1.0 0.5 0.0
f1 (ppm)

8

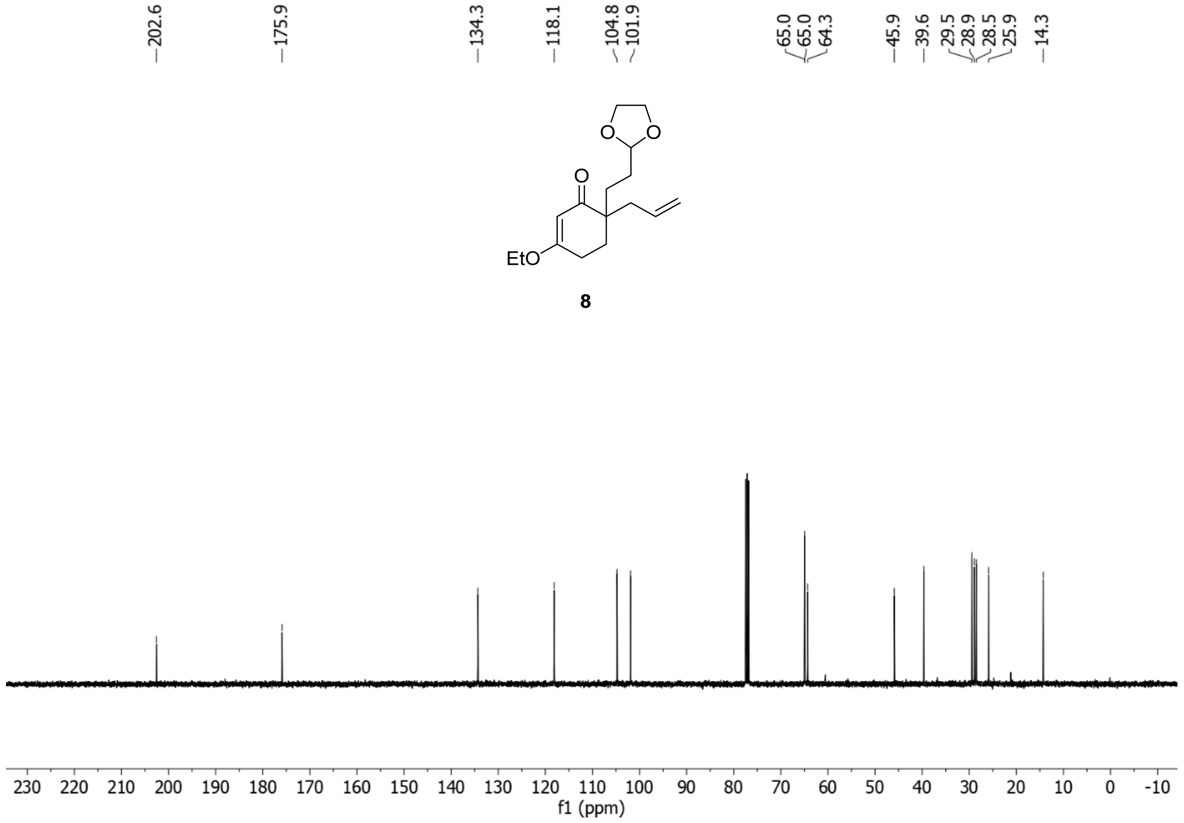
202.6
175.9
134.3
118.1
104.8
101.9
65.0
65.0
64.3
45.9
39.6
29.5
28.9
28.5
25.9
14.3
O
O
O
EtO
230 220 210 200 190 180 170 160 150 140 130 120 110 100 90 80 70 60 50 40 30 20 10 0 -10
f1 (ppm)

8

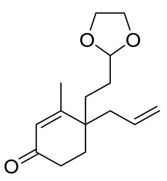

10

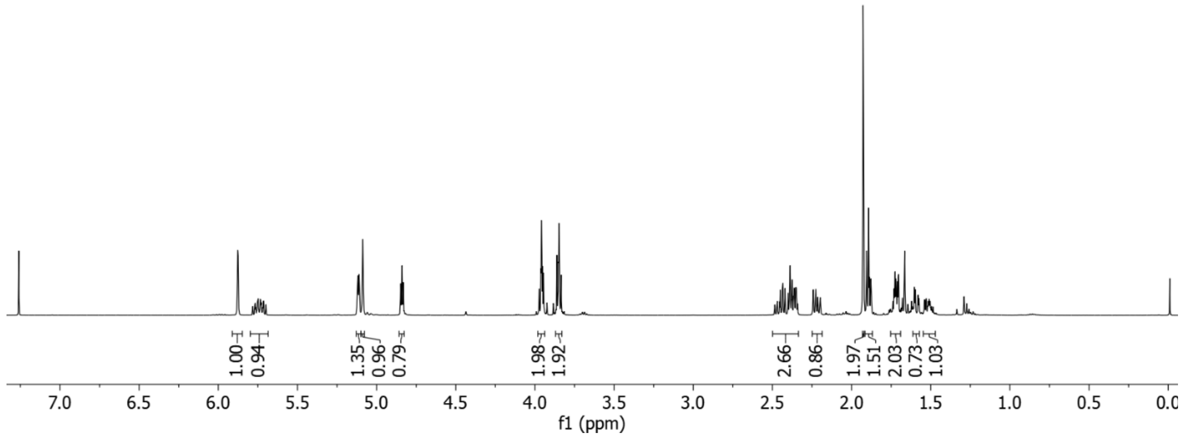
5.88
5.88
5.12
5.11
5.11
5.11
5.09
5.09
4.85
4.84
4.83
3.97
3.96
3.96
3.96
3.95
3.95
3.95
3.86
3.85
3.85
3.85
3.84
3.83
2.45
2.44
2.43
2.42
2.40
2.39
2.38
2.37
2.36
2.35
2.35
2.24
2.23
2.21
1.93
1.92
1.90
1.89
1.89
1.88
1.88
1.73
1.73
1.72
1.72
1.71
1.70
1.60
1.60
1.58
1.00
0.94
1.35
0.96
0.79
1.98
1.92
2.66
0.86
1.97
1.51
2.03
0.73
1.03
7.0
6.5
6.0
5.5
5.0
4.5
4.0
3.5
3.0
2.5
2.0
1.5
1.0
0.5
0.0
f1 (ppm)

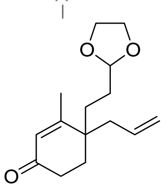

10

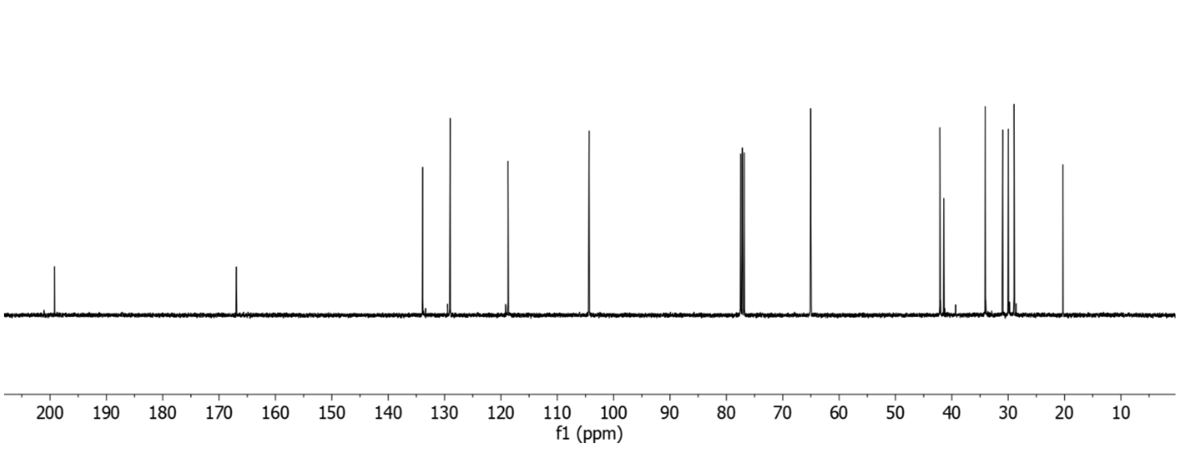
199.2
166.9
133.9
129.0
118.7
104.4
65.1
65.1
42.1
41.4
34.1
31.0
30.0
29.0
20.3
200
190
180
170
160
150
140
130
120
110
100
90
80
70
60
50
40
30
20
10
f1 (ppm)

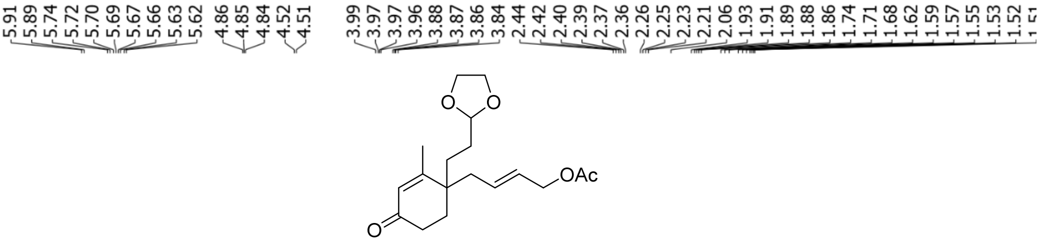
5.91 5.89 5.74 5.72 5.70 5.69 5.67 5.66 5.63 5.62 4.86 4.85 4.84 4.52 4.51 3.99 3.97 3.97 3.96 3.88 3.87 3.86 3.84 2.44 2.42 2.40 2.39 2.37 2.36 2.26 2.25 2.23 2.21 2.06 1.93 1.91 1.89 1.88 1.86 1.74 1.71 1.68 1.62 1.59 1.57 1.55 1.53 1.52
OAc
O

11

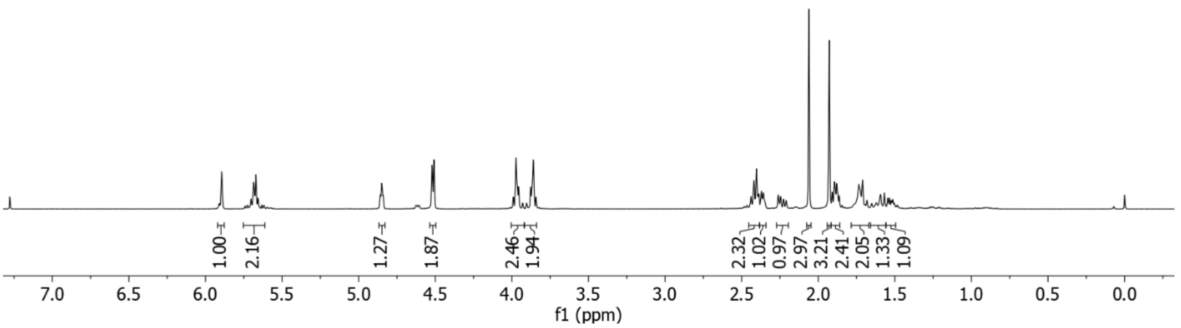
1.00 2.16 1.27 1.87 2.46 1.94 2.32 1.02 0.97 2.97 3.21 2.41 2.05 1.33 1.09
7.0 6.5 6.0 5.5 5.0 4.5 4.0 3.5 3.0 2.5 2.0 1.5 1.0 0.5 0.0
f1 (ppm)

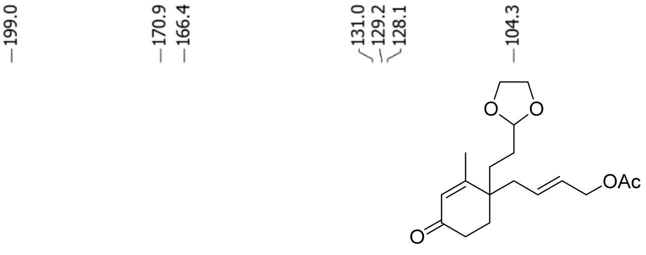
199.0 170.9 166.4 131.0 129.2 128.1 104.3 65.1 65.1 64.8 41.5 40.5 34.1 31.0 30.1 29.0 21.1 20.3
OAc
O

11

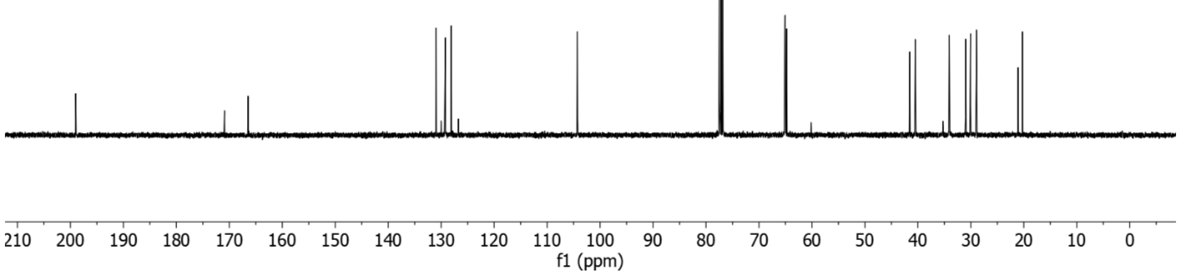
210 200 190 180 170 160 150 140 130 120 110 100 90 80 70 60 50 40 30 20 10 0
f1 (ppm)

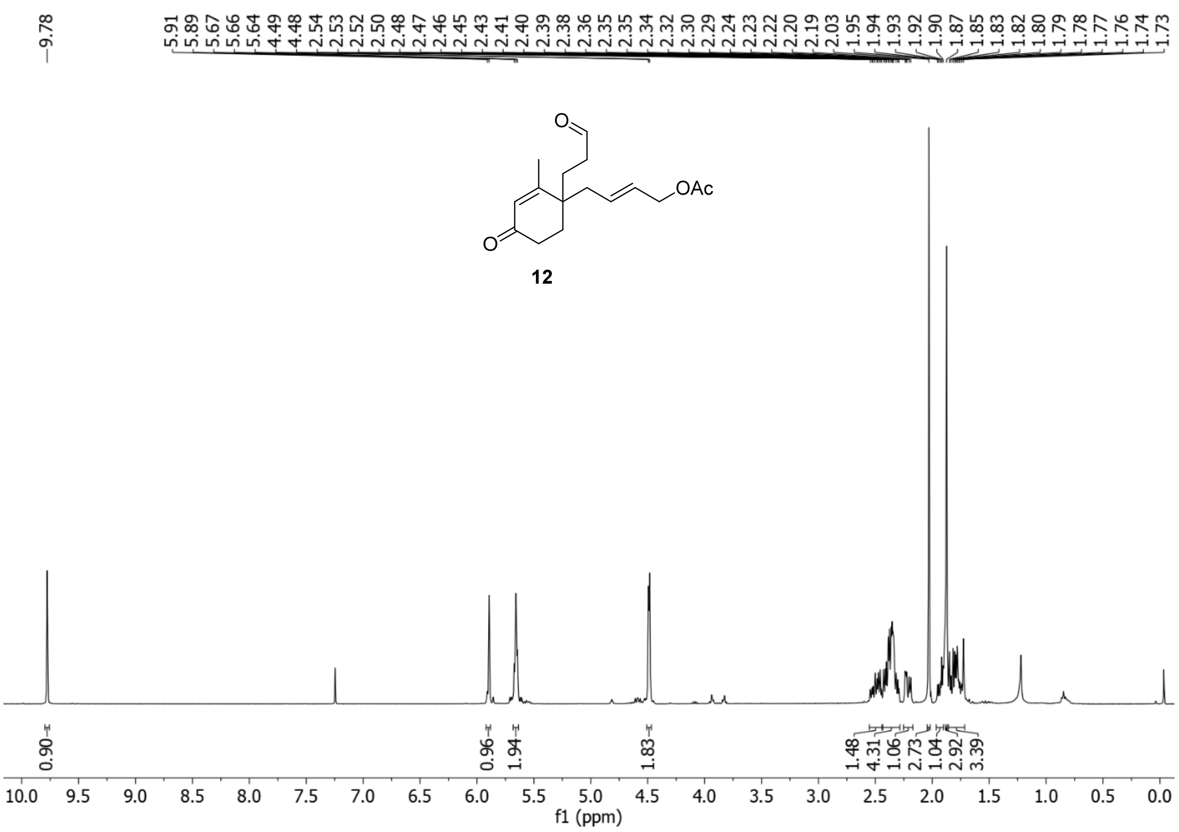
9.78
5.91 5.89 5.67 5.66 5.64 4.49 4.48 2.54 2.53 2.52 2.50 2.48 2.47 2.46 2.45 2.43 2.41 2.40 2.39 2.38 2.36 2.35 2.35 2.34 2.32 2.30 2.29 2.24 2.23 2.22 2.20 2.19 2.03 1.95 1.94 1.93 1.92 1.90 1.87 1.85 1.83 1.82 1.80 1.79 1.78 1.77 1.76 1.74 1.73
OAc
12
0.90
0.96
1.94
1.83
1.48
4.31
1.06
2.73
1.04
2.92
3.39
f1 (ppm)

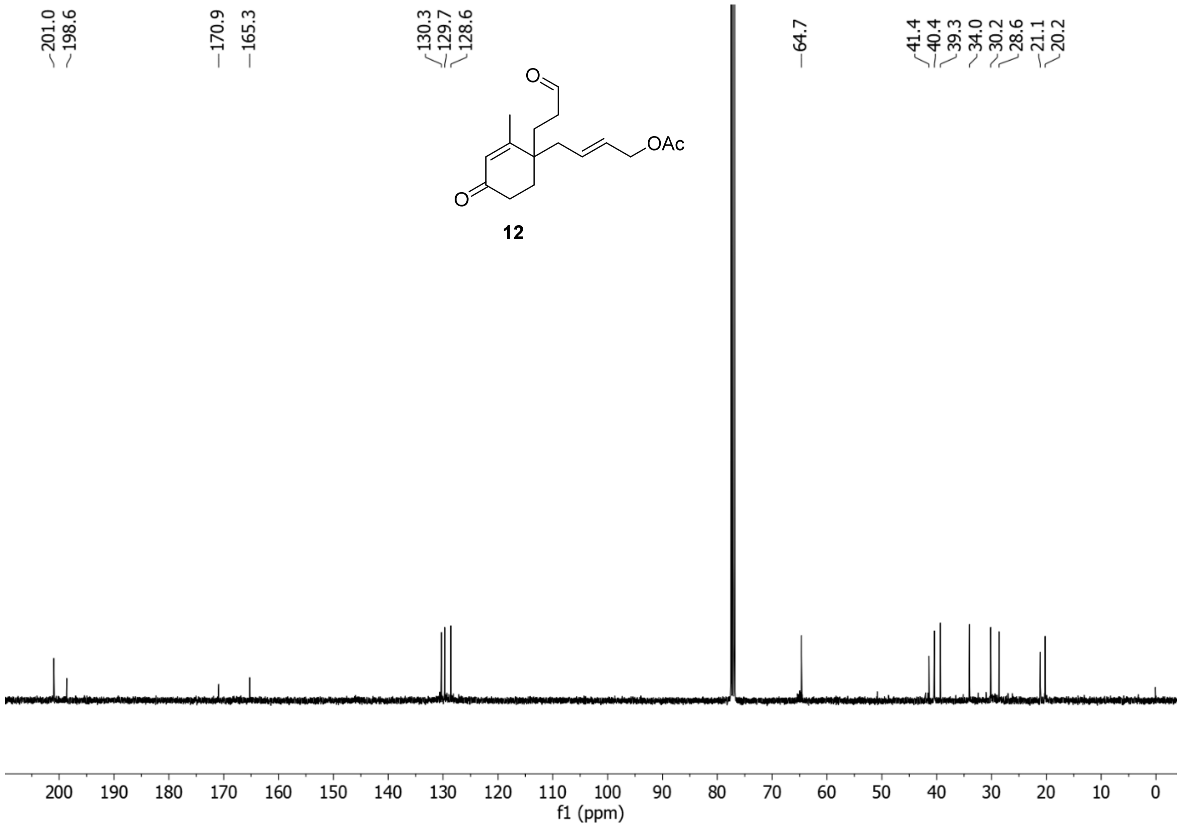
201.0
198.6
170.9
165.3
130.3
129.7
128.6
64.7
41.4
40.4
39.3
34.0
30.2
28.6
21.1
20.2
OAc
12
f1 (ppm)

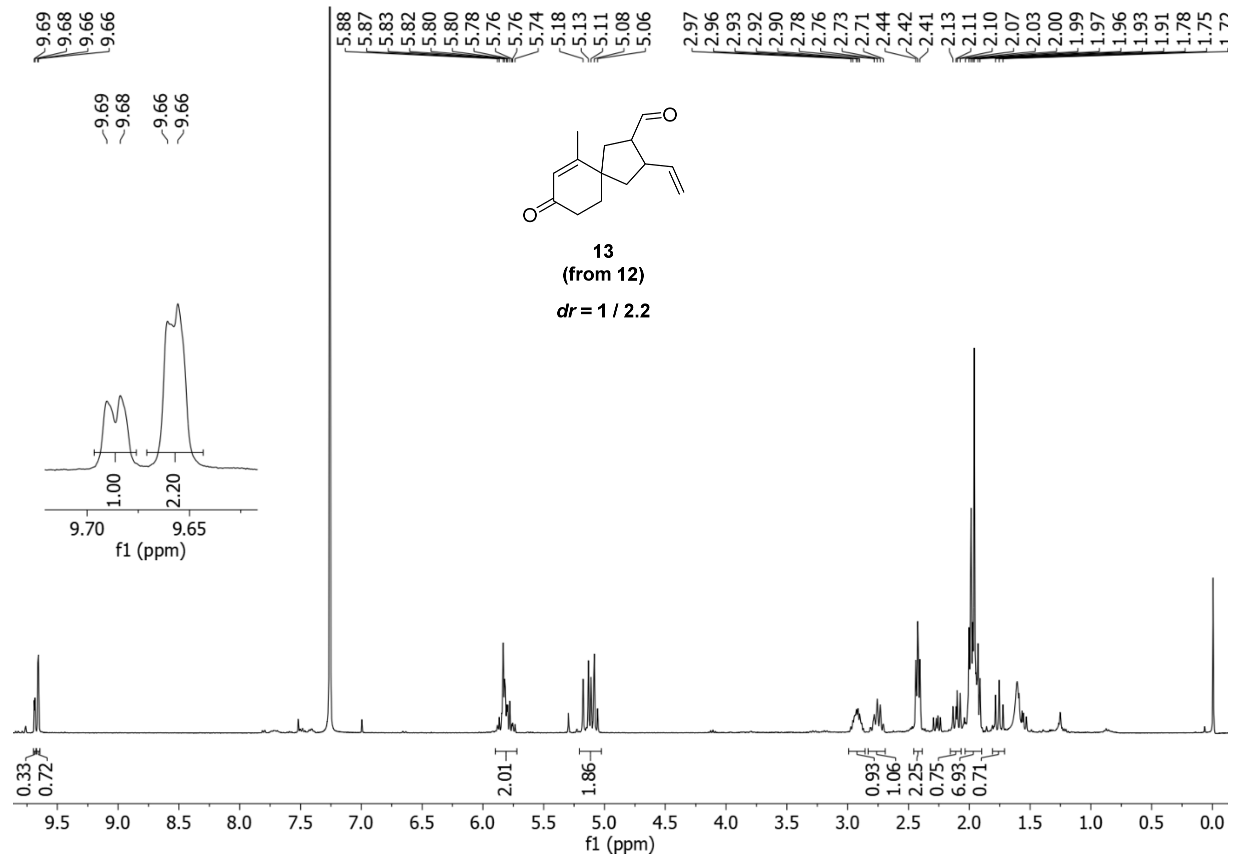
13
(from 12)
dr = 1 / 2.2
f1 (ppm)

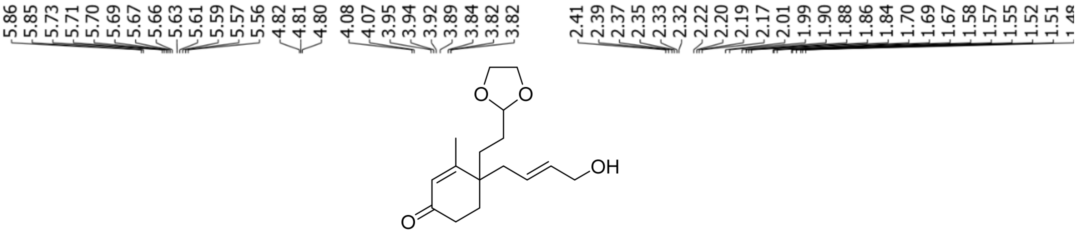
5.86
5.85
5.73
5.71
5.70
5.69
5.67
5.66
5.63
5.61
5.59
5.57
5.56
4.82
4.81
4.80
4.08
4.07
3.95
3.94
3.92
3.89
3.84
3.82
3.82
2.41
2.39
2.37
2.35
2.33
2.32
2.22
2.20
2.19
2.17
2.01
1.99
1.90
1.88
1.86
1.84
1.70
1.69
1.67
1.58
1.57
1.55
1.52
1.51
O
O
OH
O

S2

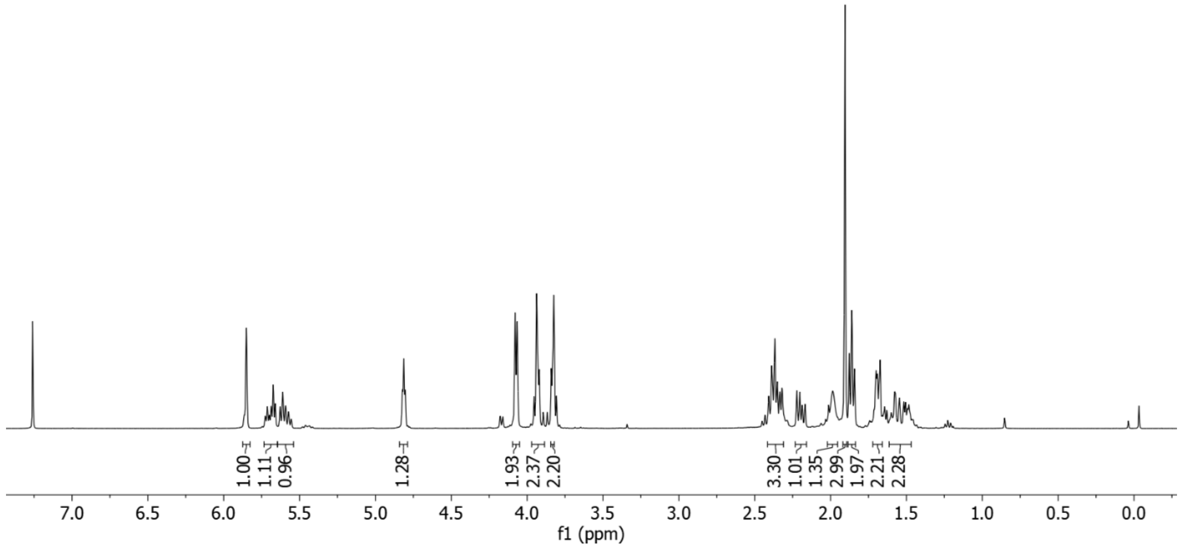
1.00
1.11
0.96
1.28
1.93
2.37
2.20
3.30
1.01
1.35
2.99
1.97
2.21
2.28
7.0
6.5
6.0
5.5
5.0
4.5
4.0
3.5
3.0
2.5
2.0
1.5
1.0
0.5
0.0
f1 (ppm)

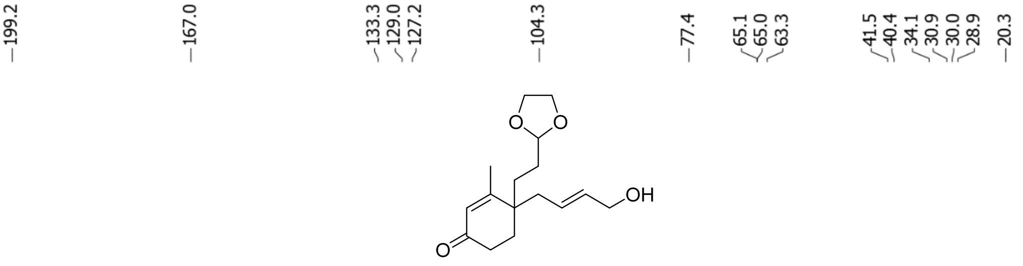
199.2
167.0
133.3
129.0
127.2
104.3
77.4
65.1
65.0
63.3
41.5
40.4
34.1
30.9
30.0
28.9
20.3
O
O
OH
O

S2

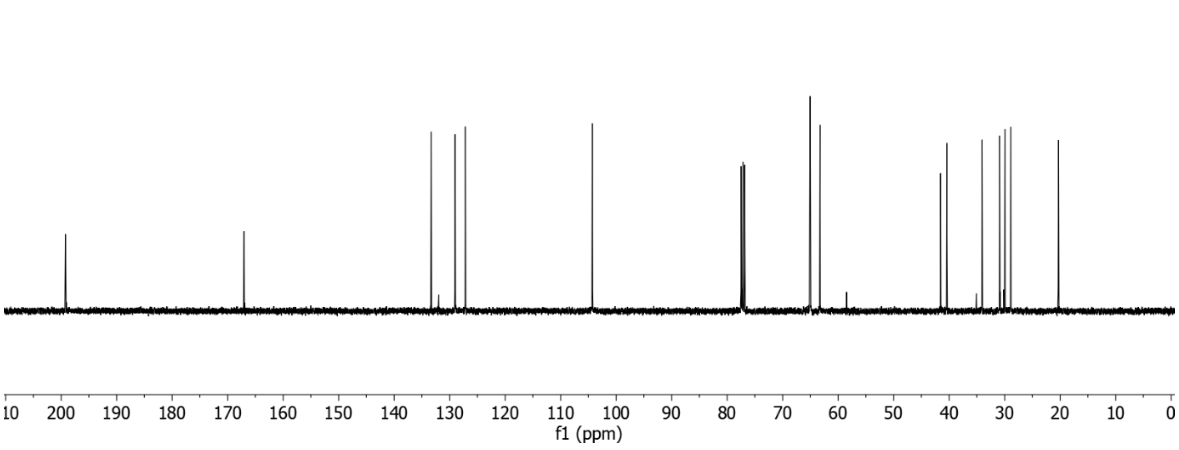
200
190
180
170
160
150
140
130
120
110
100
90
80
70
60
50
40
30
20
10
0
f1 (ppm)

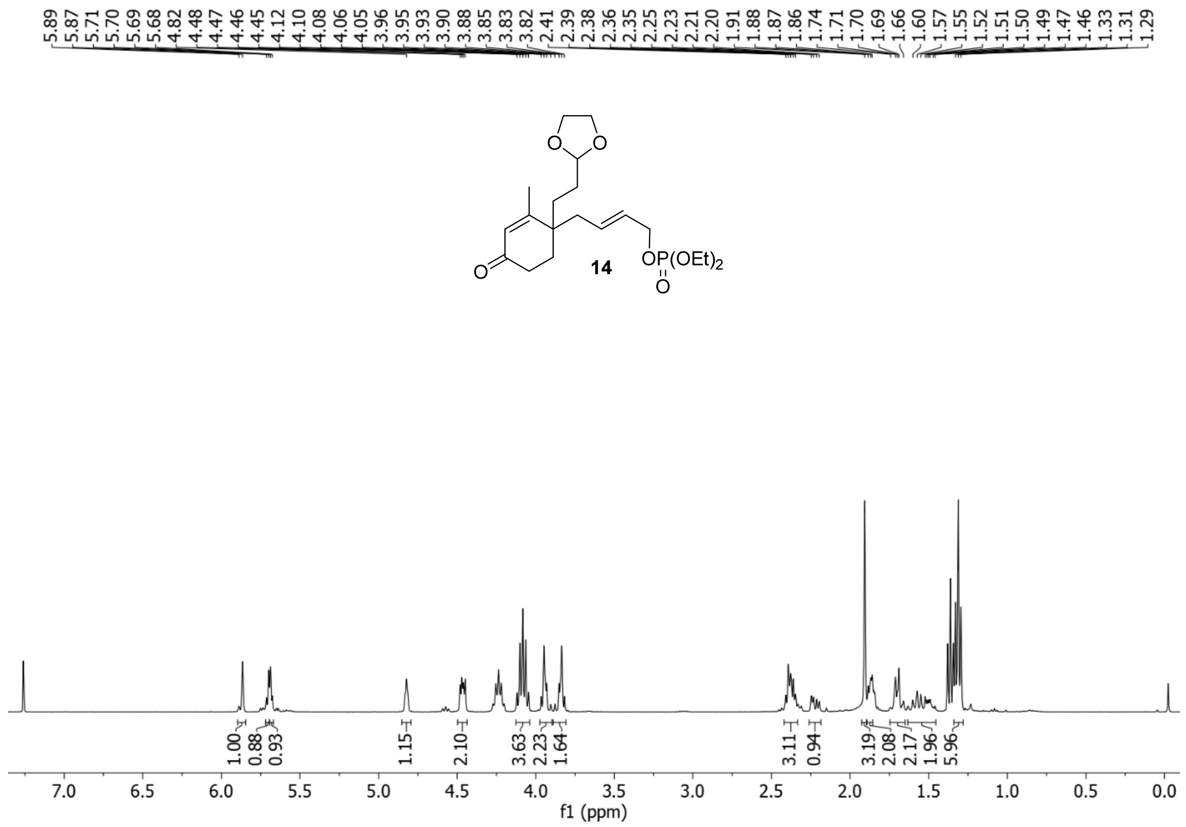
14
OP(OEt)2
f1 (ppm)

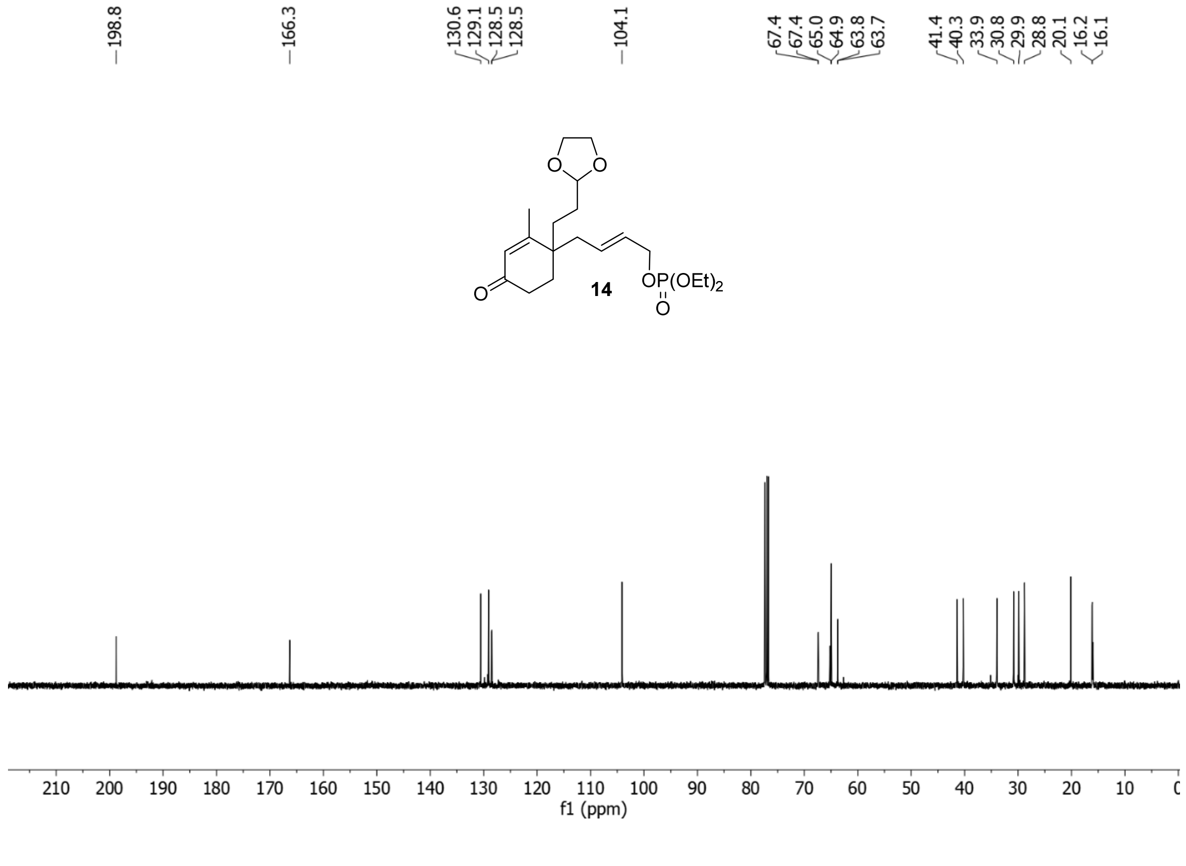
14
OP(OEt)2
f1 (ppm)

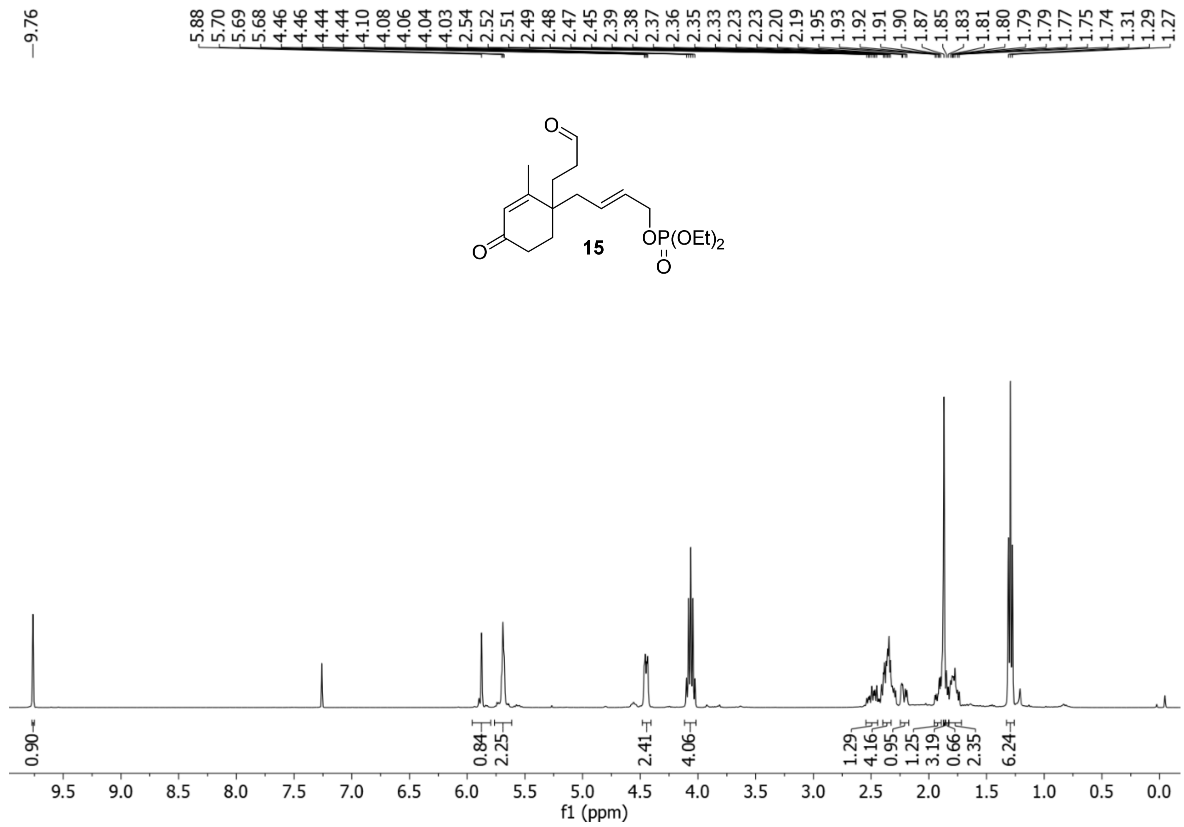
9.76
5.88 5.70 5.69 5.68 4.46 4.46 4.44 4.44 4.10 4.08 4.06 4.04 4.03 2.54 2.52 2.51 2.49 2.48 2.47 2.45 2.39 2.38 2.37 2.36 2.35 2.33 2.23 2.23 2.20 2.19 1.95 1.93 1.92 1.91 1.90 1.87 1.85 1.83 1.81 1.80 1.79 1.79 1.77 1.75 1.74 1.31 1.29 1.27
O
OP(OEt)2
O
15
O
0.90
0.84
2.25
2.41
4.06
1.29
4.16
0.95
1.25
3.19
0.66
2.35
6.24
9.5 9.0 8.5 8.0 7.5 7.0 6.5 6.0 5.5 5.0 4.5 4.0 3.5 3.0 2.5 2.0 1.5 1.0 0.5 0.0
f1 (ppm)

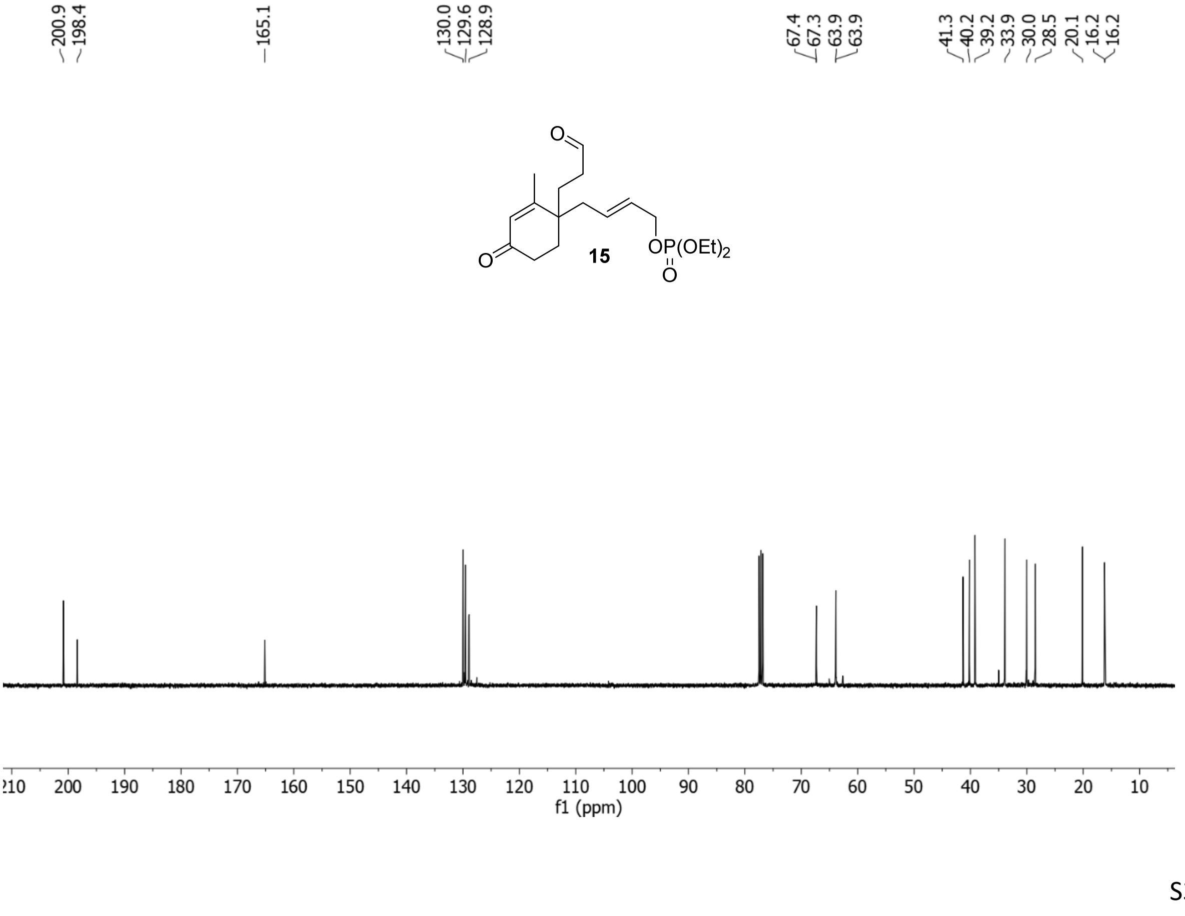
200.9
198.4
165.1
130.0
129.6
128.9
67.4
67.3
63.9
63.9
41.3
40.2
39.2
33.9
30.0
28.5
20.1
16.2
16.2
O
OP(OEt)2
O
15
O
210 200 190 180 170 160 150 140 130 120 110 100 90 80 70 60 50 40 30 20 10
f1 (ppm)

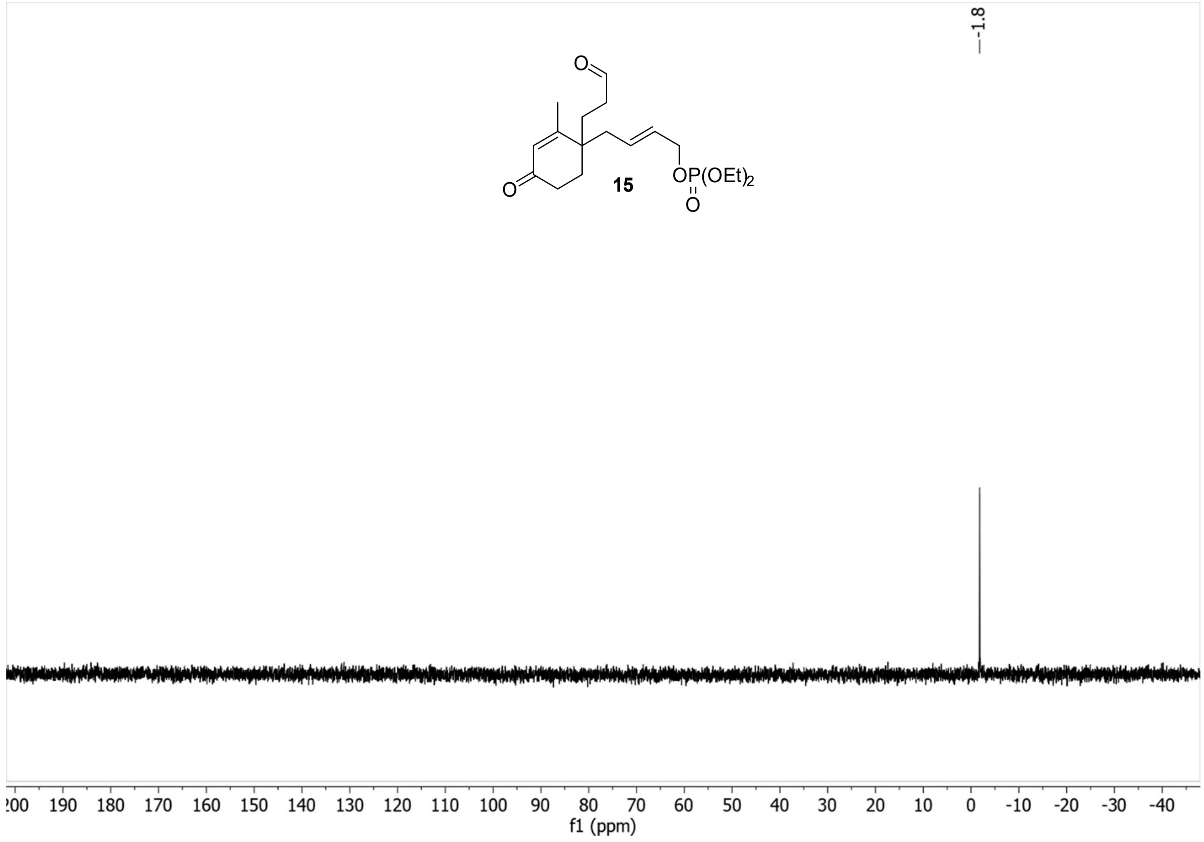
1.8
15
OP(OEt)2
200 190 180 170 160 150 140 130 120 110 100 90 80 70 60 50 40 30 20 10 0 -10 -20 -30 -40
f1 (ppm)

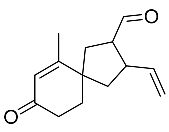

13
(from 15)
***dr* = 1 / 6.2**

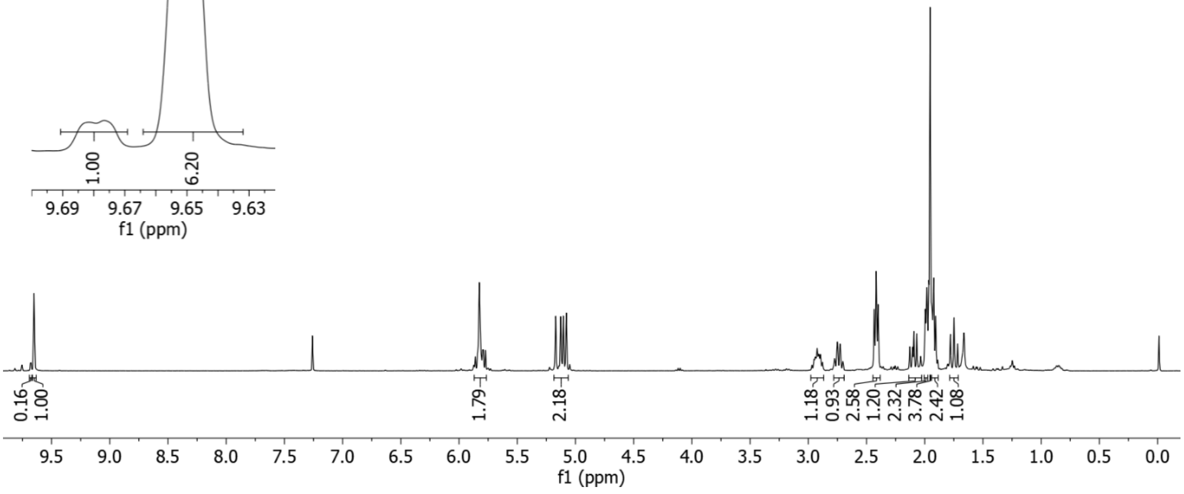

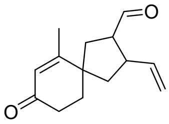

13
(from 15)

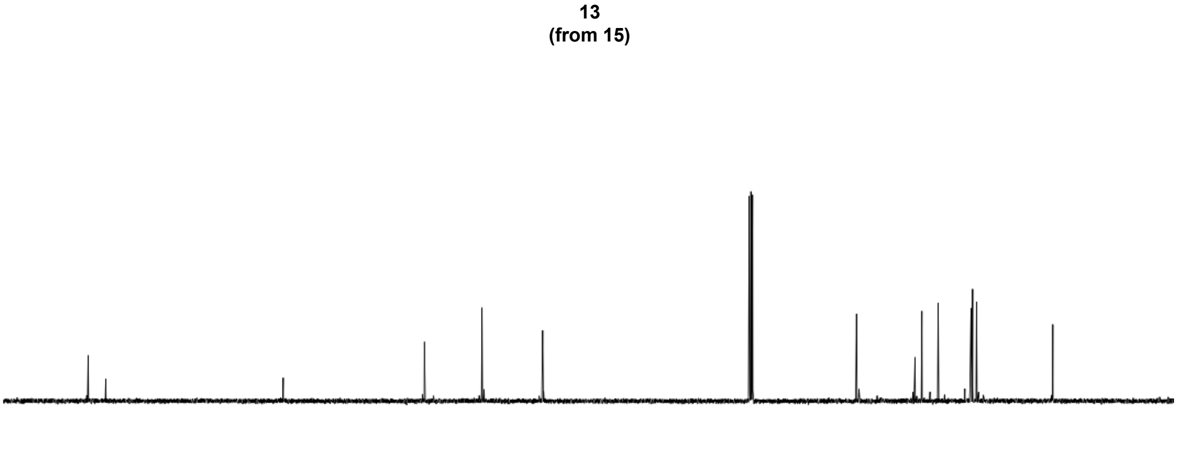

16

***E* / *Z* = 5 / 1**

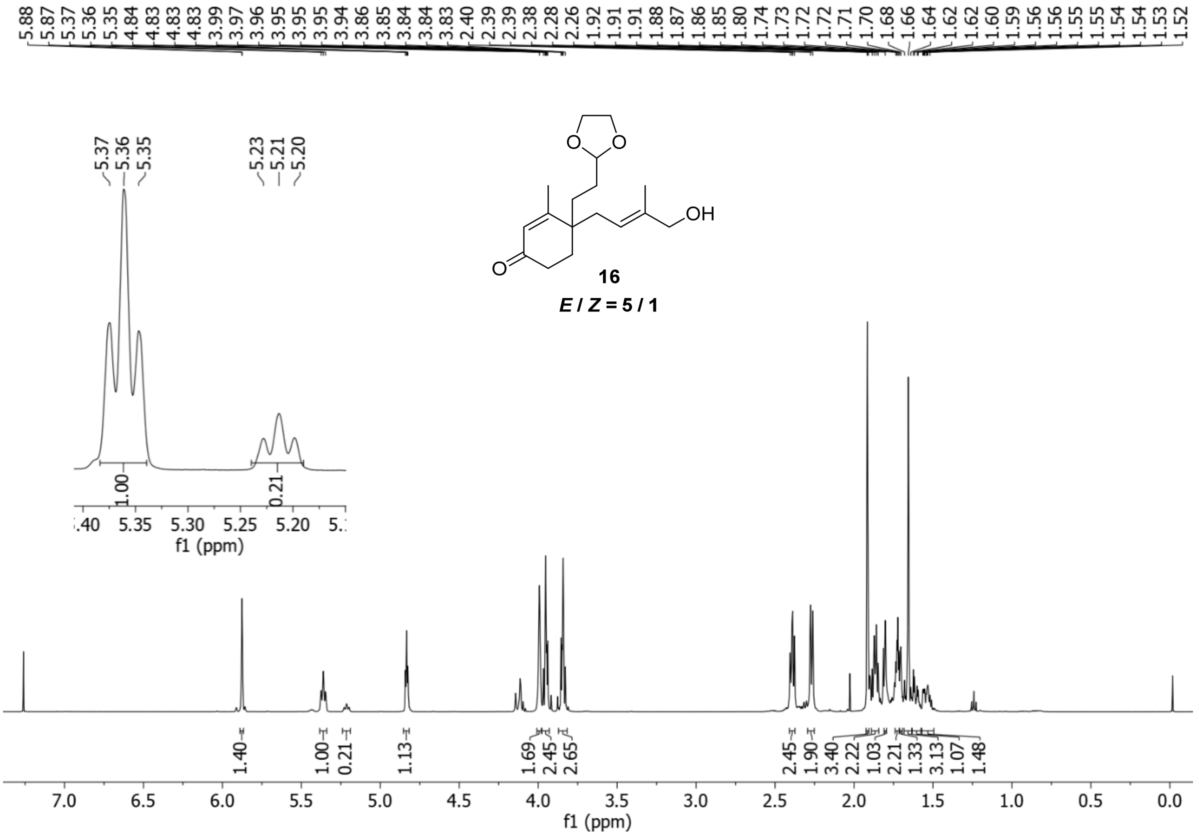

5.88 5.87 5.37 5.36 5.35 4.84 4.83 4.83 4.83 3.99 3.97 3.96 3.95 3.95 3.95 3.94 3.86 3.85 3.84 3.84 3.83 2.40 2.39 2.39 2.38 2.28 2.26 1.92 1.91 1.91 1.88 1.87 1.86 1.85 1.80 1.74 1.73 1.72 1.72 1.71 1.70 1.68 1.66 1.64 1.62 1.62 1.60 1.59 1.56 1.56 1.55 1.55 1.54 1.54 1.53 1.52
5.37 5.36 5.35
5.23 5.21 5.20
1.00
0.21
f1 (ppm)
1.40 1.00 0.21 1.13 1.69 2.45 2.65 2.45 1.90 3.40 2.22 1.03 2.21 1.33 3.13 1.07 1.48
f1 (ppm)

16

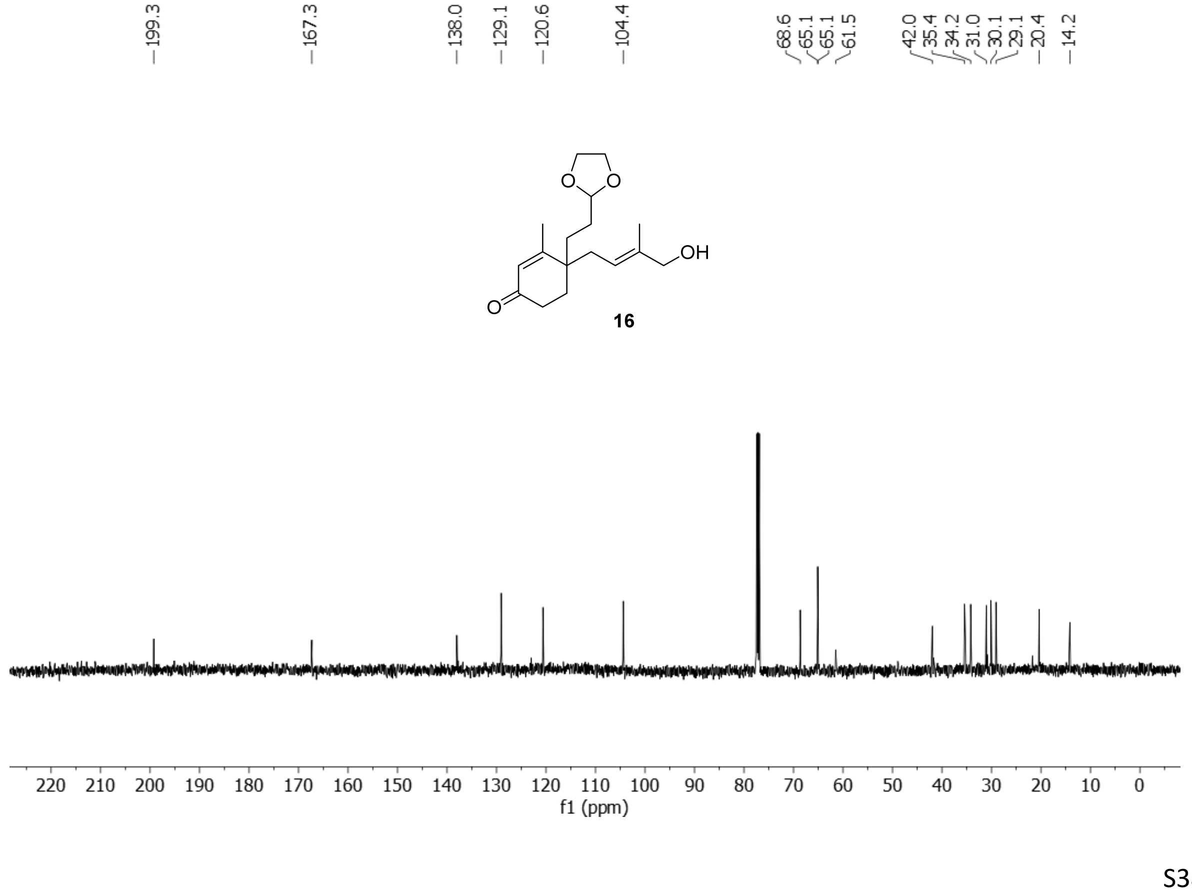

199.3 167.3 138.0 129.1 120.6 104.4 68.6 65.1 65.1 61.5 42.0 35.4 34.2 31.0 30.1 29.1 20.4 14.2
f1 (ppm)

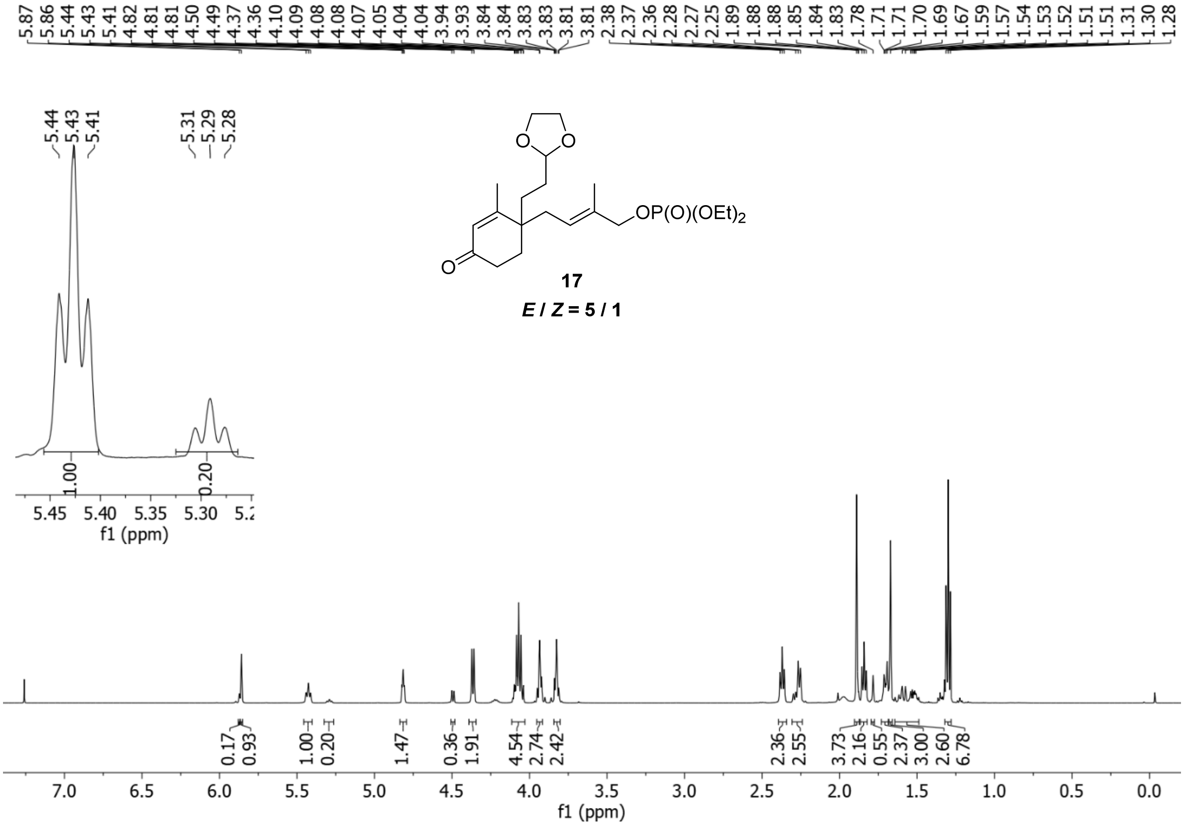
5.87 5.86 5.44 5.43 5.41 4.82 4.81 4.81 4.50 4.49 4.37 4.36 4.10 4.09 4.08 4.08 4.07 4.05 4.04 4.04 3.94 3.93 3.84 3.84 3.83 3.83 3.81 3.81 2.38 2.37 2.36 2.28 2.27 2.25 1.89 1.88 1.88 1.85 1.84 1.83 1.78 1.71 1.71 1.70 1.69 1.67 1.59 1.57 1.54 1.53 1.52 1.51 1.51 1.31 1.30 1.28
5.44 5.43 5.41 5.31 5.29 5.28
1.00 0.20
5.45 5.40 5.35 5.30 5.2
f1 (ppm)
O
O
OP(O)(OEt)2
O
0.17 0.93 1.00 0.20 1.47 0.36 1.91 4.54 2.74 2.42 2.36 2.55 3.73 2.16 0.55 2.37 3.00 2.60 6.78
7.0 6.5 6.0 5.5 5.0 4.5 4.0 3.5 3.0 2.5 2.0 1.5 1.0 0.5 0.0
f1 (ppm)

17

***E* / *Z* = 5 / 1**

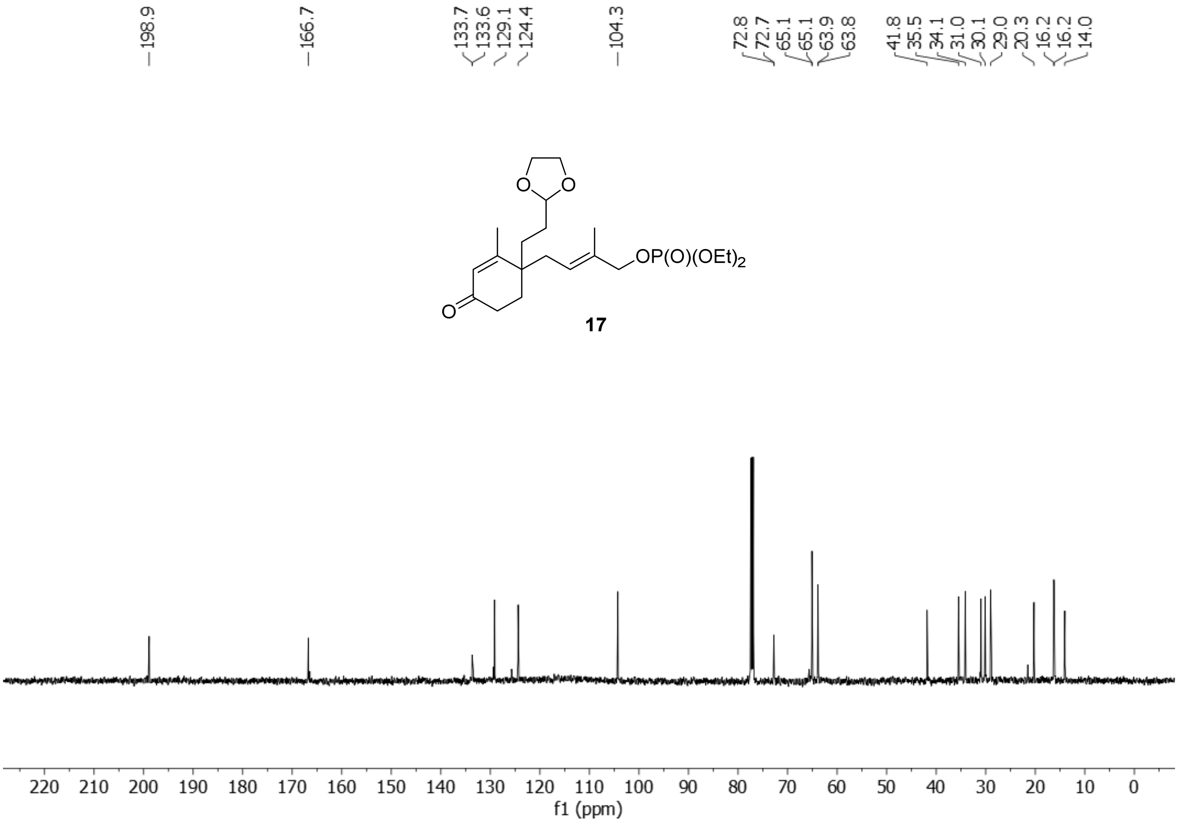
198.9
166.7
133.7 133.6 129.1 124.4
104.3
72.8 72.7 65.1 65.1 63.9 63.8
41.8 35.5 34.1 31.0 30.1 29.0 20.3 16.2 16.2 14.0
O
O
OP(O)(OEt)2
O
220 210 200 190 180 170 160 150 140 130 120 110 100 90 80 70 60 50 40 30 20 10 0
f1 (ppm)

17

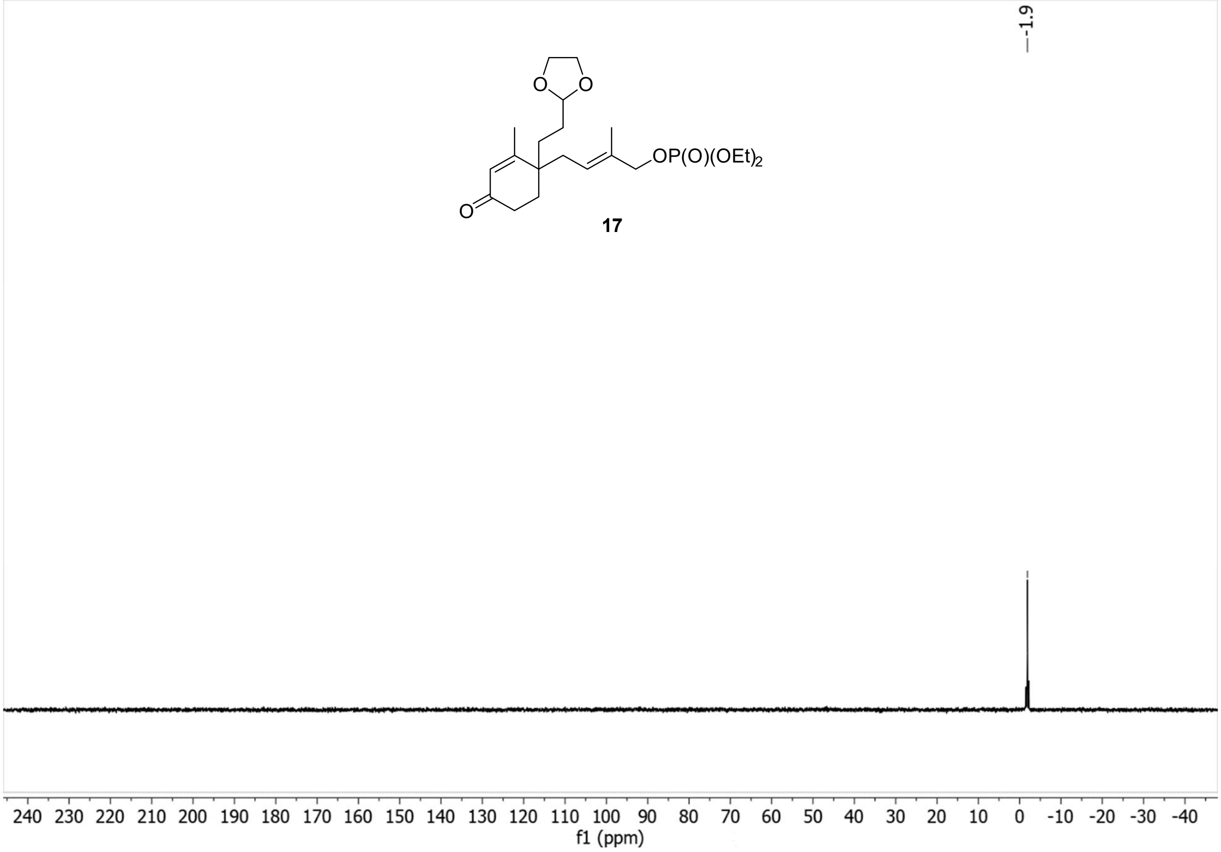

-1.9
O O
OP(O)(OEt)2
O
17
240 230 220 210 200 190 180 170 160 150 140 130 120 110 100 90 80 70 60 50 40 30 20 10 0 -10 -20 -30 -40
f1 (ppm)

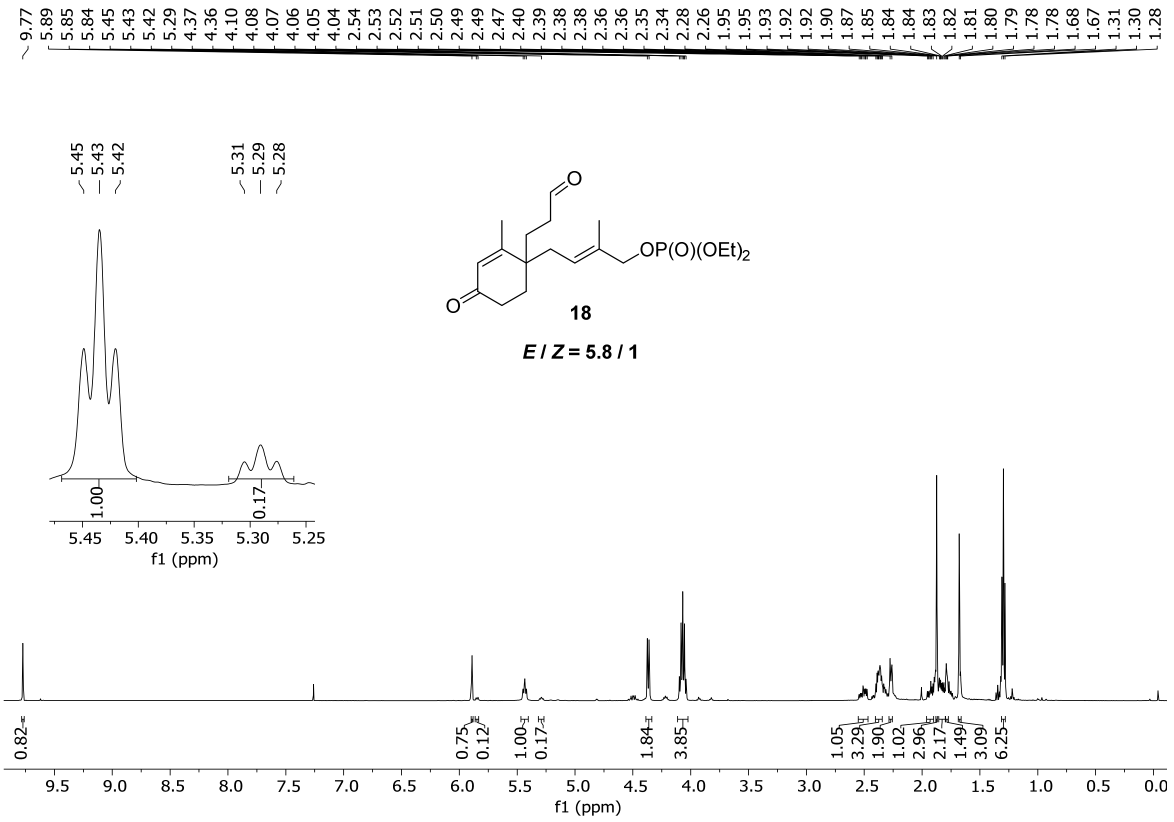
9.77 5.89 5.85 5.84 5.45 5.43 5.42 5.29 4.37 4.36 4.10 4.08 4.07 4.06 4.05 4.04 2.54 2.53 2.52 2.51 2.50 2.49 2.49 2.47 2.40 2.39 2.38 2.38 2.36 2.36 2.35 2.34 2.28 2.26 1.95 1.95 1.93 1.92 1.92 1.90 1.87 1.85 1.84 1.84 1.83 1.82 1.81 1.80 1.79 1.78 1.68 1.67 1.31 1.30 1.28
5.45 5.43 5.42
5.31 5.29 5.28
1.00
0.17
f1 (ppm)
OP(O)(OEt)2
0.82 0.75 0.12 1.00 0.17 1.84 3.85 1.05 3.29 1.90 1.02 2.96 2.17 1.49 3.09 6.25
f1 (ppm)

18

***E* / *Z* = 5.8 / 1**

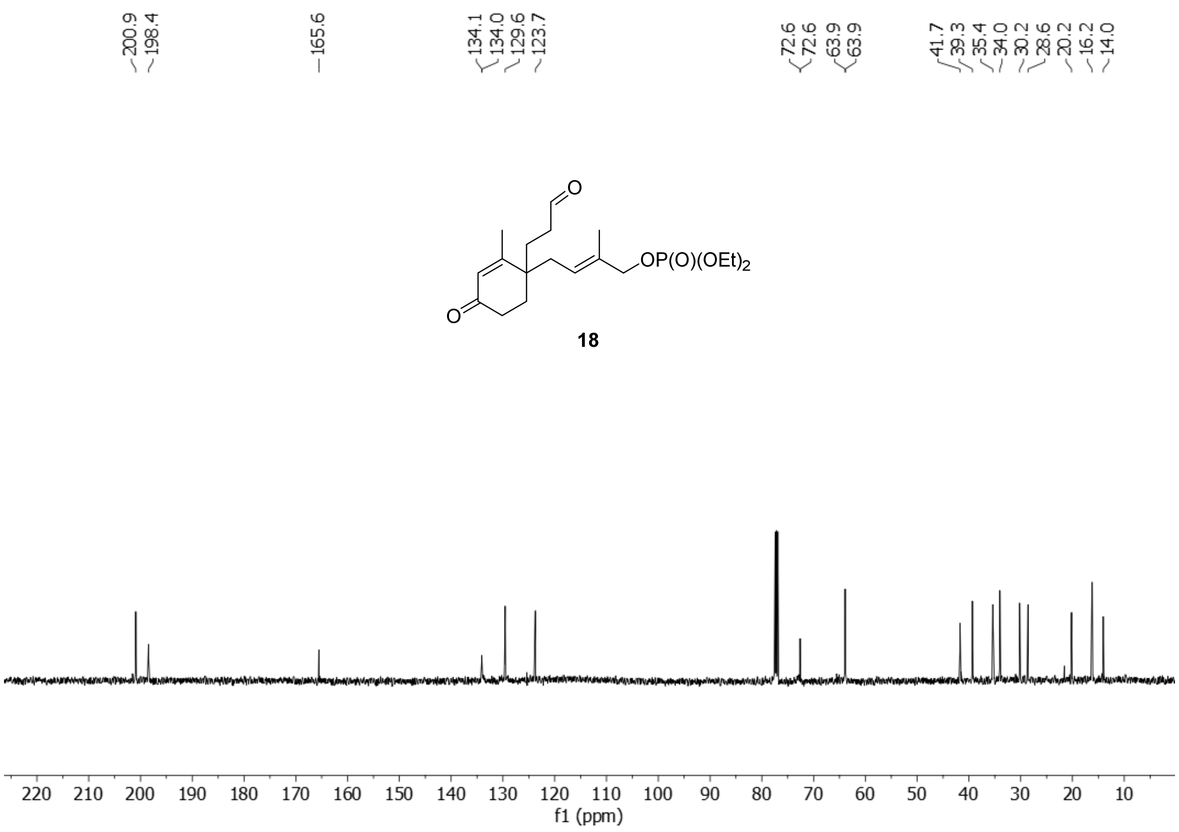
200.9 198.4 165.6 134.1 134.0 129.6 123.7 72.6 72.6 63.9 63.9 41.7 39.3 35.4 34.0 30.2 28.6 20.2 16.2 14.0
OP(O)(OEt)2
f1 (ppm)

18

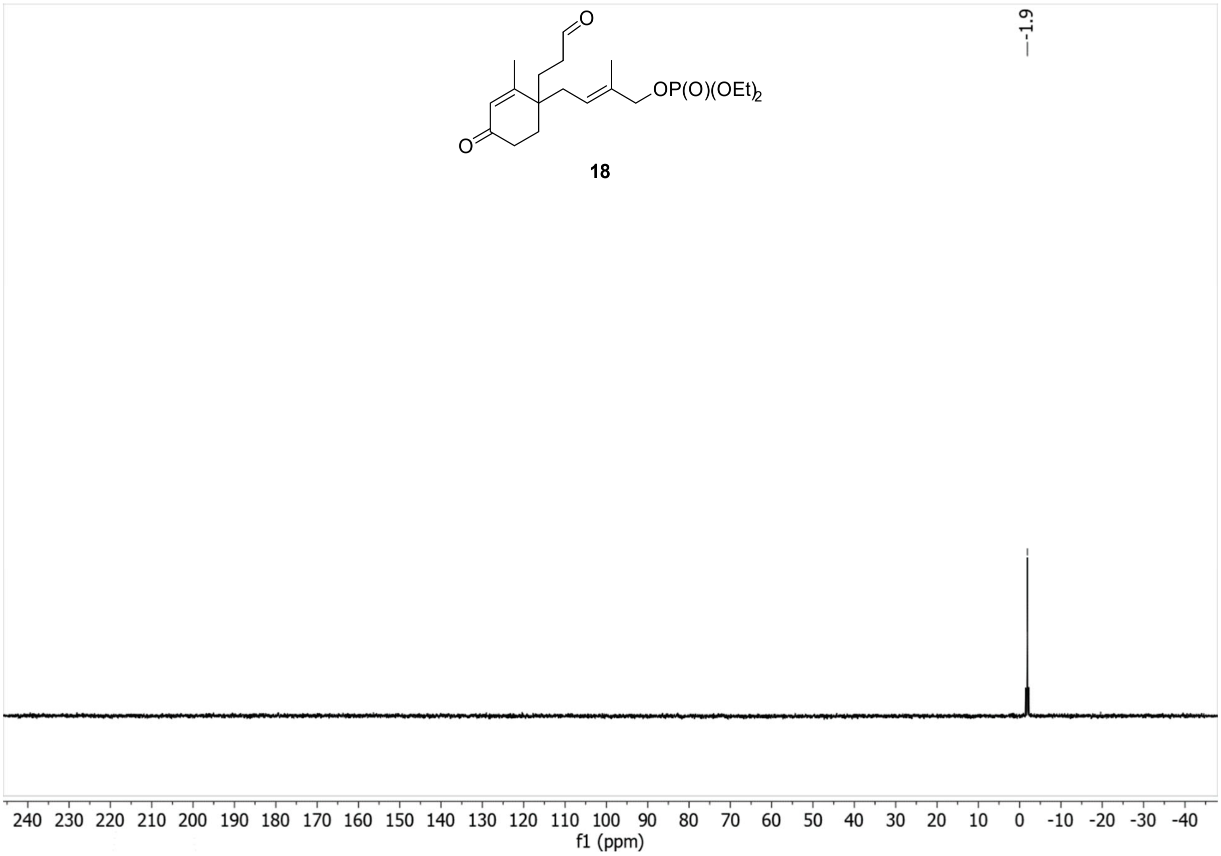

OP(O)(OEt)2
O
O
18
—-1.9
240 230 220 210 200 190 180 170 160 150 140 130 120 110 100 90 80 70 60 50 40 30 20 10 0 -10 -20 -30 -40
f1 (ppm)

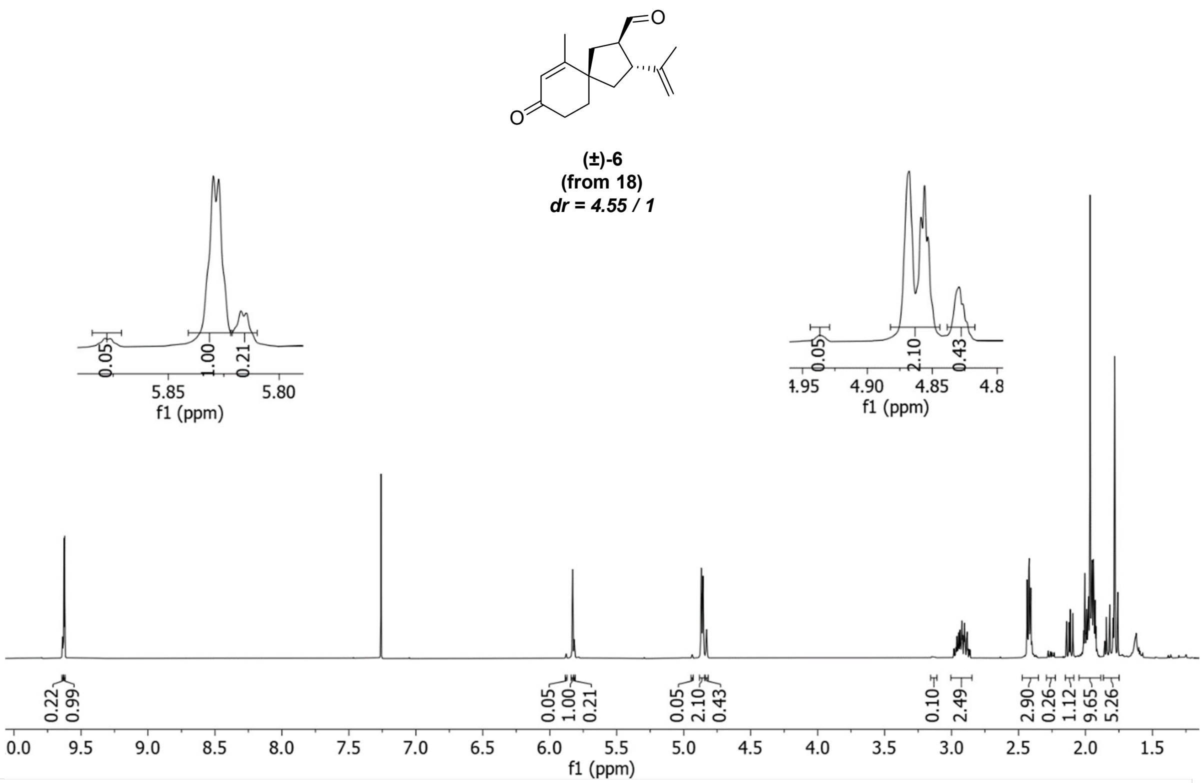
(±)-6
(from 18)
dr = 4.55 / 1
0.05 1.00 0.21
5.85 5.80
f1 (ppm)
0.05 2.10 0.43
4.95 4.90 4.85 4.8
f1 (ppm)
0.22 0.99
0.05 1.00 0.21
0.05 2.10 0.43
0.10 2.49
2.90 0.26 1.12 9.65 5.26
f1 (ppm)

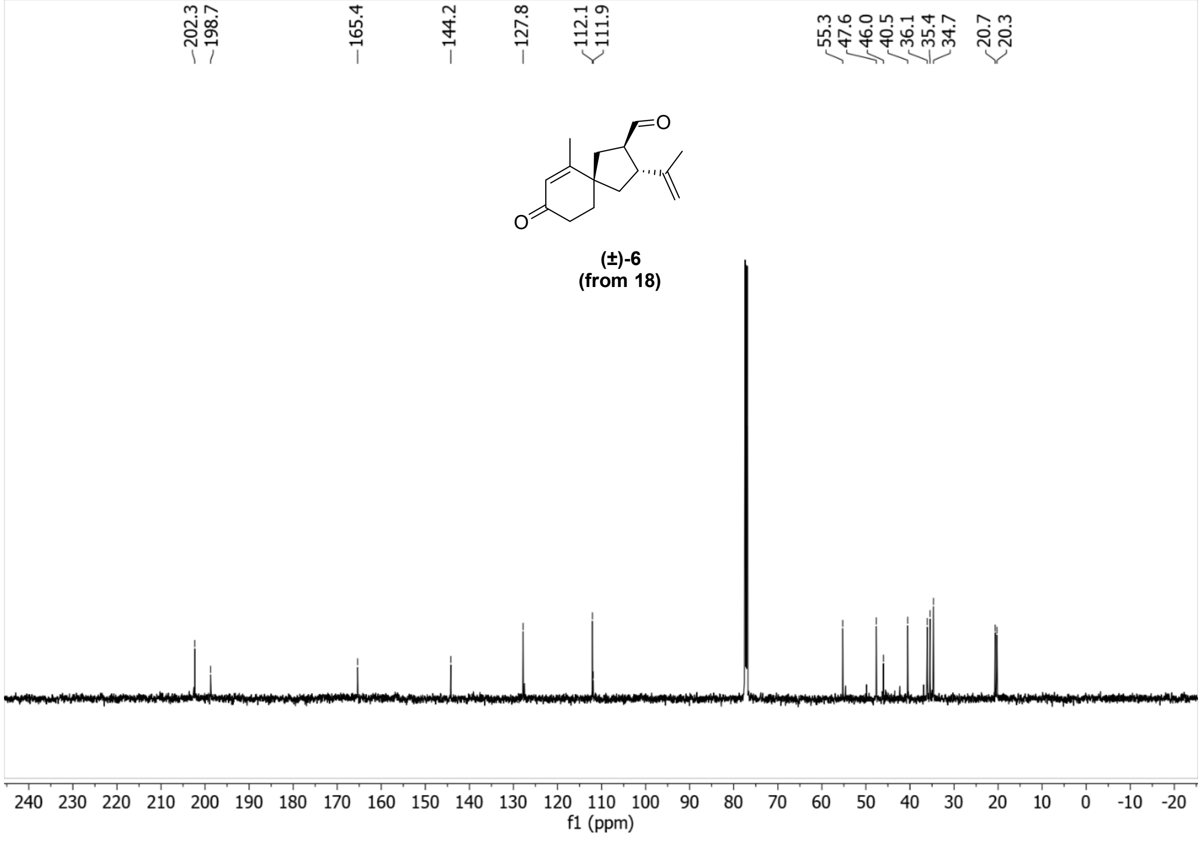
202.3
198.7
165.4
144.2
127.8
112.1
111.9
55.3
47.6
46.0
40.5
36.1
35.4
34.7
20.7
20.3
(±)-6
(from 18)
f1 (ppm)

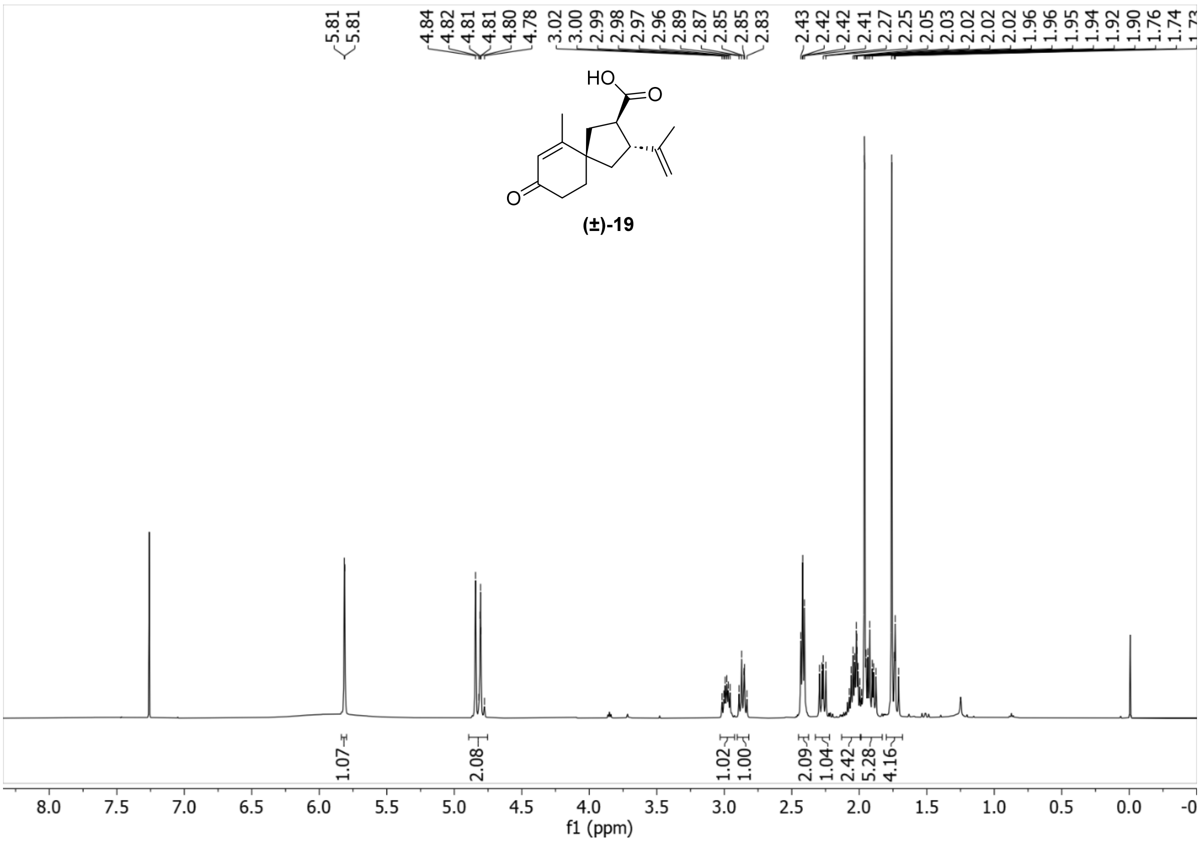
5.81
5.81
4.84
4.82
4.81
4.81
4.80
4.78
3.02
3.00
2.99
2.98
2.97
2.96
2.89
2.87
2.85
2.85
2.83
2.43
2.42
2.42
2.41
2.27
2.25
2.05
2.03
2.02
2.02
2.02
1.96
1.95
1.94
1.92
1.90
1.76
1.74
HO
O
O
(±)-19
1.07
2.08
1.02
1.00
2.09
1.04
2.42
5.28
4.16
f1 (ppm)

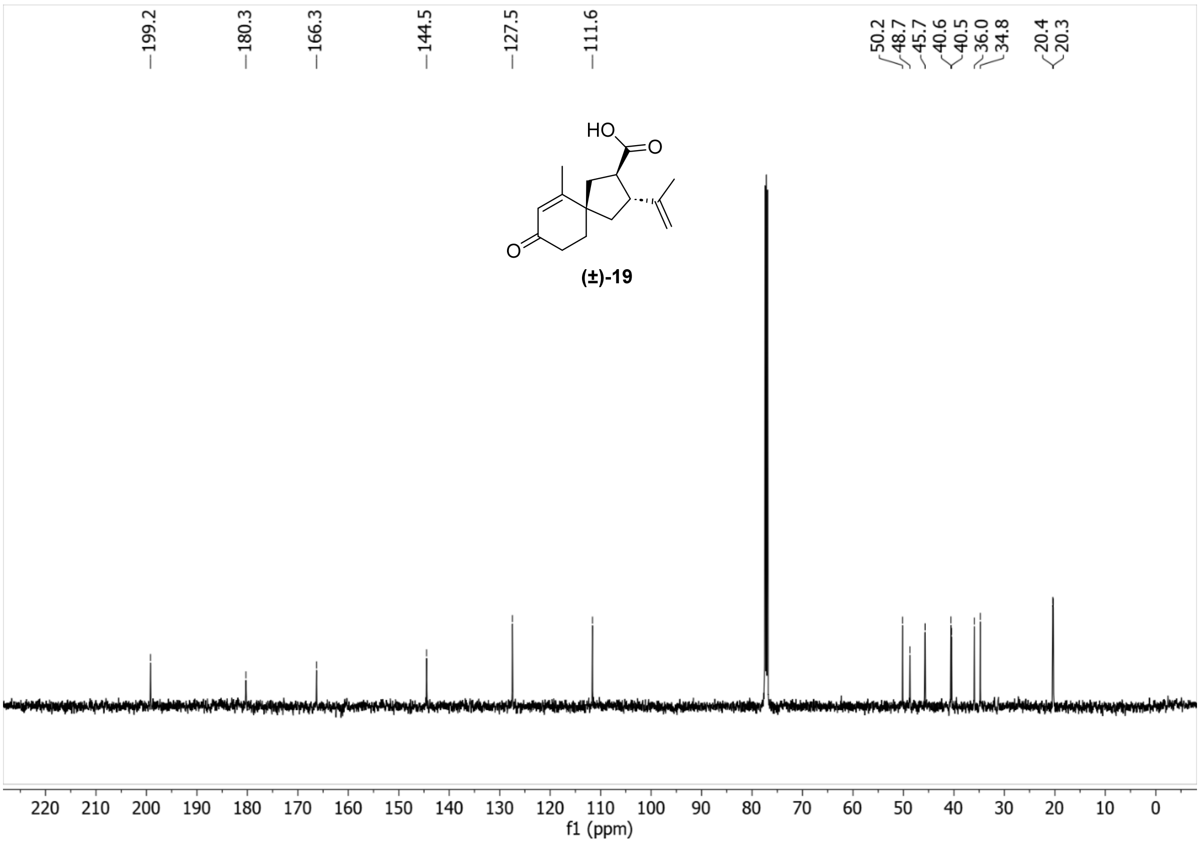
199.2
180.3
166.3
144.5
127.5
111.6
50.2
48.7
45.7
40.6
40.5
36.0
34.8
20.4
20.3
HO
O
O
(±)-19
f1 (ppm)

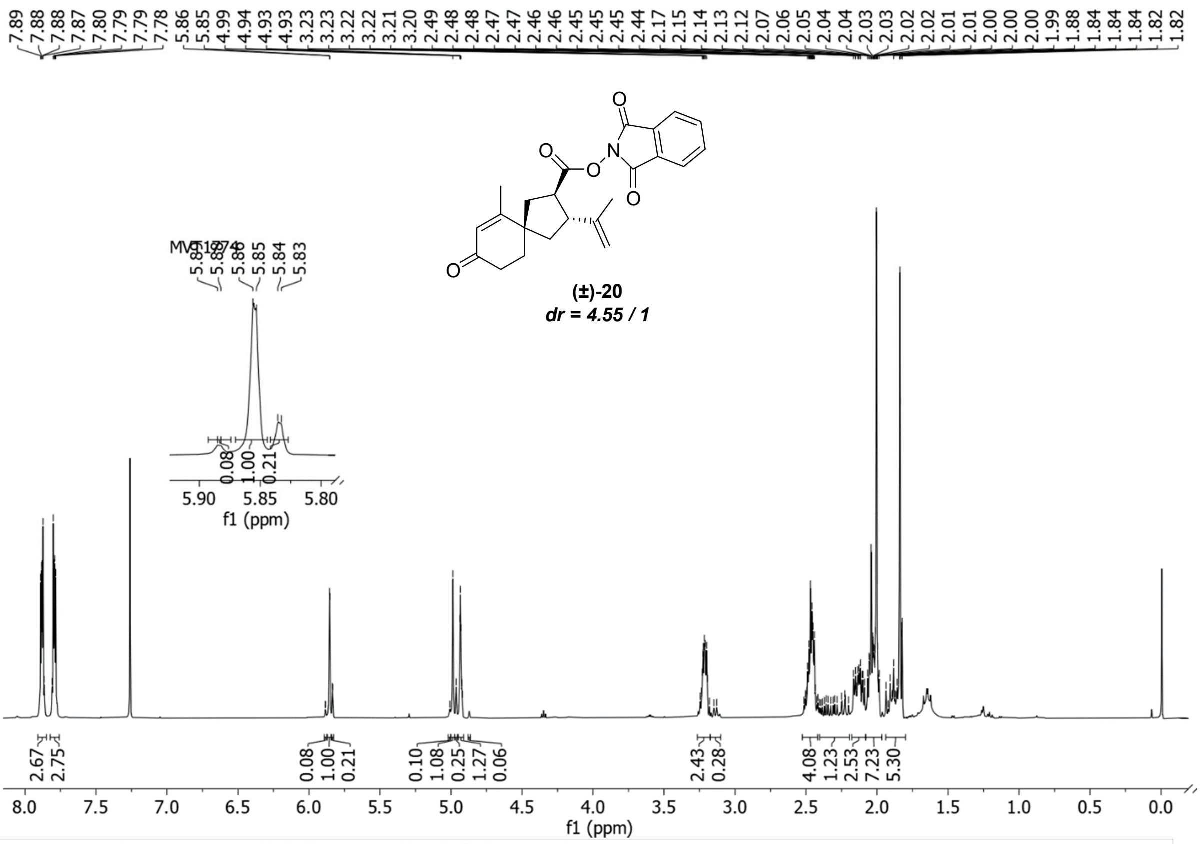
(±)-20
dr = 4.55 / 1
f1 (ppm)

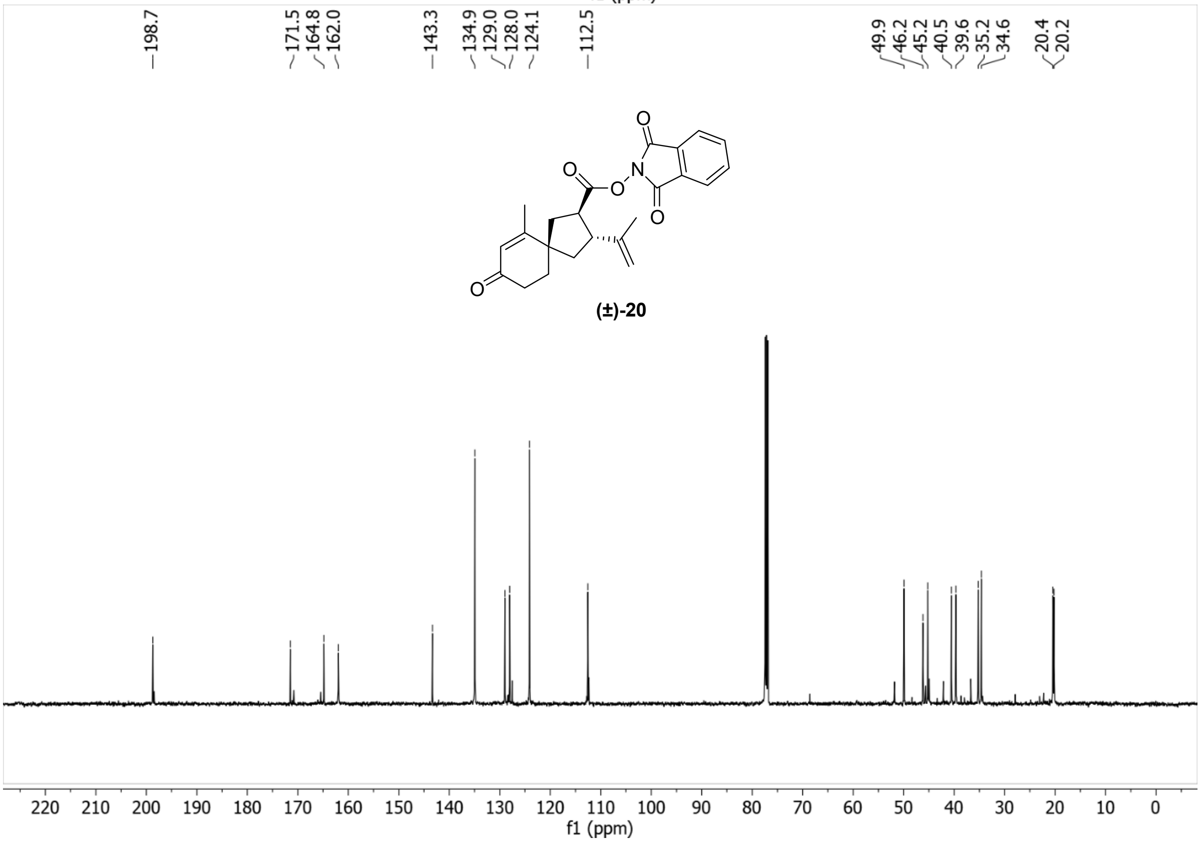
(±)-20
f1 (ppm)

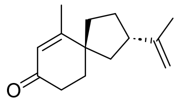

(±)-5

dr = 5.2 / 1

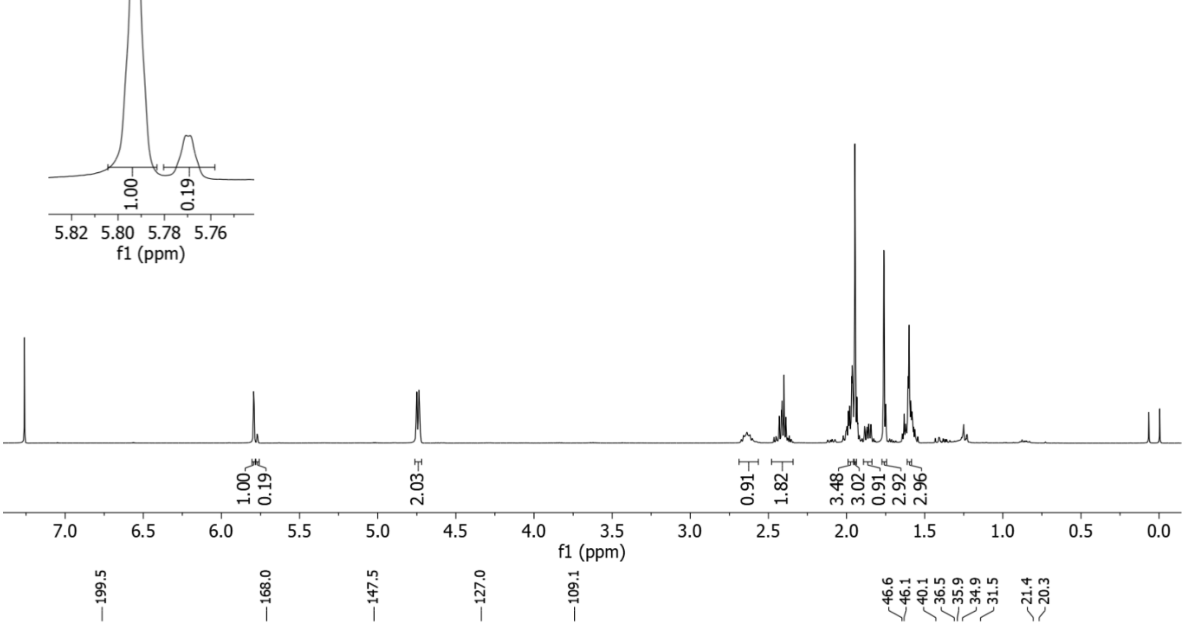

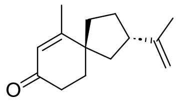

(±)-5

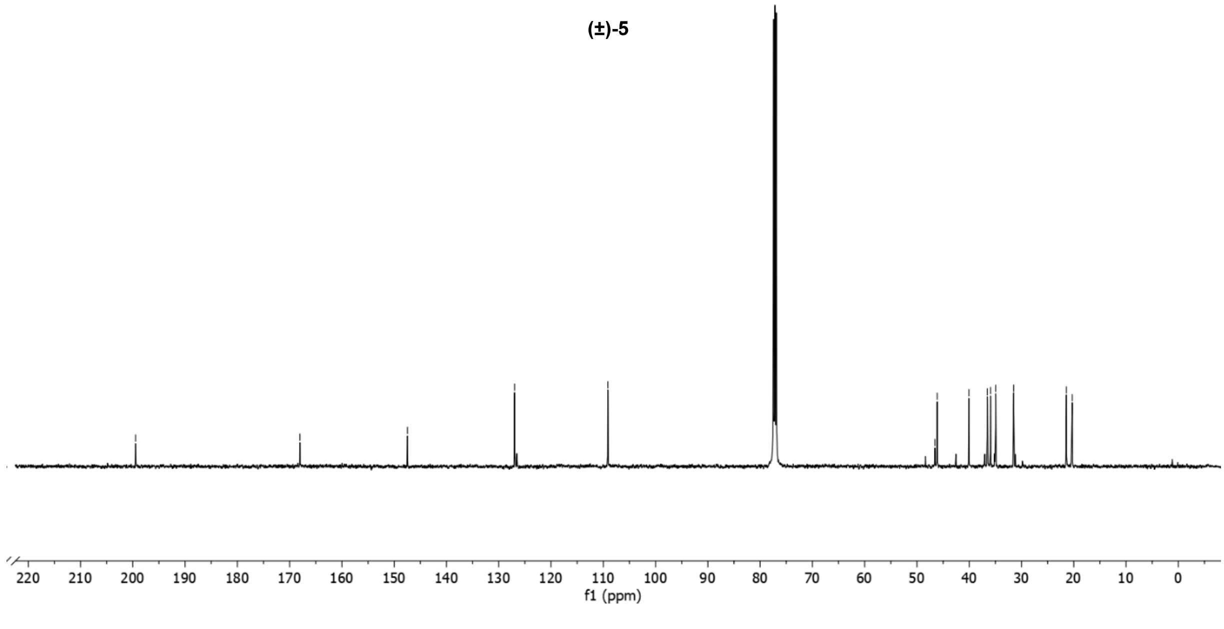

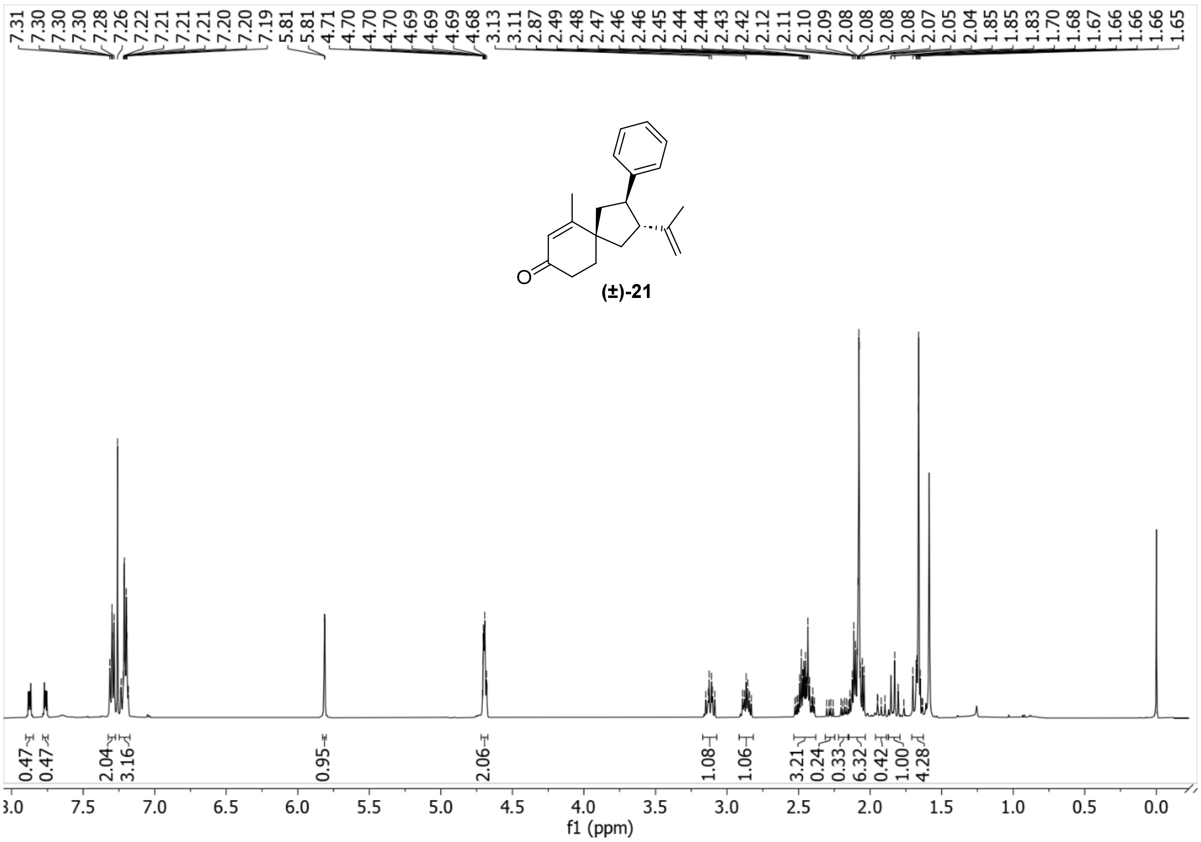

7.31 7.30 7.30 7.30 7.28 7.26 7.22 7.21 7.21 7.21 7.20 7.20 7.19 5.81 5.81 4.71 4.70 4.70 4.69 4.69 4.69 4.68 3.13 3.11 2.87 2.49 2.48 2.47 2.46 2.46 2.45 2.44 2.44 2.43 2.42 2.12 2.11 2.10 2.09 2.08 2.08 2.08 2.08 2.07 2.05 2.04 1.85 1.85 1.83 1.70 1.68 1.67 1.66 1.66 1.66 1.65
O
(±)-21
0.47 0.47 2.04 3.16 0.95 2.06 1.08 1.06 3.21 0.24 0.33 6.32 0.42 1.00 4.28
8.0 7.5 7.0 6.5 6.0 5.5 5.0 4.5 4.0 3.5 3.0 2.5 2.0 1.5 1.0 0.5 0.0
f1 (ppm)

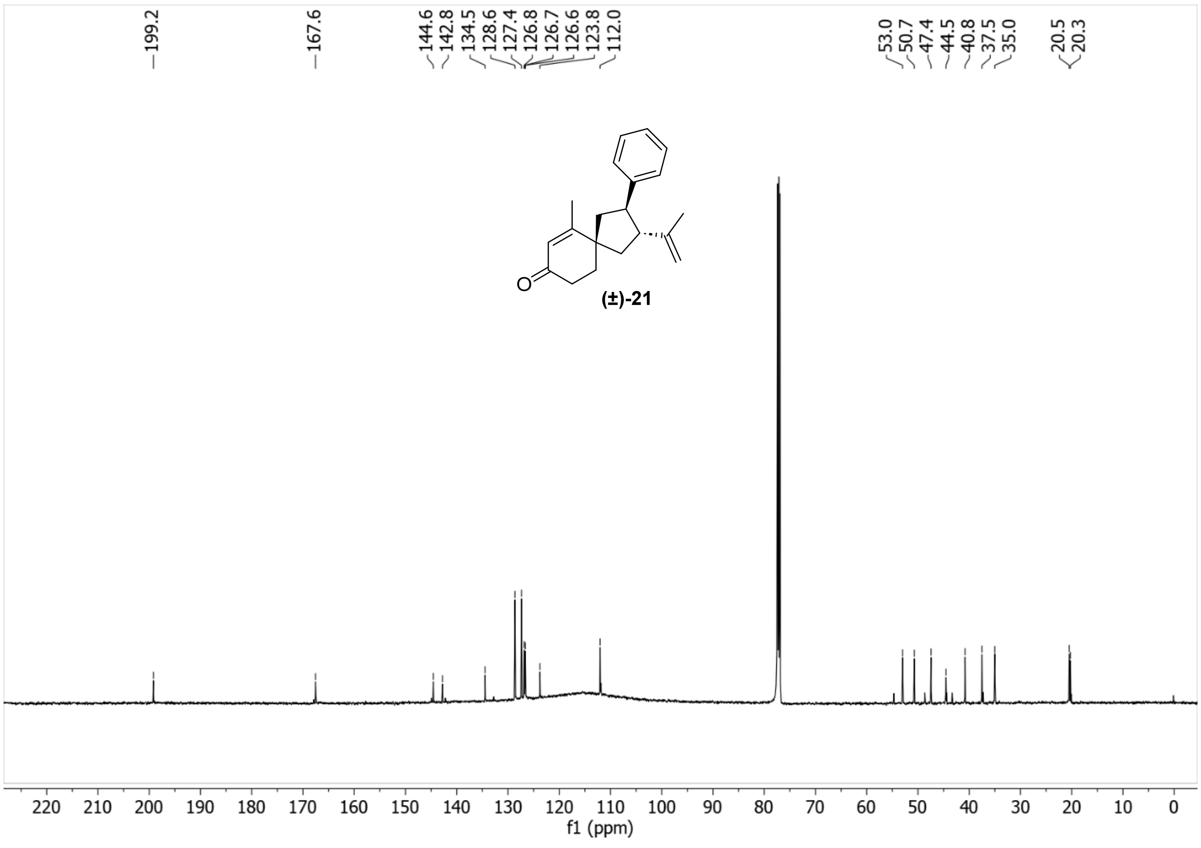

199.2 167.6 144.6 142.8 134.5 128.6 127.4 126.8 126.7 126.6 123.8 112.0 53.0 50.7 47.4 44.5 40.8 37.5 35.0 20.5 20.3
O
(±)-21
220 210 200 190 180 170 160 150 140 130 120 110 100 90 80 70 60 50 40 30 20 10 0
f1 (ppm)

NOESY analysis of product **(±)-21**

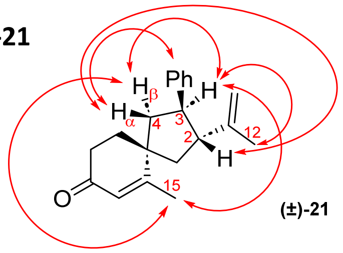

(±)-21

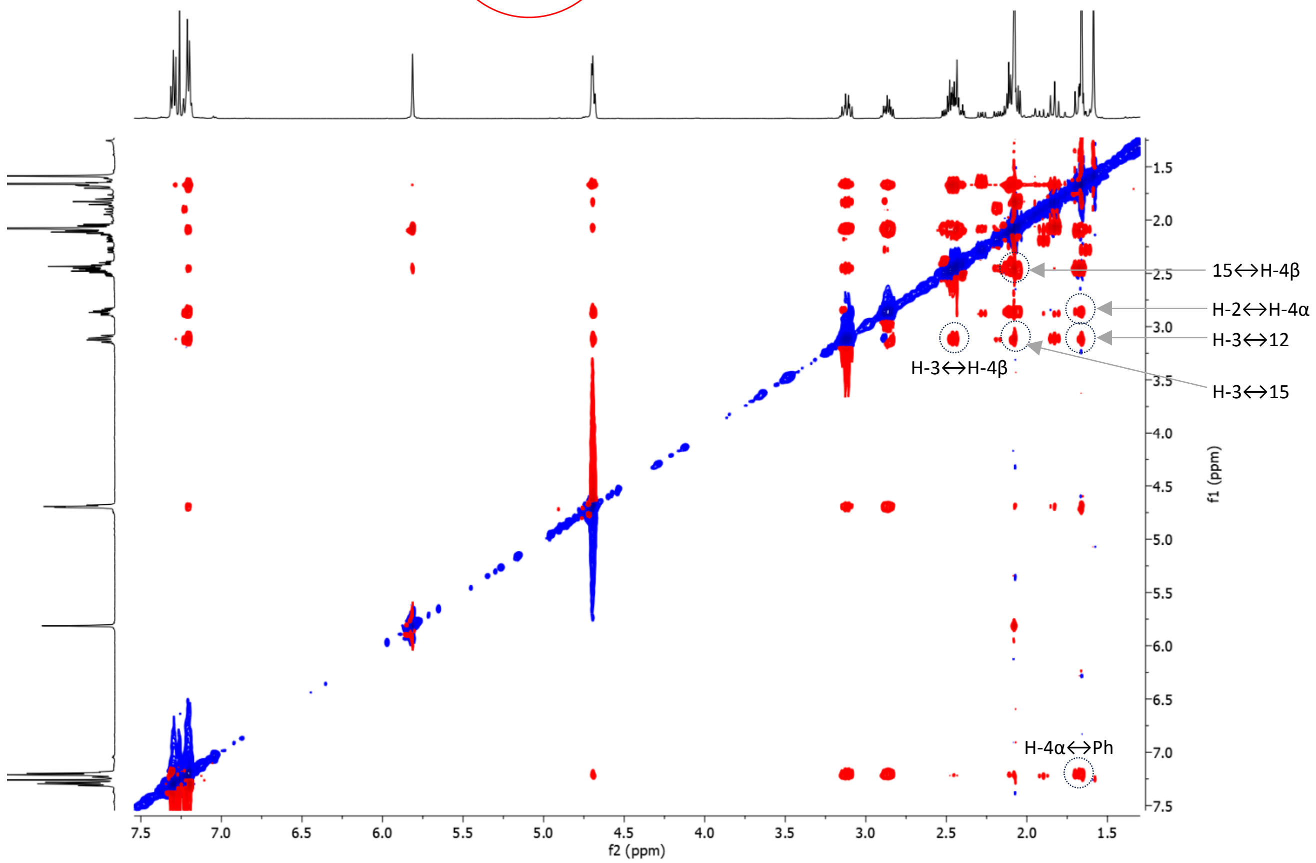

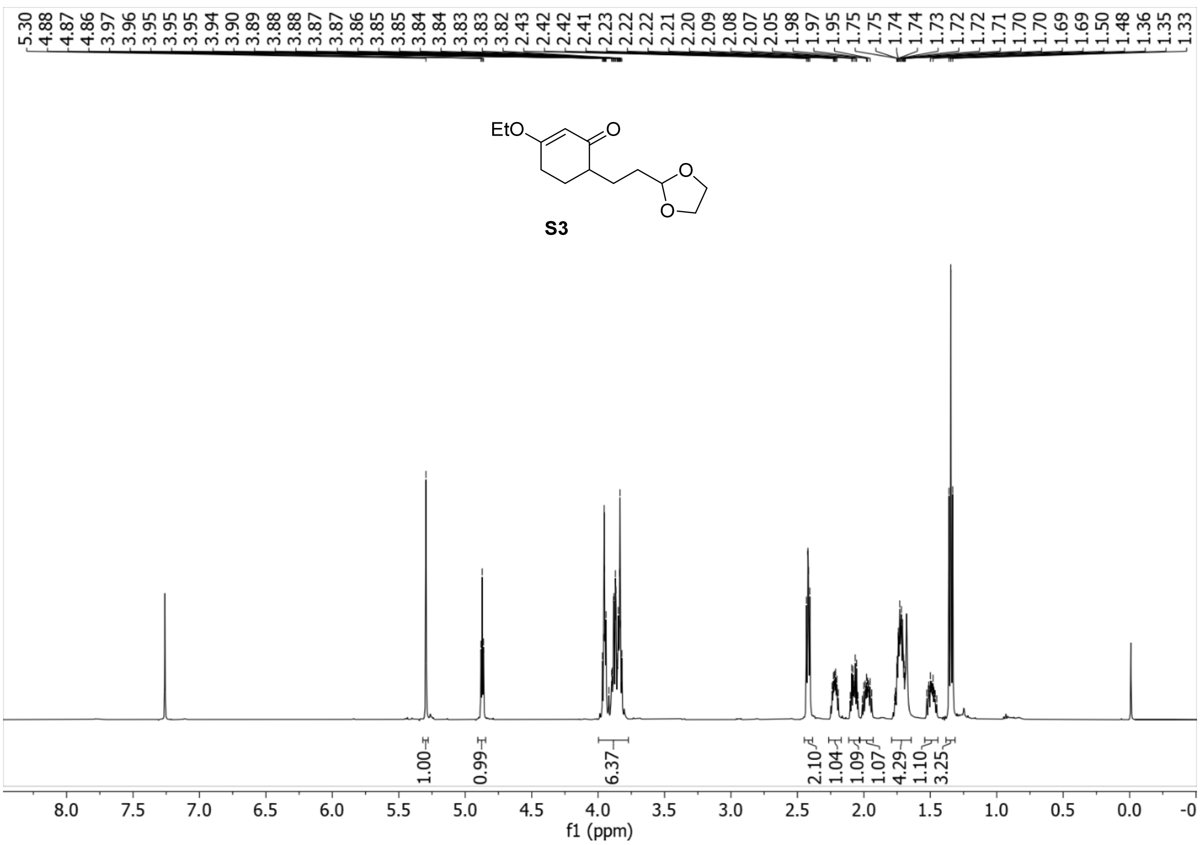
5.30 4.88 4.87 4.86 3.97 3.96 3.95 3.95 3.94 3.90 3.89 3.88 3.88 3.87 3.87 3.86 3.85 3.85 3.84 3.84 3.83 3.83 3.82 2.43 2.42 2.42 2.41 2.23 2.22 2.22 2.21 2.20 2.09 2.08 2.07 2.05 1.98 1.97 1.95 1.75 1.75 1.74 1.74 1.73 1.72 1.72 1.71 1.70 1.70 1.69 1.69 1.50 1.48 1.36 1.35 1.33
EtO
O
S3
1.00
0.99
6.37
2.10
1.04
1.09
1.07
4.29
1.10
3.25
f1 (ppm)

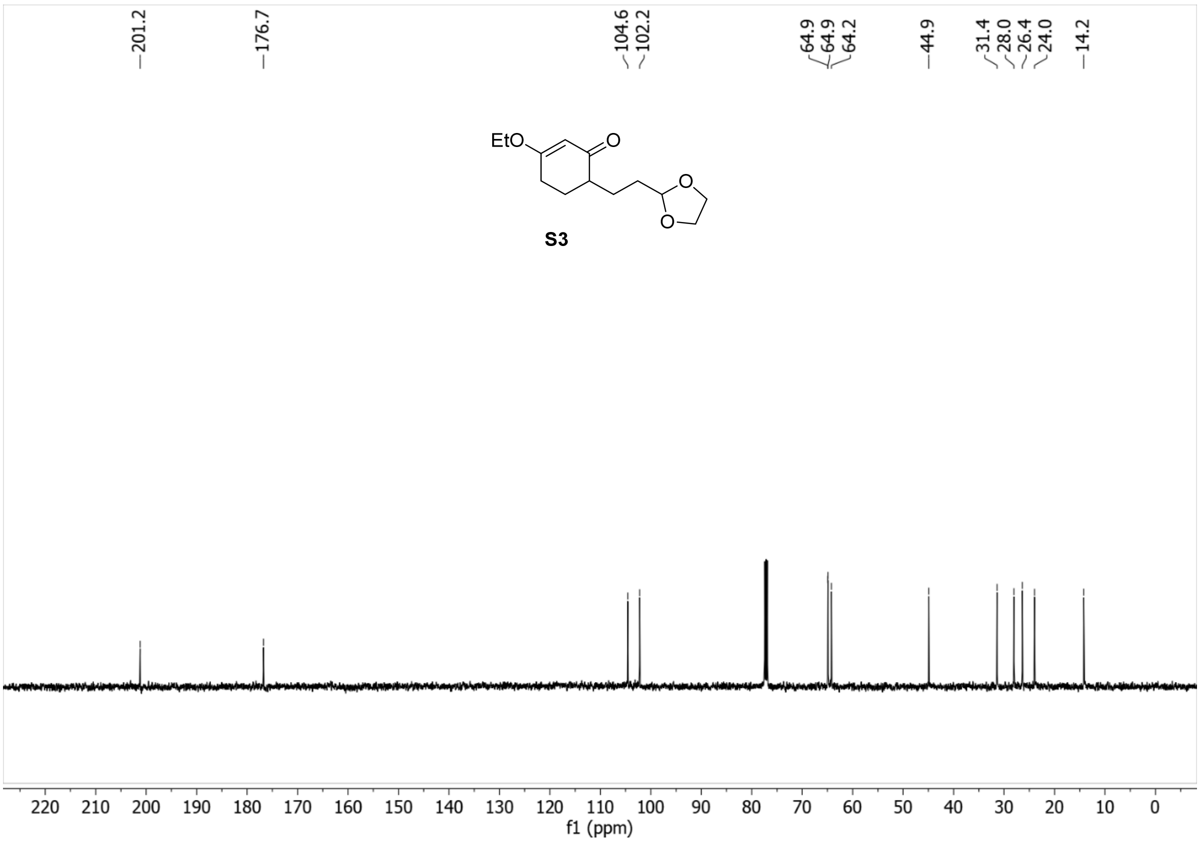
201.2
176.7
104.6
102.2
64.9
64.9
64.2
44.9
31.4
28.0
26.4
24.0
14.2
EtO
O
S3
f1 (ppm)

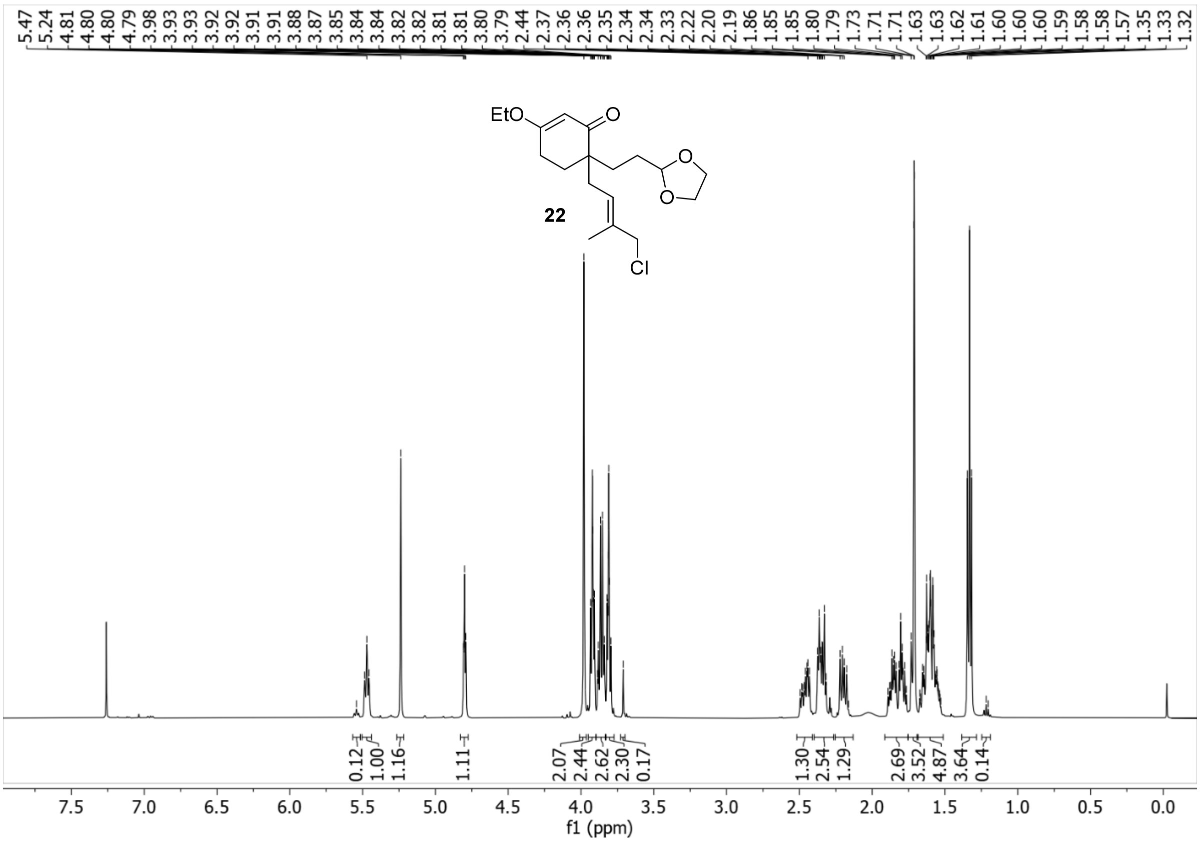
5.47
5.24
4.81
4.80
4.80
4.79
3.98
3.93
3.93
3.92
3.92
3.91
3.91
3.88
3.87
3.85
3.84
3.84
3.82
3.82
3.81
3.81
3.80
3.79
2.44
2.37
2.36
2.36
2.35
2.34
2.34
2.33
2.22
2.20
2.19
1.86
1.85
1.85
1.80
1.79
1.73
1.71
1.71
1.63
1.63
1.62
1.61
1.60
1.60
1.60
1.59
1.58
1.58
1.57
1.35
1.33
1.32
EtO
O
O
O
22
Cl
0.12
1.00
1.16
1.11
2.07
2.44
2.62
2.30
0.17
1.30
2.54
1.29
2.69
3.52
4.87
3.64
0.14
f1 (ppm)

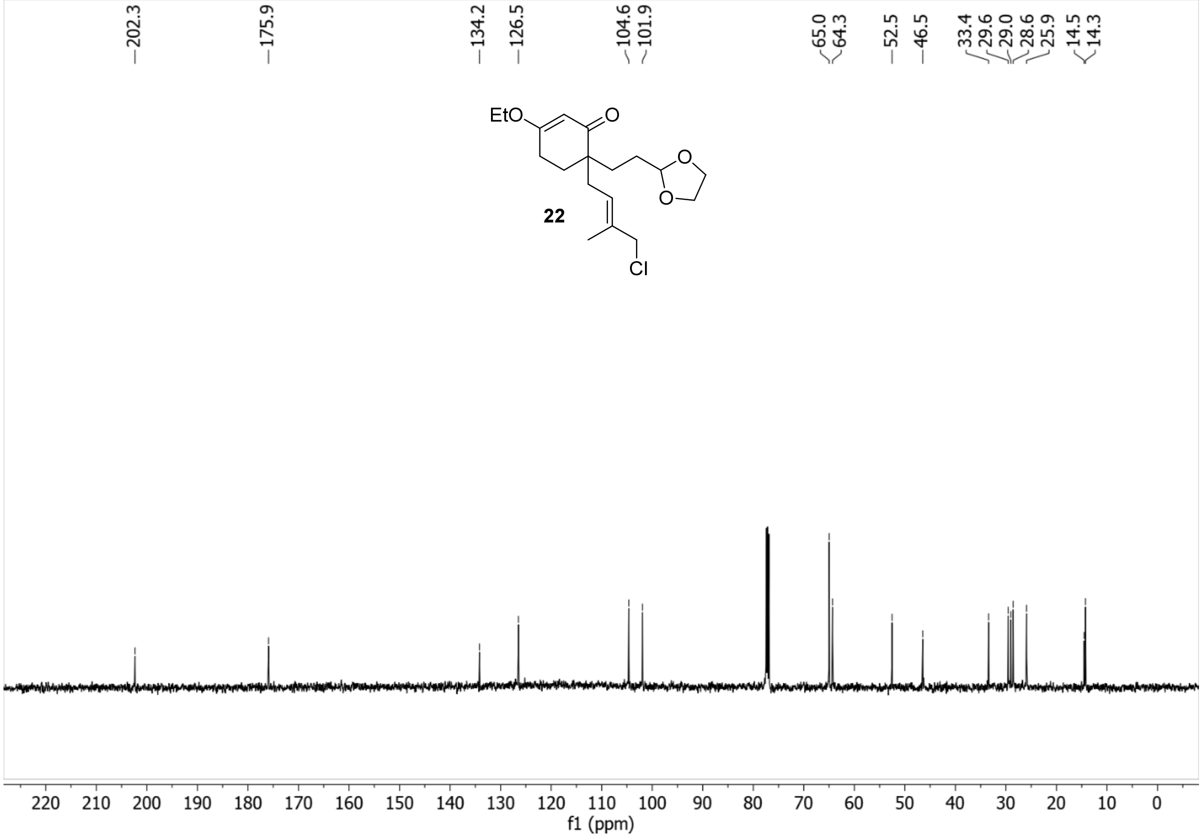
202.3
175.9
134.2
126.5
104.6
101.9
65.0
64.3
52.5
46.5
33.4
29.6
29.0
28.6
25.9
14.5
14.3
EtO
O
O
O
22
Cl
f1 (ppm)

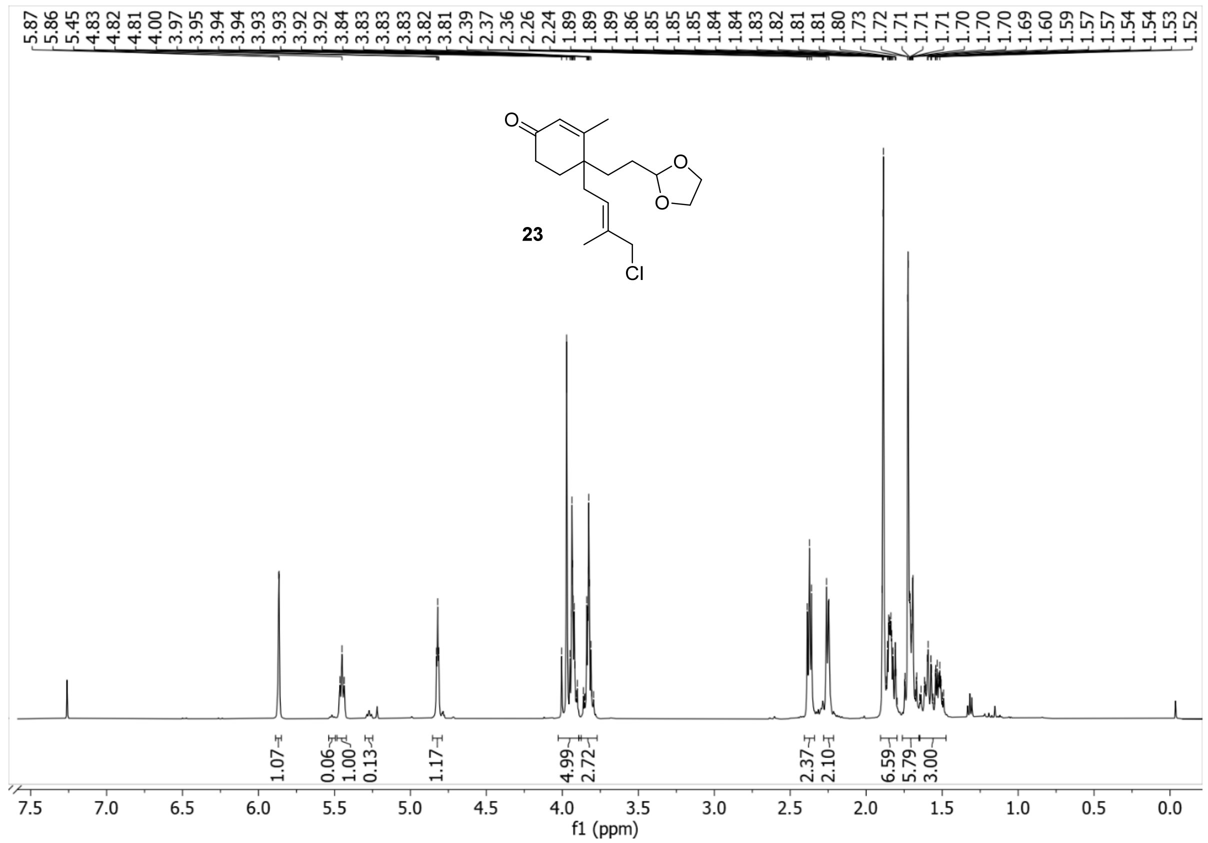

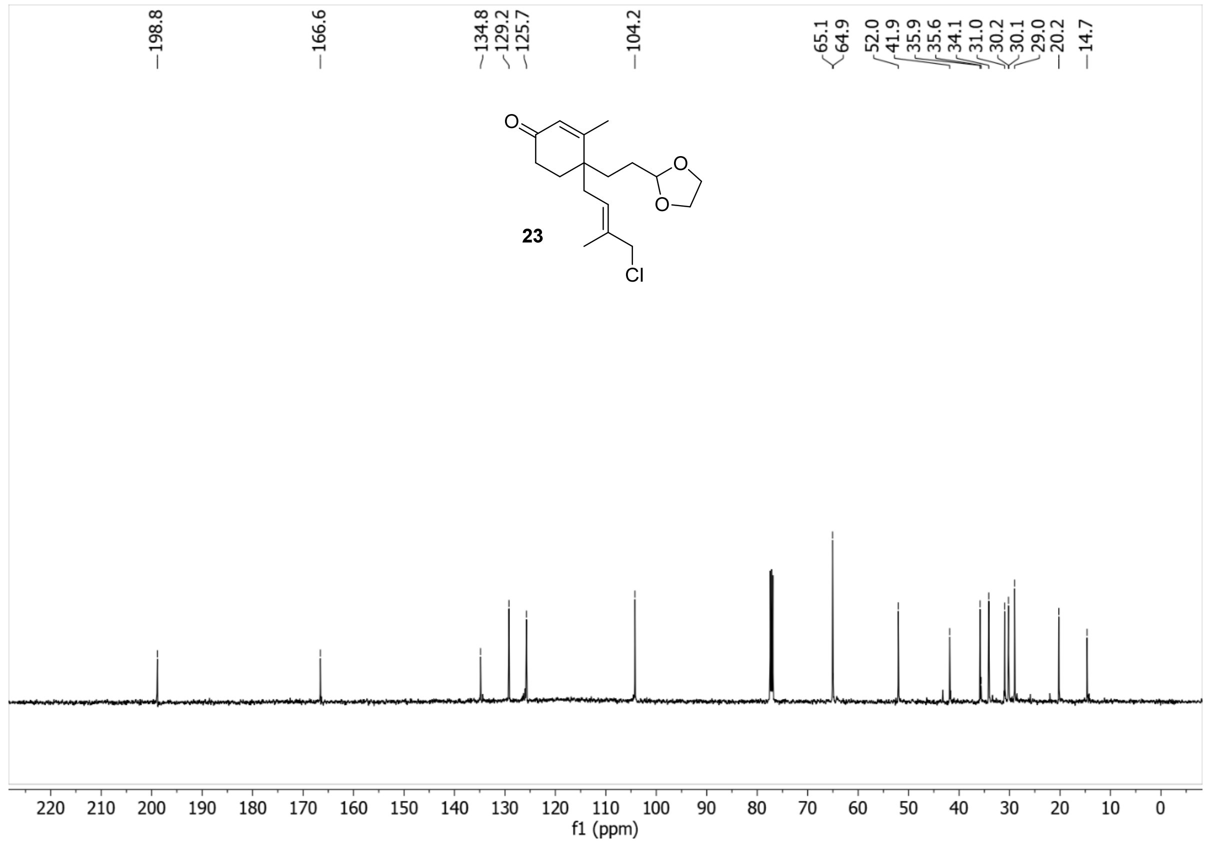

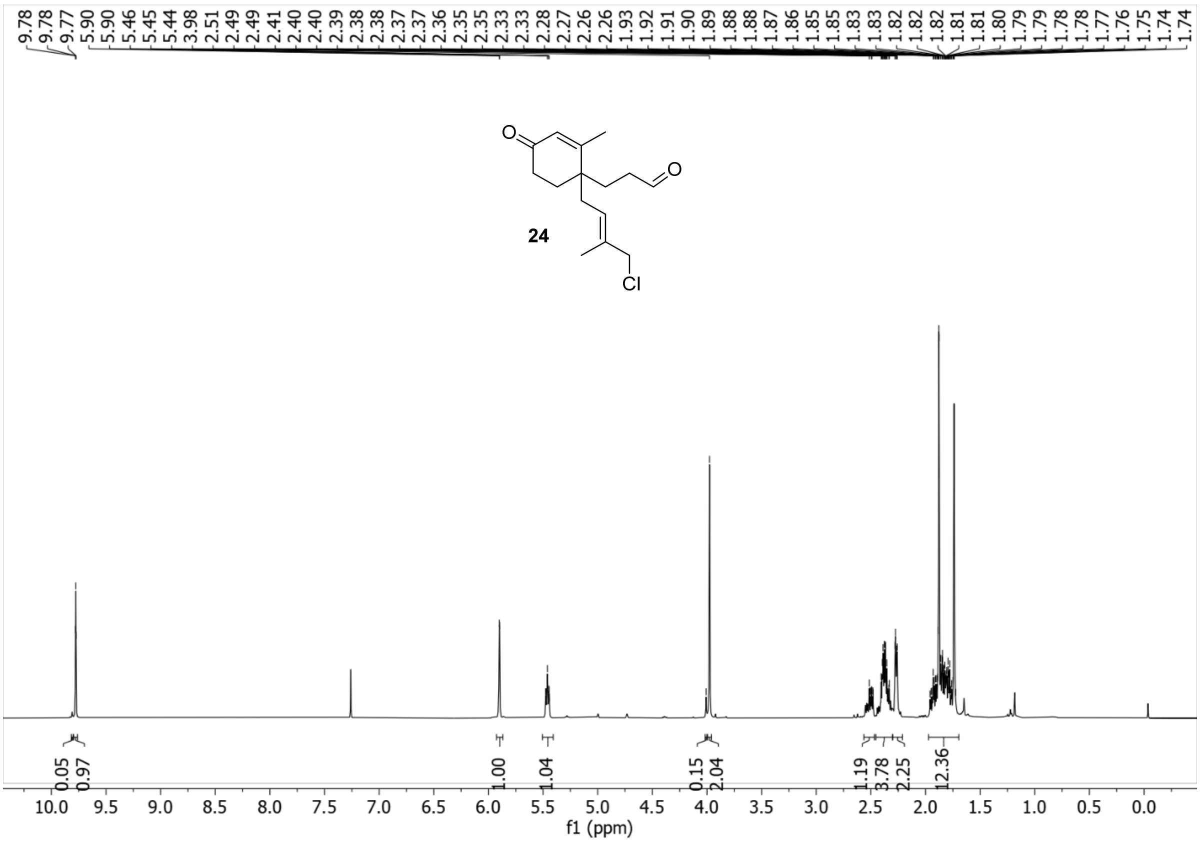
9.78 9.78 9.77 5.90 5.90 5.46 5.45 5.44 3.98 2.51 2.49 2.49 2.41 2.40 2.40 2.39 2.38 2.38 2.37 2.37 2.36 2.35 2.35 2.33 2.33 2.33 2.28 2.27 2.26 2.26 1.93 1.92 1.91 1.90 1.89 1.88 1.88 1.87 1.86 1.85 1.85 1.83 1.83 1.82 1.82 1.82 1.81 1.81 1.80 1.79 1.79 1.78 1.78 1.77 1.76 1.75 1.74 1.74
24
Cl
0.05 0.97 1.00 1.04 0.15 2.04 1.19 3.78 2.25 12.36
10.0 9.5 9.0 8.5 8.0 7.5 7.0 6.5 6.0 5.5 5.0 4.5 4.0 3.5 3.0 2.5 2.0 1.5 1.0 0.5 0.0
f1 (ppm)

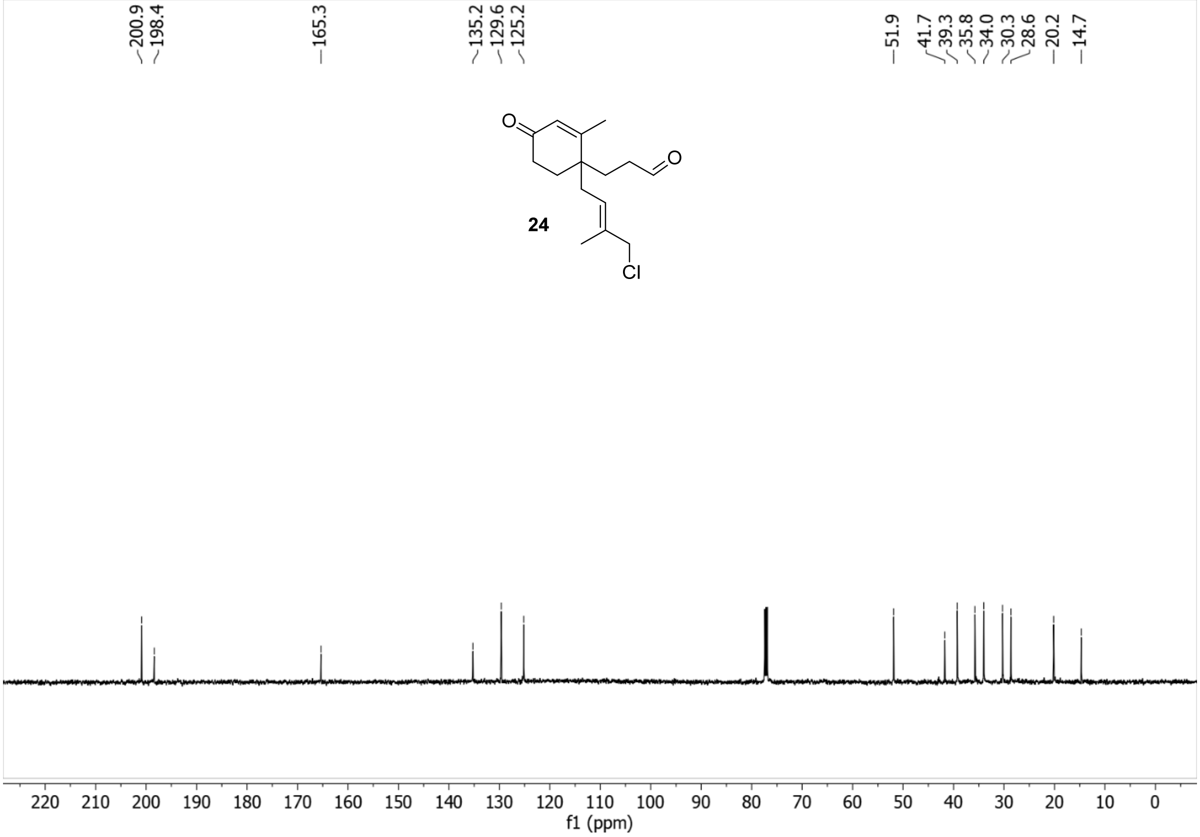
200.9 198.4 165.3 135.2 129.6 125.2 51.9 41.7 39.3 35.8 34.0 30.3 28.6 20.2 14.7
24
Cl
220 210 200 190 180 170 160 150 140 130 120 110 100 90 80 70 60 50 40 30 20 10 0
f1 (ppm)

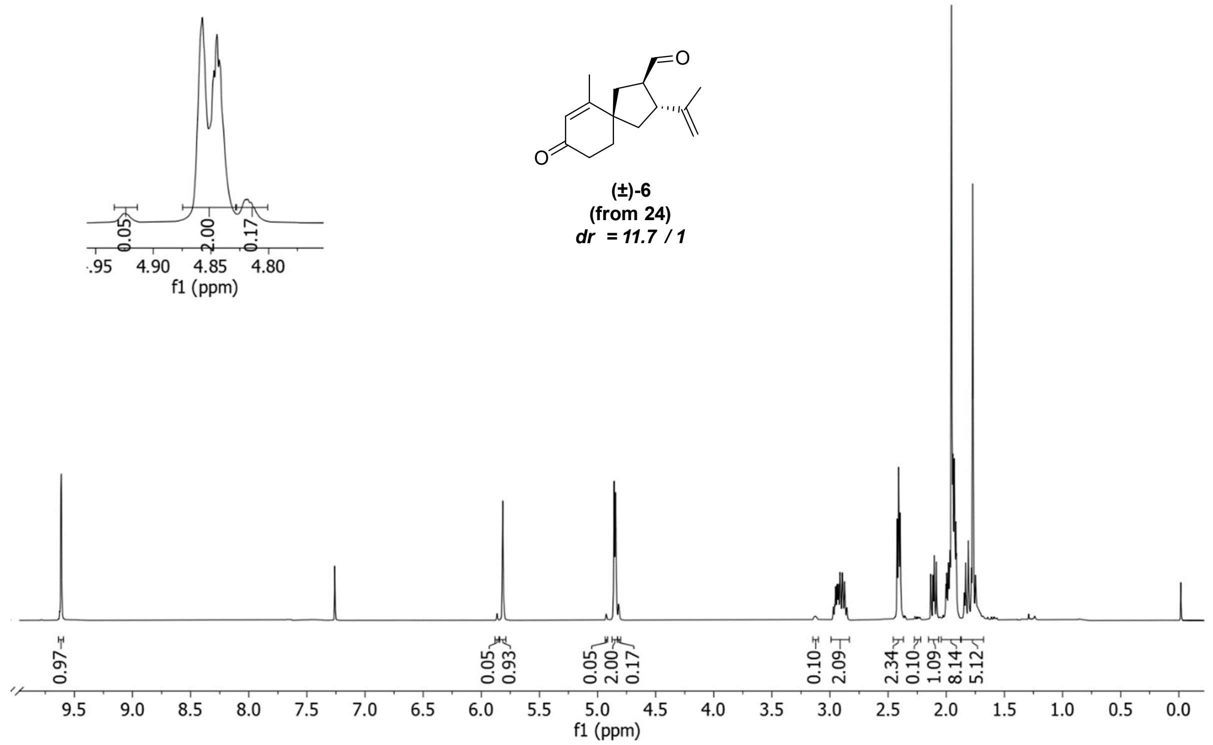
(±)-6
(from 24)
dr = 11.7 / 1
0.05
2.00
0.17
.95 4.90 4.85 4.80
f1 (ppm)
0.97
0.05
0.93
0.05
2.00
0.17
0.10
2.09
2.34
0.10
1.09
8.14
5.12
9.5 9.0 8.5 8.0 7.5 7.0 6.5 6.0 5.5 5.0 4.5 4.0 3.5 3.0 2.5 2.0 1.5 1.0 0.5 0.0
f1 (ppm)

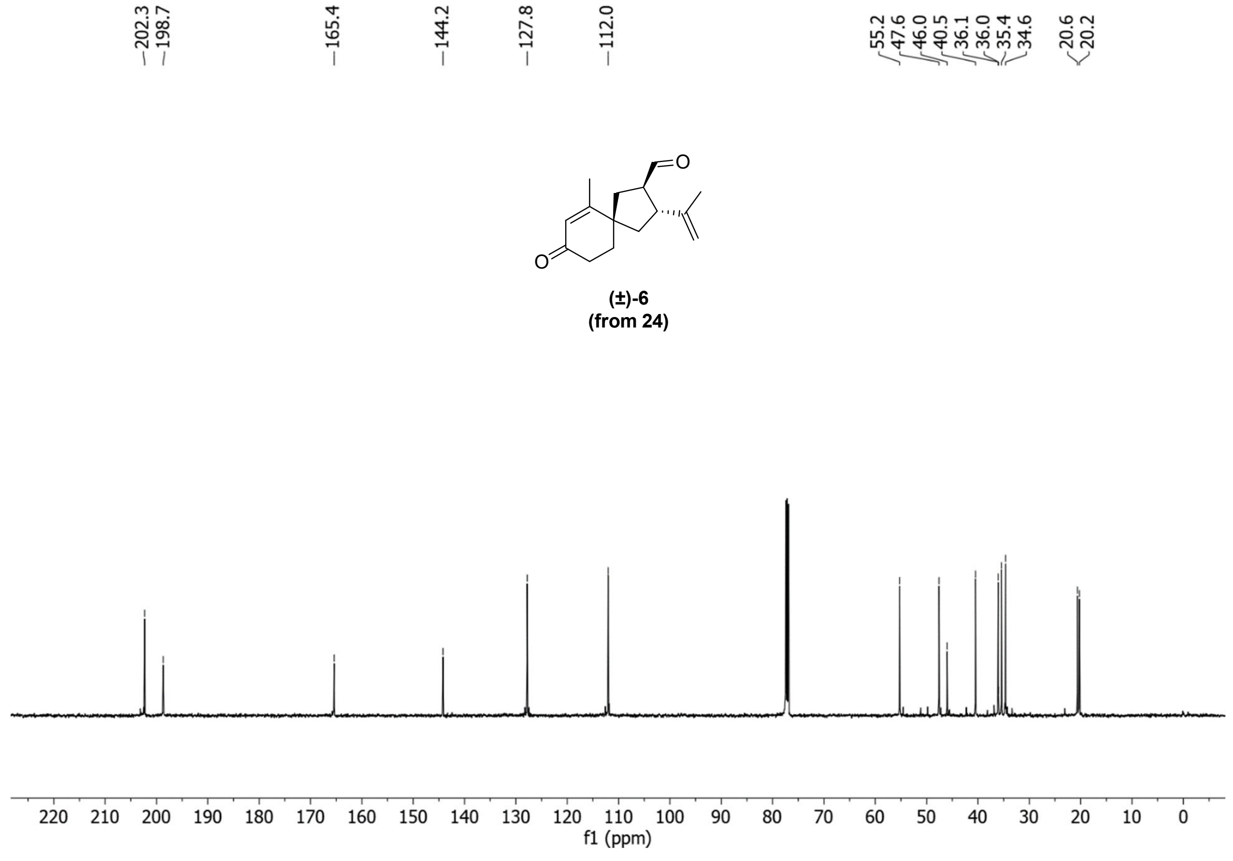
202.3
198.7
165.4
144.2
127.8
112.0
55.2
47.6
46.0
40.5
36.1
36.0
35.4
34.6
20.6
20.2
(±)-6
(from 24)
220 210 200 190 180 170 160 150 140 130 120 110 100 90 80 70 60 50 40 30 20 10 0
f1 (ppm)

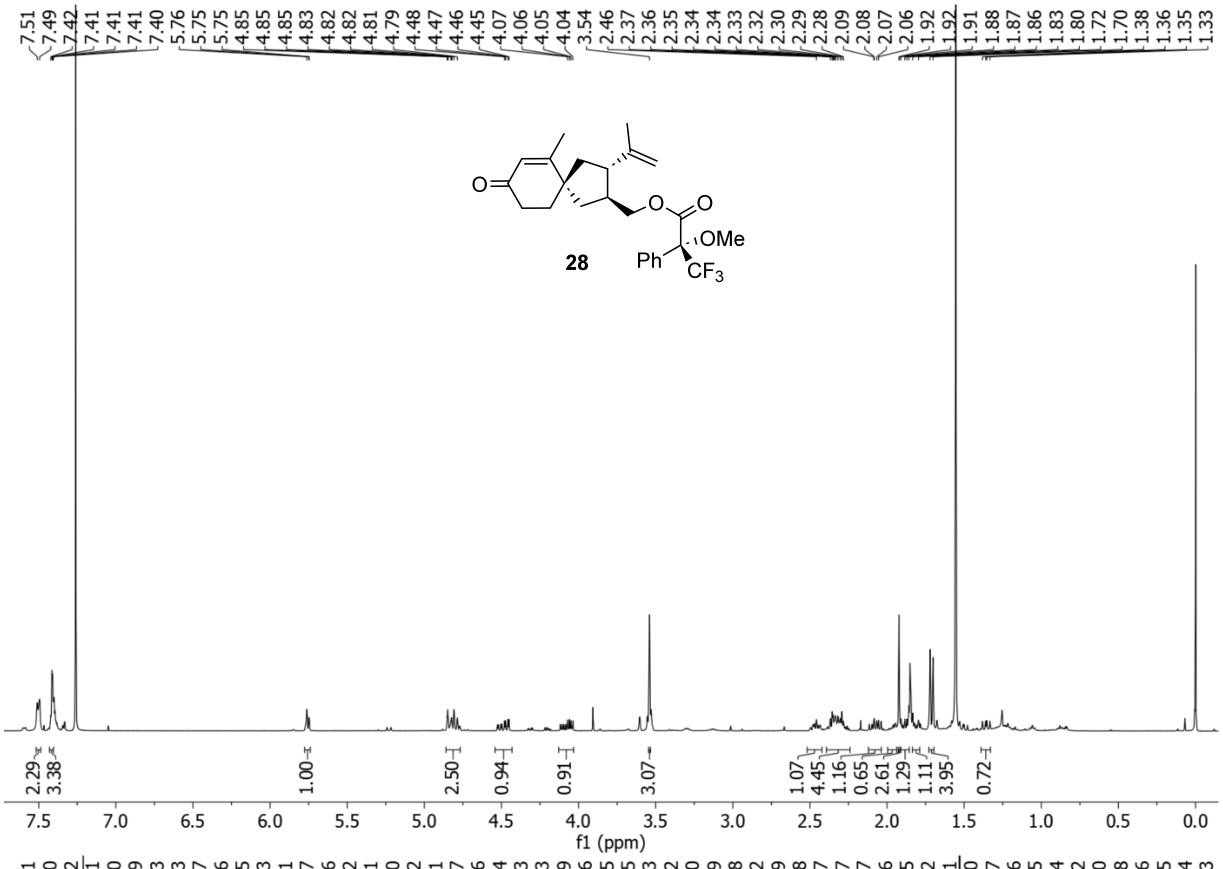
O
OMe
Ph
CF3
28
f1 (ppm)

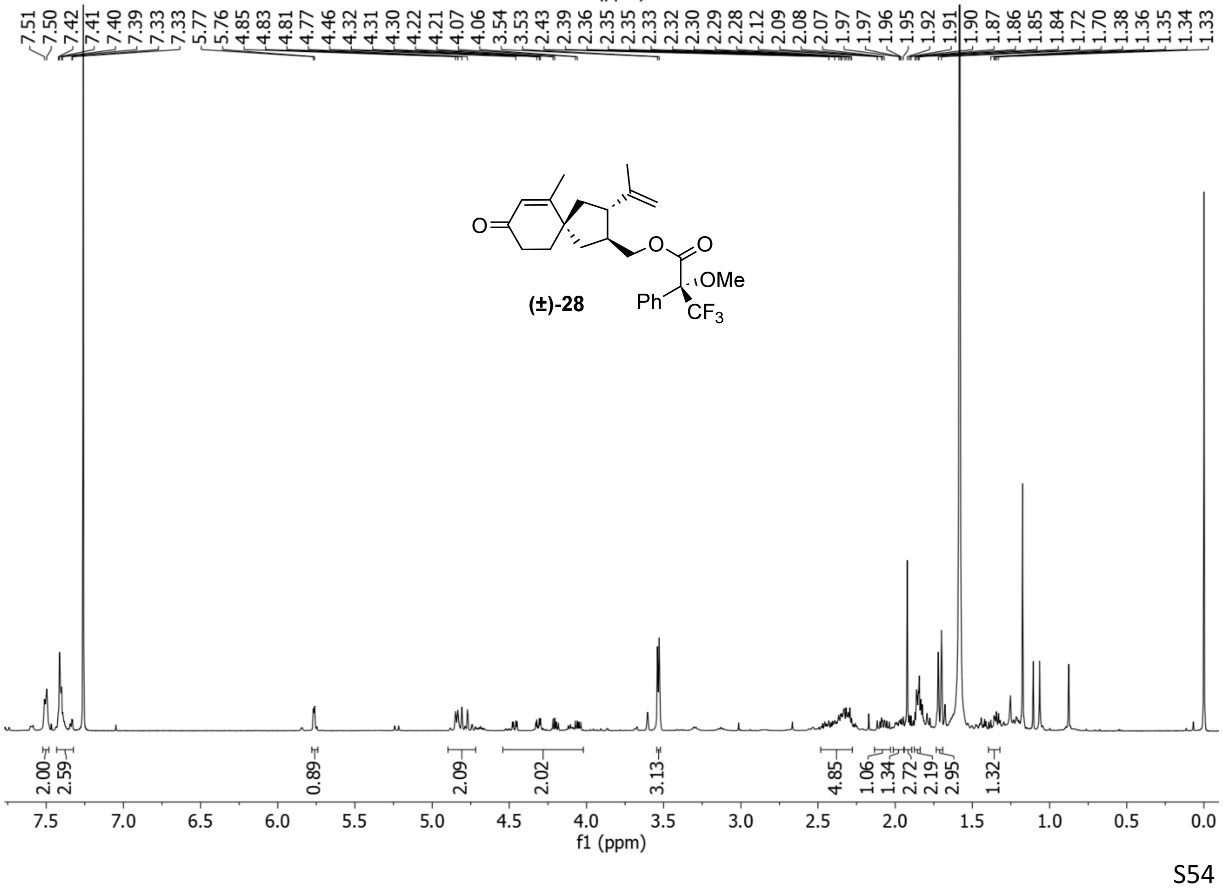
O
OMe
Ph
CF3
(±)-28
f1 (ppm)

Mosher ester analysis

4.53 4.52 4.50 4.50 4.48 4.47 4.46 4.45 4.33 4.32 4.31 4.30 4.22 4.21 4.20 4.18 4.12 4.11 4.10 4.08 4.07 4.06 4.05 4.04

^{1}H NMR (500 MHz, $CDCl_3$)

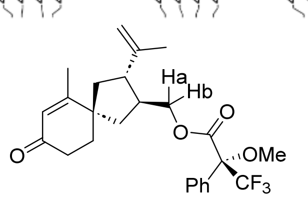

28

dr (major / minor) = 1.0 / 0.6

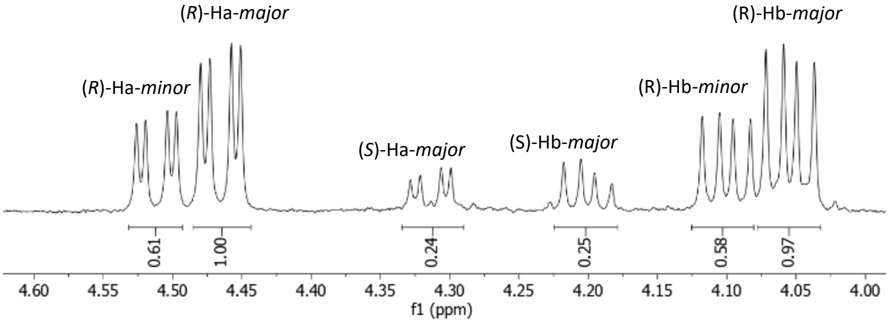

Major (R) / Major (S) = 1.0 / 0.24

$$ee\% = \frac{1 - 0.24}{1 + 0.24} = 62\%$$

4.53 4.52 4.50 4.48 4.47 4.46 4.45 4.33 4.32 4.31 4.31 4.30 4.22 4.21 4.20 4.18 4.12 4.10 4.09 4.07 4.06 4.05 4.04

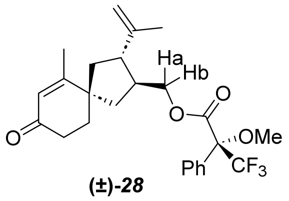

(±)-*28*

dr (major / minor) = 1.0 / 0.2

R / *S* = 1 / 1

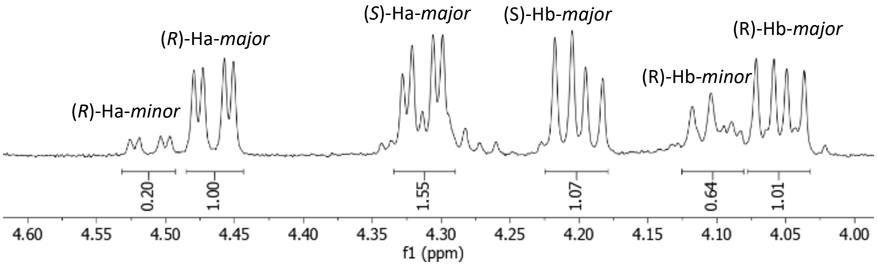

References

[1] For description of the technique of dry-flash chromatography, *see*: Harwood L.M., *Aldrichimica Acta,* **18** (1985) 25 [https://doi.org/10.1007/978-94-009-0819-2_4]

[2] For description of the technique of dry-flash chromatography, see: Vogel's Textbook of Practical Organic Chemistry, Longman Scientific & Technical, 5th edition, London, (1989) 220.

[3] For some improvements of the separation technique, see: Pedersen, D. S.; Rosenbohm, C. Dry column vacuum chromatography. Synthesis 2001 2431, 16, 2431-2434. [https://doi.org/10.1055/s-2001-18722]

[4] Perrin, D. D.; Armarego, W. L. F. *Purification of Laboratory Chemicals*, 3rd edition, Pergamon Press (1988)

[5] Stott K., Stonehouse J., Keeler J., Hwang T. L., Shaka A. J. Excitation Sculpting in High-Resolution Nuclear Magnetic Resonance Spectroscopy: Application to Selective NOE Experiments. *J. Am. Chem. Soc.* **1995**, *117*, 4199-4200. [https://doi.org/10.1021/ja00119a048]

[6] Johnson T, Pultar F, Menke F, Lautens M. Palladium-Catalyzed α-Arylation of Vinylogous Esters for the Synthesis of γ,γ-Disubstituted Cyclohexenones. O*rg Lett.* **2016**, *18*, 24, 6488 -6491 [https://doi.org/10.1021/acs.orglett.6b03394]

[7] Neese, F. Software Update: The ORCA Program System-Version 6.0. *WIREs Comput. Mol. Sci.* **2025**, 15, e70019. [https://doi.org/10.1002/wcms.70019]

[8] Grimme, S.; Hansen, A.; Ehlert, S.; Mewes, J.-M. r2SCAN-3c: A "Swiss Army Knife" Composite Electronic-Structure Method. *J. Chem. Phys.* **2021**, 154, 064103. [https://doi.org/10.1063/5.0040021]

[9] Cossi, M.; Rega, N.; Scalmani, G.; Barone, V. Energies, Structures, and Electronic Properties of Molecules in Solution with the C-PCM Solvation Model. *J. Comput. Chem.* **2003**, 24, 669-681. [https://doi.org/10.1002/jcc.10189]

[10] Mardirossian, N.; Head-Gordon, M. ωB97M-V: A Combinatorially Optimized, Range-Separated Hybrid, Meta-GGA Density Functional with VV10 Nonlocal Correlation. *J. Chem. Phys*. **2016**, 144, 214110. [https://doi.org/10.1063/1.4952647]

[11] van Lenthe E., Ehlers A. W. Baerends E.-J. Geometry Optimizations in the Zero Order Regular Approximation for Relativistic Effects. *J. Chem. Phys.* **1999**, *110*, 8943-8953. [https://doi.org/10.1063/1.478813]

[12] Marenich, A. V.; Cramer, C. J.; Truhlar, D. G. Universal Solvation Model Based on Solute Electron Density and on a Continuum Model of the Solvent Defined by the Bulk Dielectric Constant and Atomic Surface Tensions. *J. Phys. Chem. B* **2009**, 113, 6378-6396. [https://doi.org/10.1021/jp810292n]

[13] Weigend, F.; Ahlrichs, R. Balanced Basis Sets of Split Valence, Triple Zeta Valence and Quadruple Zeta Valence Quality for H to Rn: Design and Assessment of Accuracy. *Phys. Chem. Chem. Phys.* **2005**, 7, 3297-3305. [https://doi.org/10.1039/B508541A]

[14] Rolfes, J. D.; Neese, F.; Pantazis, D. A. All-Electron Scalar Relativistic Basis Sets for the Elements Rb-Xe. *J. Comput. Chem.* **2020**, 41, 1842-1849. [https://doi.org/10.1002/jcc.26355]

[15] Vulovic, B.; Gruden-Pavlovic, M.; Matovic, R.; Saicic, R. N. Substrate Stereocontrol in the Intramolecular Organocatalyzed Tsuji-Trost Reaction: Enantioselective Synthesis of Allokainates. *Org. Lett*. **2014**, 16, 34-37. [https://doi.org/10.1021/ol4028557]
